# Whole-genome duplication shaped cell-type evolution in the vertebrate brain

**DOI:** 10.1038/s41586-026-10629-x

**Published:** 2026-06-10

**Authors:** Yuanzhen Zhu, Shuai Zhang, Jiankai Wei, Diego Dolgetta-Garcia, Katia Jindrich, Huimin Liu, Chenggang Shi, Rongrong Pan, Yuwei Chen, Yan Xu, Qiye Li, Günter P. Wagner, Peter W. H. Holland, Guang Li, Sebastian M. Shimeld

**Affiliations:** 1 Department of Biology, University of Oxford, Oxford, UK; 2 State Key Laboratory of Cellular Stress Biology, School of Life Sciences, Xiamen University, Xiamen, China; 3 Fang Zongxi Center for Marine EvoDevo, MoE Key Laboratory of Marine Genetics and Breeding, College of Marine Life Sciences, Ocean University of China, Qingdao, China; 4 MoE Key Laboratory of Evolution and Marine Biodiversity, Institute of Evolution and Marine Biodiversity, Ocean University of China, Qingdao, China; 5 Mountain Ecological Restoration and Biodiversity Conservation Key Laboratory of Sichuan Province, Chengdu Institute of Biology, Chinese Academy of Sciences, Chengdu, China; 6 China–Croatia Belt and Road Joint Laboratory on Biodiversity and Ecosystem Services, Chengdu Institute of Biology, Chinese Academy of Sciences, Chengdu, China; 7 Department of Basic Medical Sciences, Qiannan Medical College for Nationalities, Duyun, China; 8 State Key Laboratory of Genome and Multi-Omics Technologies and Shenzhen Key Laboratory of Forensics, BGI Research, Shenzhen, China; 9 BGI Research, Wuhan, China; 10 Department of Ecology and Evolutionary Biology, Yale University, New Haven, CT USA; 11 Department of Evolutionary Biology, University of Vienna, Vienna, Austria

**Keywords:** Molecular evolution, Genomics, Evolutionary developmental biology, Cellular neuroscience

## Abstract

The complex brains of vertebrates have more cell types than those of their closest relatives. Whole-genome duplications (WGDs) occurred during early vertebrate evolution^1^, but it is unclear whether the duplicated genes (ohnologues) facilitated cell-type evolution. Here using brain single-cell transcriptomes from five chordates—human^2^, mouse^3^, lizard^4^, lamprey^5^ and amphioxus—we report that many cell-type families with conserved core transcription factors in vertebrates do not show one-to-one homology with amphioxus. Moreover, ohnologues, particularly those from the first WGD, were more important than small-scale duplication paralogues for vertebrate cell-type evolution. To explore whether ohnologues are mechanistically important for this process, we predicted ancestral cell-type states and compared them to amphioxus and experimentally investigated macroglia. The findings indicate that ohnologues had a role in early vertebrate cell-type diversification. Moreover, by examining paralogue expression across cell types and species, we show that expression changes were mainly driven by dosage selection and subfunctionalization. We also link ohnologues to cellular diversity at different anatomical and cell-type scales. Our findings demonstrate the importance of WGDs for the evolution of early vertebrate brain complexity and highlight that the resultant ohnologues continued to capacitate cell-type evolution long after they were formed.

## Abstract (web summary)

Analyses of brain single-cell transcriptomes from human, mouse, lizard, lamprey and amphioxus reveal that duplicated genes (ohnologues) played a pivotal part in early vertebrate cell-type diversification.

## Main

**Fig. 1:**
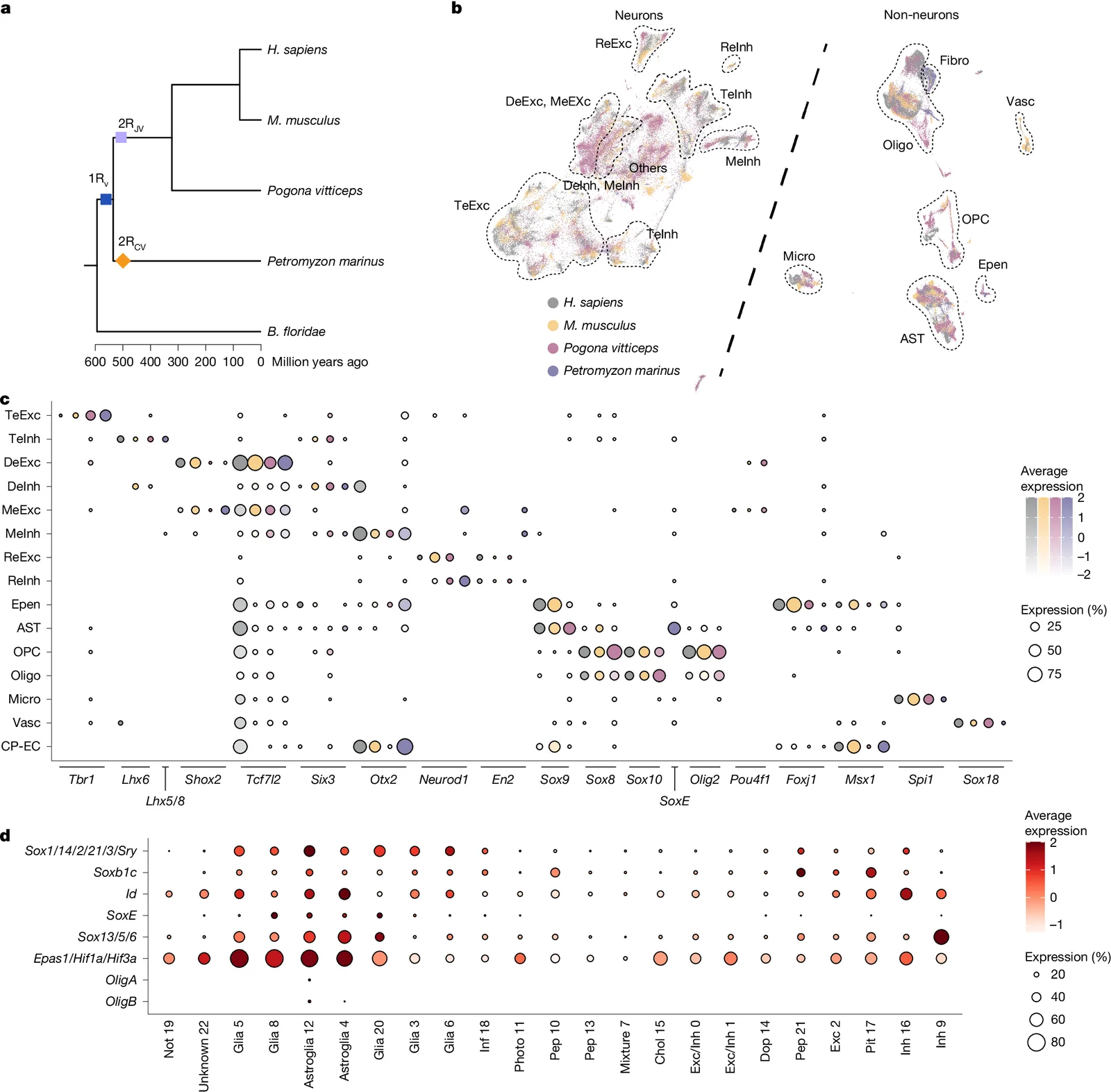
Vertebrate brain atlases and core TF programs that define major cell-type families. **a**, Phylogenetic tree showing the approximate timing (million years ago) for vertebrate-shared auto-tetraploidization (1R_V_), a jawed-vertebrate-specific allo-tetraploidization (2R_JV_) and a cyclostome-specific hexaploidization (2R_CV_) based on recent studies^1,7,45^. **b**, Uniform manifold approximation and projection (UMAP) visualization of the integrated neuronal (left) and non-neuronal (right) atlases for the four indicated species. Each dot represents a single nucleus or cell. To ensure balanced representation across datasets, only 20,000 randomly sampled cells or nuclei are shown per species for both neuronal and non-neuronal integrated atlases. **c**, Dot plot showing conserved TFs that define major cell-type families in vertebrates (a complete list is provided in Supplementary Table 3). The dot size represents the percentage of cells in each cell-type family expressing that gene. The colour gradient for each dot is a scaled average expression for each gene in the species (species colours are as in **b**). For TF gene families with multiple copies in lamprey, only the copy with the highest expression is displayed. **d**, Dot plot showing the expression of conserved key TF families of vertebrate astrocyte clusters (*x *axis) in adult amphioxus brain. The dot size represents the percentage of cells in each cell type expressing that gene. Colour represents the scaled average expression for each gene. A complete dot plot for amphioxus expression of key TFs for all vertebrate cell-type families is shown in Extended Data Fig. 3. AST, astrocytes; CP-EC, choroid plexus epithelial cells; DeExc, diencephalon glutamatergic neurons; DeInh, diencephalon GABAergic neurons; Epen, ependymal cells; Fibro, fibroblasts; MeExc, mesencephalon glutamatergic neurons; MeInh, mesencephalon GABAergic neurons; Micro, microglia; Oligo, oligodendrocytes; OPC, oligodendrocyte precursor cells; ReExc, rhombencephalon glutamatergic neurons; ReInh, rhombencephalon GABAergic neurons; TeExc, telencephalon glutamatergic neurons; TeInh, telencephalon GABAergic neurons; Vasc, vascular cells. Additional information on cluster names is provided in Supplementary Table 2.

It has long been proposed^6^ that vertebrates underwent WGDs sometime in their ancestry. This idea was later refined into a ‘2R hypothesis’, with two WGDs identified in the early evolution of jawed vertebrates. Recent studies have shown that the first WGD predated the separation of cyclostome and gnathostome lineages, and subsequent WGDs occurred independently in each lineage^1,7^ (Fig. 1a). WGD is not the only way genes duplicate and must be distinguished from extensive small-scale duplications (SSDs)^8^. Most duplicated genes are lost after duplication, but retained genes may undergo complementary loss of function (subfunctionalization) and/or evolve new functions (neofunctionalization)^9^. Retained genes are also frequently co-opted into evolving gene regulatory networks^10,11^, and this process is proposed to drive new uses in the development and specification of tissues, organs and cell types^11,12^. An evolutionary definition of cell types has been proposed that is based on common descent regardless of form and function^13^. New cell types can evolve through duplication and divergence (the sister cell-type model), which is an inherently hierarchical concept^13,14^. Consequently, in many cases, individual cell types are species-specific or clade-specific^15^. These issues highlight the importance of investigating cell-type evolution at different hierarchical levels and across different regions of the body.

Vertebrates possess well-developed brains that enable rapid and coordinated responses to environmental stimuli and facilitated adaptation to diverse ecological niches. Compared to their closest invertebrate relatives—tunicates and amphioxus—vertebrate brains are highly regionalized and complex. Previous studies^4,16–18^ have demonstrated similarities and differences in neural cell types within and between vertebrate species. However, ancestral repertoires of neural cell types and their core transcription factor (TF) programs, and the origin of cell types in early vertebrates, remain poorly understood. Potential roles for WGD and SSD paralogues in brain cell-type evolution remain obscure. In this study, we analyse four vertebrate (human (*Homo sapiens*), mouse (*Mus musculus*), lizard (*Pogona vitticeps*) and lamprey (*Petromyzon marinus*)) and one amphioxus (*Branchiostoma floridae*) whole brain single-cell transcriptomes to infer ancestral repertoires of neural cell-type families. We then systemically analyse WGD paralogues (ohnologues) and SSD paralogues in cell-type evolution. Our findings indicate that 2R WGDs capacitated cell-type innovation during both early vertebrate evolution and over the subsequent hundreds of millions of years.

## New vertebrate brain cell-type families

To compare vertebrate brain cell types, we surveyed single-cell RNA (scRNA) and single-nucleus RNA (snRNA) data from four vertebrates: human, mouse, lizard and lamprey^2–5^. Data were filtered to retain only brain tissues at juvenile or adult stages. To balance cell numbers, we downsampled human and lizard atlases, but retained full atlases for mouse and lamprey (Methods). For amphioxus (outgroup) brain, we generated 3,217 neurons and 2,351 non-neuronal cells (Supplementary Fig. 1). We reanalysed each species using self-assembling manifold (SAM)^19^, then performed iterative clustering (except for amphioxus; Methods). This process resulted in 241, 167, 202, 141 and 23 clusters for human, mouse, lizard, lamprey and amphioxus, respectively. We attributed clusters to cell types on the basis of reference annotation, marker expression (Methods and Supplementary Tables 1 and 2) and SAMap^20^ mapping (see below). Overall, 94% of clusters on average were reliably assigned to cell types in the 4 vertebrates and 21 out of 23 in amphioxus (Supplementary Table 2).

**Extended Data Fig. 1:**
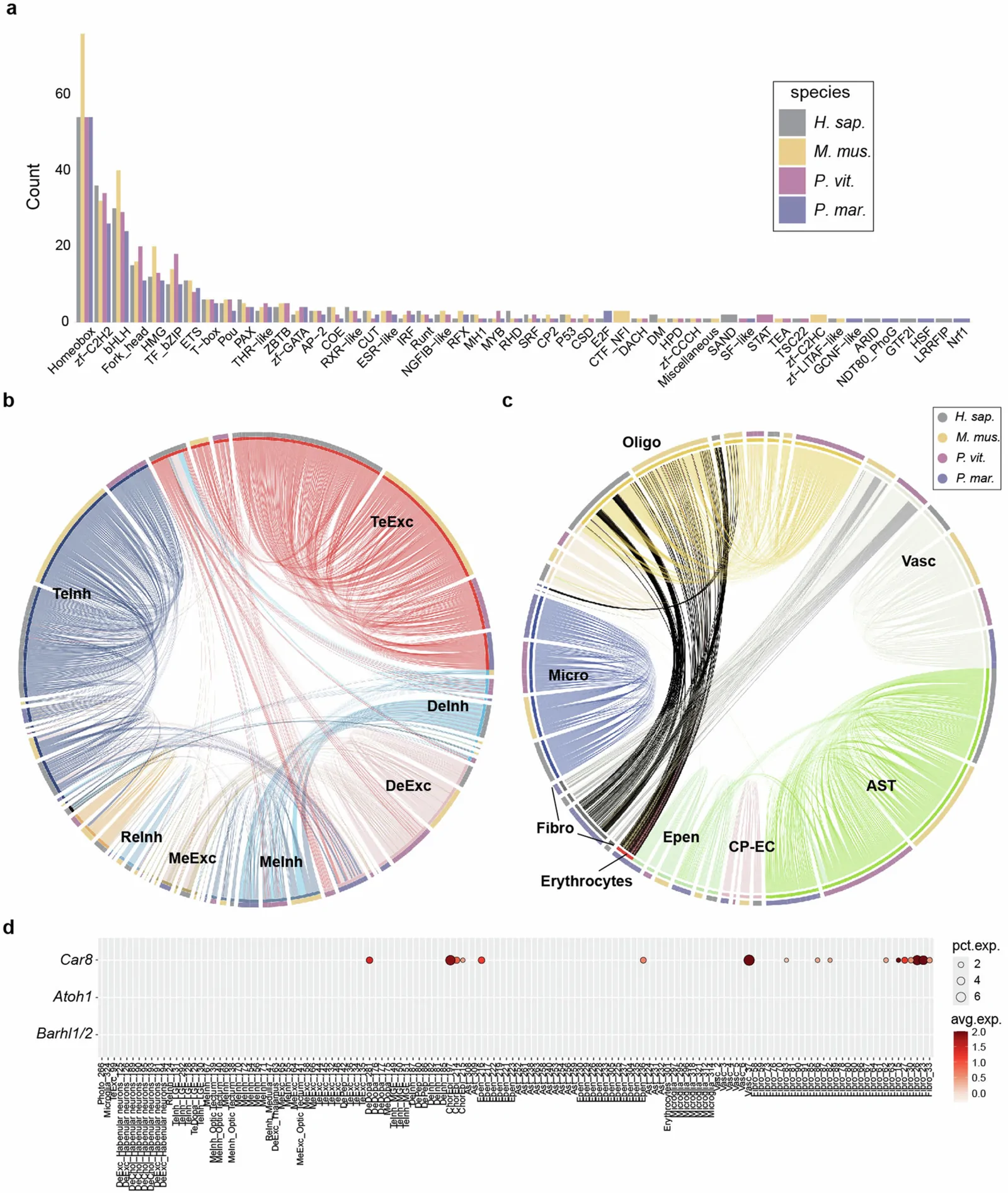
Categories of shared TFs and cell type mapping across vertebrates. **a**, Bar plot shows the number of conserved TFs of major cell-type families across TF families. TF genes were classified based on the classification scheme which was downloaded from website (https://guolab.wchscu.cn/AnimalTFDB4//#/Family). The colour of bar represents different species. The height of bar is the number of TFs classified as a certain type. **b,c** Chord plots displaying mappings within neuronal atlas (**b**) and non-neuronal atlas (**c**) across vertebrates. The outer coloured circle represents different species. The inner coloured circle denotes the colour of major cell-type families. Major cell-type families were also bold labelled as abbreviations, as in Fig. 1. False mappings from fibroblasts and lamprey erythrocytes to amniote oligodendrocytes were coloured as black lines. **d**, Dot plot of cerebellar markers in lamprey with dot size as the percentage of cells expressing that gene. Dot colour reflects the scaled average expression (−2 to 2) across all clusters. Dots representing clusters with <1% of cells expressing the gene were omitted to reduce noise. Purkinje cell marker: *Car8*; Cerebellar granule cell markers: *Math1* and *Barhl1*).

Evolutionarily, cell types can emerge through duplication and divergence^13,14^ in a manner conceptually similar to gene duplications that form lineage-specific paralogues^15^. This concept means that one-to-one cell-type homology at high resolution might not exist between distant species^15^. Nevertheless, the conservation of brain regionalization and its developmental basis^21^ led us to focus on cell-type families at the same hierarchical layer. Cell-type families can be defined as a set of cell types which use the same regulatory programs that drive differentiation and identity^22^ (for example, as defined by core regulatory complexes (CoRCs)^13^, character identity networks (ChINs)^23^ and terminal selectors^24^). We therefore predicted cell-type family specific TFs and used conserved TFs to define cell-type families in vertebrates (Fig. 1c, Methods and Supplementary Table 3). Homeodomain TFs were the most represented TF type in cell-type-specific TFs and the only enriched class in all four species (hypergeometric test, adjusted *P* < 0.01; Methods and Extended Data Fig. 1a). This result supports previous reports of their roles as terminal selectors^24–26^.

We separated neurons and non-neurons and then performed SAMap^20^ mapping (Fig. 1b). Most clusters mapped to clusters in the same cell-type families (Extended Data Fig. 1b,c), with around 76% and 91% of bidirectional linkages connecting clusters in the same neuronal and non-neuronal families, respectively. These cell-type families were also represented by conserved TF programs (Fig. 1c and Supplementary Table 3). However, some discrepancies were noted. Lamprey erythrocytes mapped to jawed vertebrate oligodendrocytes, but with low mapping scores and supporting gene-pair numbers (median = 0.19; 157 versus 906 gene pairs on average for homologous astrocytes; Methods). Lamprey rhombencephalon GABAergic neurons shared several TFs (*Bhlhe22*,* Lbx1*,* Lhx1, Lhx5*,* Neurodo1*,* Neurodo2*,* En2*,* Hoxb3*,* Tfap2a* and* Tfap2b*) with jawed vertebrate counterparts. However, mapping relationships between lamprey rhombencephalon and jawed vertebrate rhombencephalon cells were unstable, and expression of some rhombencephalon cell-type markers was absent (Extended Data Fig. 1d). This finding is consistent with previous works^5,27,28^.

**Extended Data Fig. 2:**
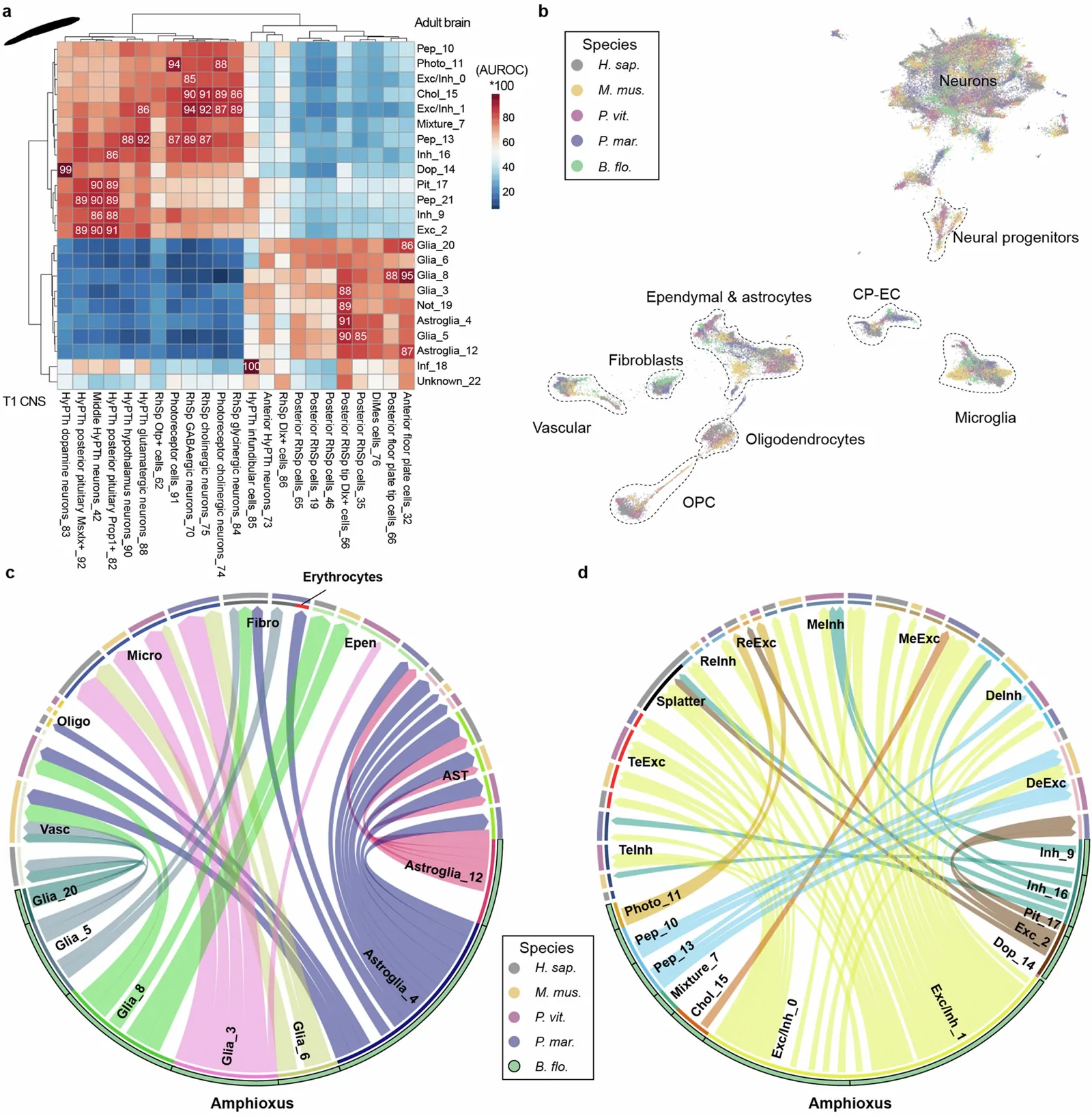
Amphioxus cell-types mapping. **a**, Heatmap showing mapping between T1 (late neurula) CNS cell types and adult brain cell types in amphioxus. The colour represents pairwise AUROC scores by MetaNeighbor and AUROC scores > 0.85 are presented with 100*score in heatmap for visualisation convenience. **b**, UMAP visualization of the integrated five chordate cell atlases. Each dot represents a single nucleus or cell. Different colours indicate different species. Amphioxus cells are plotted on the top layer of the UMAP to better show their locations. **c,d** Chord plots displaying mappings within non-neuronal and neuronal cell-types from amphioxus to four vertebrates, respectively. The outer coloured circle represents different species, with the same colour as in (**b**). The inner coloured circle denotes the colour of major cell-type families. Major cell-type families of vertebrates and cell-types of amphioxus are bold labelled with their abbreviations.

**Extended Data Fig. 3:**
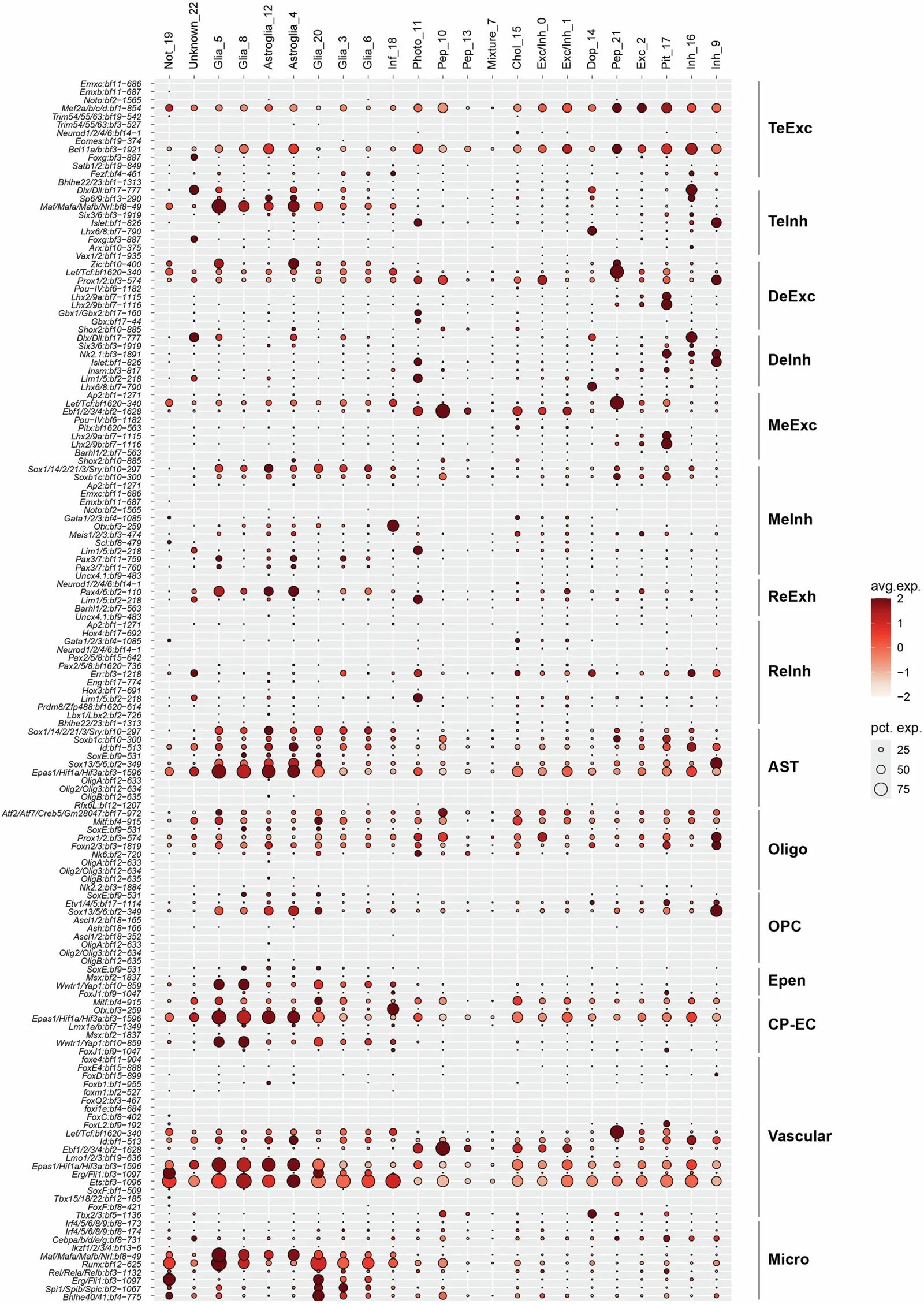
Expression of conserved key TFs for vertebrate major cell-type families in adult amphioxus brain. Gene annotation and ID are presented on the left-hand side. Vertebrate major cell-type families were labelled on the right side. Dot size indicates the percentages of cells within each cluster expressing that gene (pct. exp), while dot colour reflects the scaled average expression (−2 to 2) across all clusters. Dots representing clusters with <1% of cells expressing the gene were omitted to reduce noise.

Amphioxus cell annotation was cross-validated against embryonic scRNA-seq data^29^ (Extended Data Fig. 2a). To compare amphioxus and vertebrates, we performed SAMap mapping across five chordates (Methods and Extended Data Fig. 2b). Amphioxus astroglia mapped to vertebrate macroglia (ependymal cells, astrocytes and oligodendrocytes). Most amphioxus neurons did not map to a single vertebrate cell-type family but showed broad pan-mapping (Extended Data Fig. 2c,d). Vertebrate cell-type family conserved TFs did not show strong expression specificity in amphioxus (Fig. 1d and Extended Data Fig. 3). Notably, amphioxus glia did show stronger expression of astrocyte TFs, which suggests that they share a degree of ‘primitive’ identity but with later macroglia specialization in vertebrates. We considered whether whole-brain comparisons might be affected by derived increases in size and complexity of the vertebrate cortex. We therefore investigated the evolutionarily conserved hypothalamus. We observed functionally analogous cell types between amphioxus and mouse (dopaminergic neurons, some peptidergic neurons and neurosecretory cells). However, consistent with the outcomes of the whole-brain analyses, key TFs were different (Supplementary Text and Supplementary Figs. 3–5). In summary, these data suggest that most vertebrate brain cell-type families originated in the vertebrate stem lineage through distinct and specific TF expression and that they have been subsequently conserved.

## WGD versus SSD in cell-type diversity

**Fig. 2:**
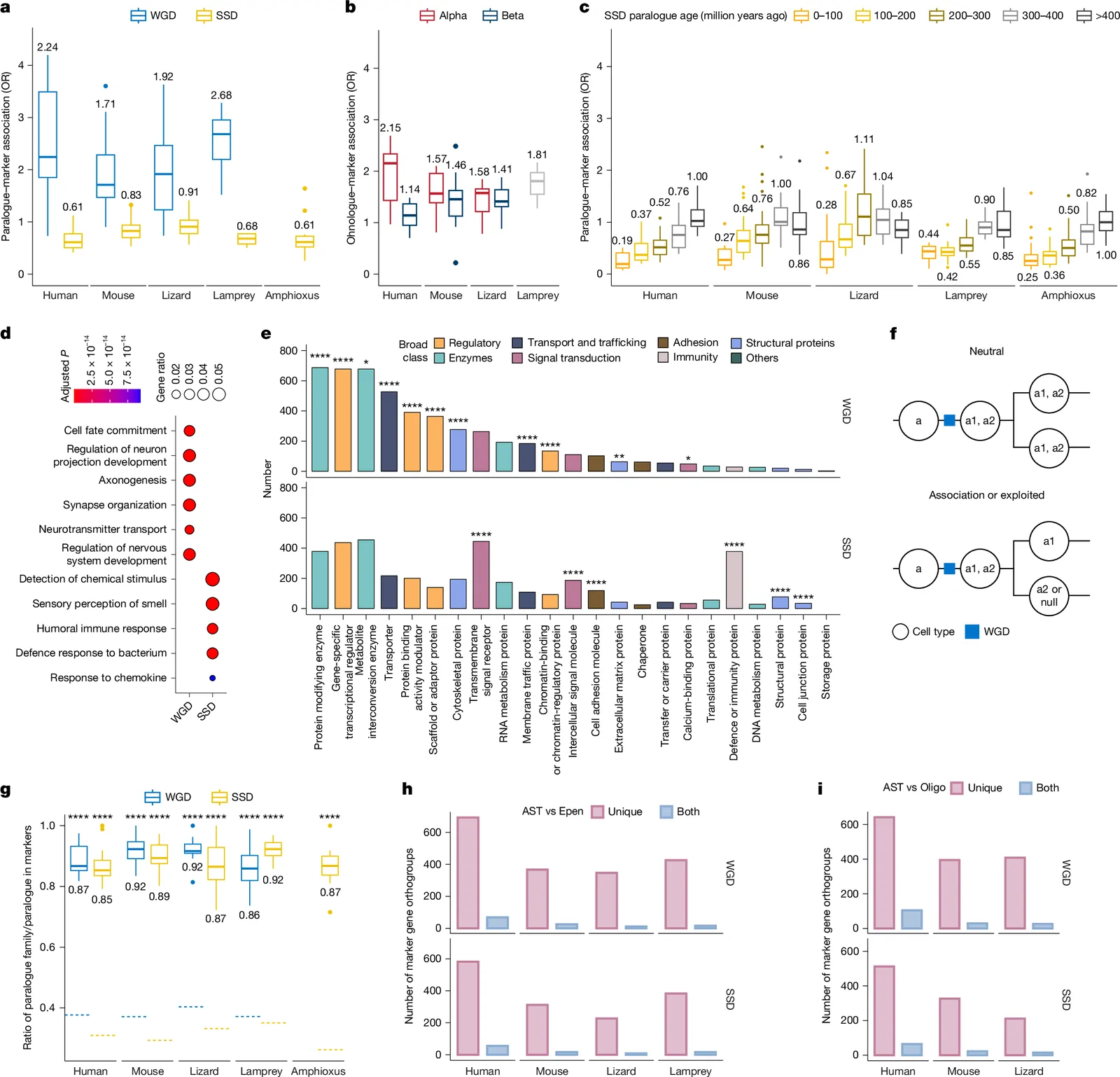
Ohnologues contributed more to cell-type evolution than SSD paralogues. **a**, ORs calculated from Fisher’s exact test on WGD (ohnologue) and SSD paralogues with cell-type family (Supplementary Table 2) DEGs. The test measures the association between WGD and SSD paralogues and DEGs. Number of cell-type families analysed: human, 17; mouse, 22; lizard, 17; lamprey, 18; and amphioxus, 15. Box plots show the median (centre line) and the interquartile range (box), with whiskers 1.5× the interquartile range. **b**, ORs from Fisher’s exact tests on alpha and beta ohnologues with cell-type family DEGs. The cell-type families analysed and box plot elements are as for **a**. **c**, ORs from Fisher’s exact tests on age-stratified SSD paralogues with cell-type family DEGs. The cell-type families analysed and box plot elements are as for **a**. **d**, Selected top enriched GO terms (by clusterProfiler; Methods) of WGD and SSD paralogues in humans. The circle colour denotes the false-discovery rate (FDR) range, whereas the size represents the gene ratio. *n* = 5,907 WGD paralogue genes and* n* = 4,669 SSD paralogue genes expressed in the brain (background). **e**, Bar plot showing the numbers of protein classes in WGD and SSD human paralogues. The colour represents broad classifications. FDR values of overrepresented classes (by PANTHER; Methods) are shown: *P < 0.05, **P < 0.01, ***P < 0.001, ****P < 0.0001; FDRs for significantly under-represented and non-significant classes are not shown. **f**, Differences between ohnologues associated with marker neutrally (top) and differentially exploited by different cell types (bottom). The letter ‘a’ indicates markers. **g**, The ratio of WGD and SSD families that include markers to paralogues that are markers for each cell-type family. The cell-type families analysed and box plot elements are as for **a**. If only one copy in each gene family was used as a marker, the ratio would be 1. One-sample Wilcoxon signed-rank test, ****P < 0.0001. The background ratio is represented by blue (WGD) and yellow (SSD) dotted lines. **h**,**i**, Numbers of paralogue families containing markers between pairs of sister cell types: astrocytes versus ependymal cells (**h**) and astrocytes versus oligodendrocytes (**i**). ‘Unique’ means paralogue families only used by one of the sister cell types, whereas ‘both’ indicates those used by both, but different duplicates were used as markers. All statistical tests are two-sided, except in **d** and **e**.

**Extended Data Fig. 4:**
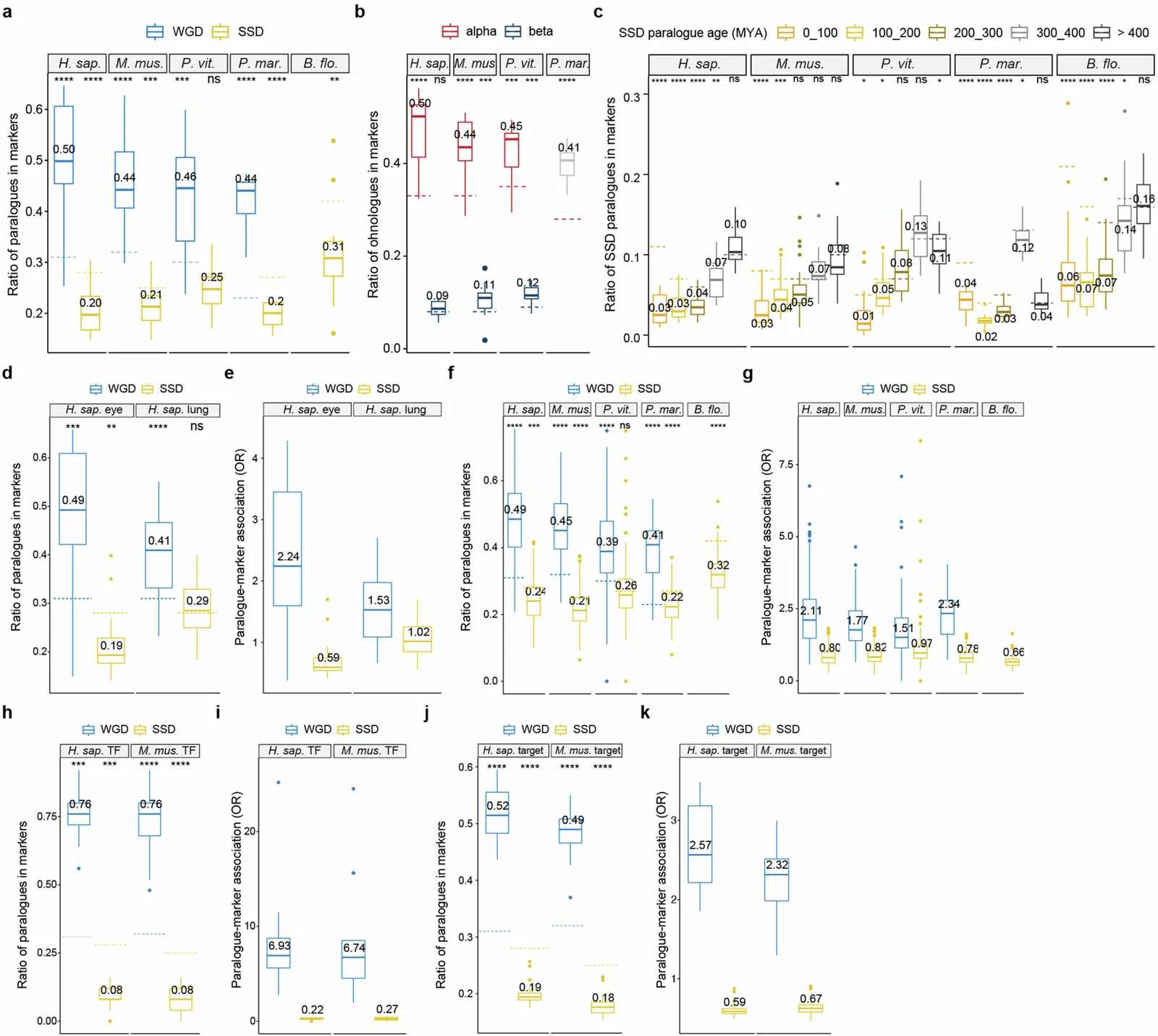
Relationships between paralogues and cell-type identity and diversity. **a**, Box plot showing the ratio of ohnologues (WGD, blue) and SSD paralogues (SSD, yellow) in cell-type family (see Supp. Table 2) DEGs. Number of cell-type families analysed per species and boxplot elements as Fig. 2a. The significance of differences between the ratio of paralogues as markers and their total percentage in all protein-coding genes was assessed using two-sided one-sample Wilcoxon signed-rank test. The ratio denotes the number of paralogue families to number of paralogues and were represented by a blue (ohnologue) and a yellow (SSD paralogue) dotted line to be used as background for each species. **b,c** Box plots displaying the ratio of ‘alpha/beta’ ohnologues (b) and age stratified SSD paralogues (c) in cell-type family DEGs. Cell-type families analysed and boxplot elements as Fig. 2a. **d**, Box plot showing the ratio of ohnologues (WGD, blue) and SSD paralogues (SSD, yellow) in cell-type DEGs from human eye (n = 18 cell types) and lung (n = 26). Boxplot elements and significance were calculated as (a). The human eye and lung scRNA were pre-processed by Human Cell Atlas and downloaded from following links (https://datasets.cellxgene.cziscience.com/64175889-d600-4b58-97ea-e74be80206e5.rds and https://datasets.cellxgene.cziscience.com/b351804c-293e-4aeb-9c4c-043db67f4540.rds). The DEGs were detected using Seurat FindAllMarkers on a subset of whole atlas with 3,000 cells per cell type and then filtered with FDR < 0.01. **e**, Odds ratio (OR) was calculated from two-sided Fisher’s exact test on ohnologues and SSD paralogues with cell-type DEGs from human eye and lung. Cell types analysed and boxplot elements as (**d**). **f,g** The relationships between DEGs and paralogues at cell-type (cluster) level. Number of clusters are described in Main text and Supp. Table 2. (**f**) paralogue ratio in TFs for each cell type, (**g**) Odds ratio calculated from two-sided Fisher’s exact test. Cell-type families analysed and boxplot elements are the same as (**a**). **h,i** The relationships between TFs from top 25 cell-type-family-specific regulons and paralogues in human and mouse. Cell-type families analysed and boxplot elements as (**a**). (**h**) paralogue ratio in TFs for each cell type, (**i**) Odds ratio calculated from two-sided Fisher’s exact test on ohnologues and SSD paralogues with above TFs. **j,k** The relationships between target genes from top 25 cell-type-family-specific regulons and paralogues in human and mouse. Cell-type families analysed and boxplot elements as (**a**). (**j**) paralogue ratio in target genes for each cell-type family, (**k**) Odds ratio calculated from two-sided Fisher’s exact test on ohnologues and SSD paralogues with above target genes. The p-value and FDR are shown as following: ns (not significant); * (*P* < 0.05); ** (*P* < 0.01); *** (*P* < 0.001); **** (*P* < 0.0001). Odds ratios are calculated from two-sided Fisher’s exact test for (e,g,i,k); p-values are calculated by two-sided one-sample Wilcoxon signed-rank test for (a-d,f,h,j).

To address how and to what extent gene duplicates contribute to cell-type diversity and evolution, we identified ohnologues and SSD paralogues (Methods; see Supplementary Text for our evaluation of ohnologue detection). This analysis generated 6,206, 6,344, 5,616 and 4,273 pooled ohnologues for human, mouse, lizard and lamprey, respectively. Moreover, 5,977, 6,265, 5,007, 6,783 and 11,083 pooled SSD paralogues for human, mouse, lizard, lamprey and amphioxus, respectively, were identified. We asked whether ohnologues or SSD paralogues were differentially expressed genes (DEGs or ‘markers’; Methods) at the cell-type family level. Enrichment analyses of paralogues and DEGs indicated that ohnologues were significantly enriched as markers in all vertebrates, whereas SSD paralogues showed the inverse in all chordates (Fig. 2a and Extended Data Fig. 4a).

SSDs arise continuously, whereas the 2R WGDs occurred in a specific evolutionary window, which means that gene duplication age might confound our analyses. Furthermore, asymmetric gene loss occurred following the jawed-vertebrate-specific WGD, which derived from interspecific hybridization from two lineages: alpha and beta^30^. We separated ohnologues into those derived from alpha and beta lineages and classified SSD paralogues on the basis of estimated duplication time (Methods). We observed the same associations between markers and ohnologues or SSD paralogues as described above (Fig. 2b,c and Extended Data Fig. 4b,c). Markers were associated more with alpha than beta ohnologues, especially for human ohnologues. SSD paralogues exhibited a negative association with markers overall. In general, recent SSDs showed more negative associations than ancient SSDs, although even the most ancient SSDs were still lower than WGDs. These findings were reinforced by similar results in other tissues and at different levels of cell types (Extended Data Fig. 4d–g, Supplementary Figs. 6 and 7 and see below). TFs and putative target genes in cell-type-specific regulons (Methods) also showed the same patterns as ohnologues and SSD paralogues (Extended Data Fig. 4h–k).

**Extended Data Fig. 5:**
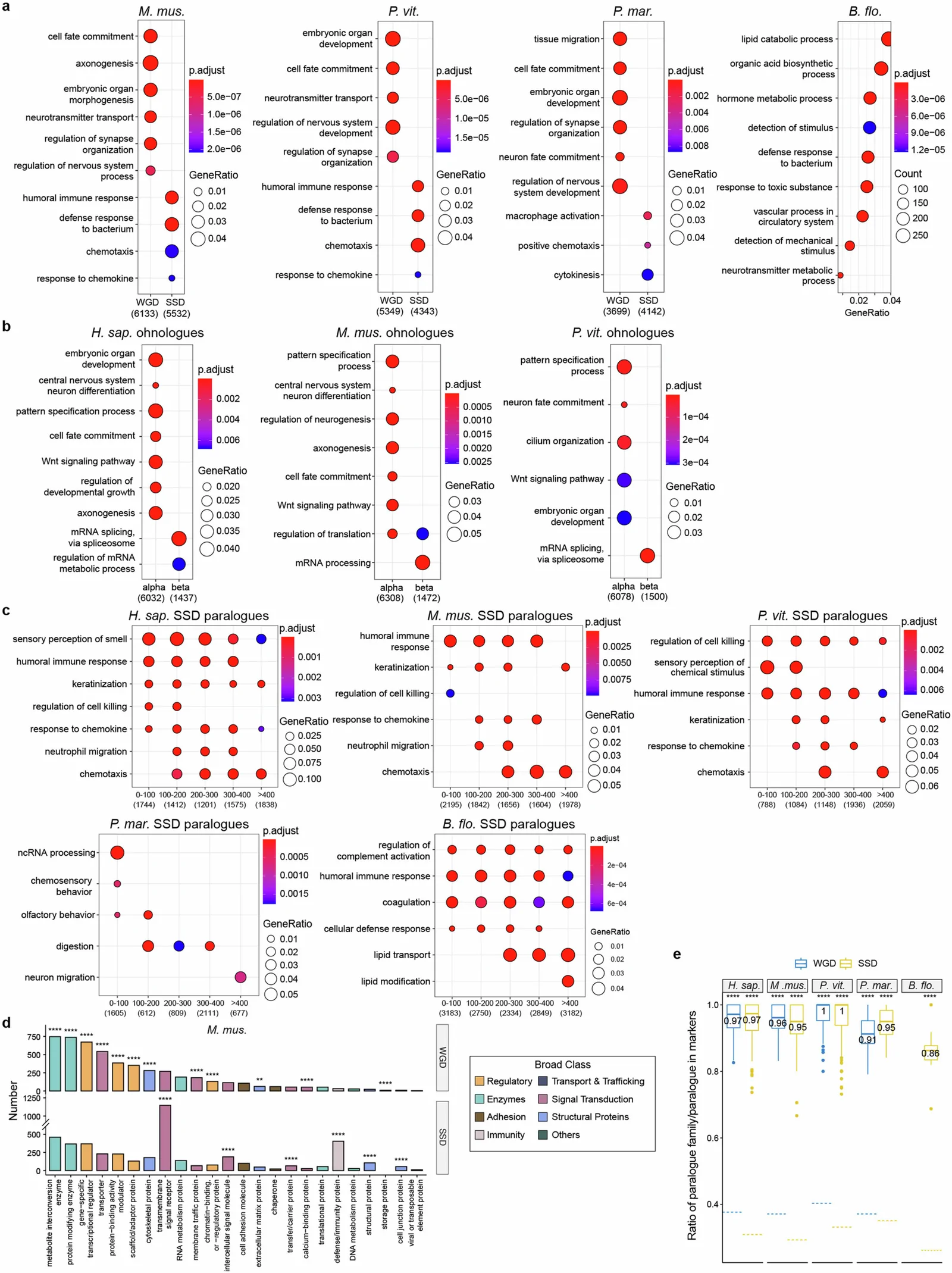
GO enrichment of ohnologues and SSD paralogues. **a**, GO enrichment analysis on ohnologues and SSD paralogues in mouse, lizard, and lamprey and paralogue enrichment in amphioxus by clusterProfiler. The colour represents FDR and dot size denotes gene ratio for vertebrates or gene count for amphioxus. **b**, GO enrichment analysis on ‘alpha’ and ‘beta’ ohnologues in human, mouse, and lizard. The colour and dot size are presented in the same way as (**a**). **c**, GO enrichment analysis on age stratified SSD paralogues for five chordates. The colour and dot size are presented in the same way as (**a**). **d**, Bar plot showing the number of different protein classes in WGD and SSD paralogues of mouse. Colour represents broad classifications. FDR values of overrepresented classes are shown based on ns (not significant); * (*P* < 0.05); ** (*P* < 0.01); *** (*P* < 0.001); **** (*P* < 0.0001) whereas FDR in under-represented classes and non-significant classes are not shown. **e**, The ratio of the number of WGD families (blue) and SSD families (yellow) that include markers to the number of paralogues that are markers for each cell type (cluster level). Number of clusters are described in Main text and Supp. Table 2 (number of clusters with significant markers, human: 211, mouse: 155, lizard: 183, lamprey: 141). Boxplot elements as Fig. 2a. If only one copy in each gene family were used as a marker, the ratio would be 1. Two-sided one-sample Wilcoxon signed-rank test, *p*-value: **** *P* < 0.0001. The background ratio denotes the number of paralogue families to number of paralogues and were represented by a blue (WGD) and a yellow (SSD) dotted line to be used as background for each species. The *p*-value is displayed as in (**d**).

To understand why markers were more associated with ohnologues than SSD paralogues, we performed gene ontology (GO) enrichment analyses (Fig. 2d,Methods and Extended Data Fig. 5a). Ohnologues were enriched in development, cell-fate commitment, signalling and neurotransmitter transport. By contrast, SSD paralogues were enriched for immune response and sensory perception in all species, a result that matched previous reports^8,31^. GO enrichment comparisons between alpha and beta ohnologues showed that alpha was the most important contributor to this (Extended Data Fig. 5b). Age-stratified SSD paralogues showed similar enrichment of immune response and chemosensory terms (Extended Data Fig. 5c). Consistently, we found that TFs, cofactors and transporters, among others, were preferentially retained following WGDs (Fig. 2e, Methods and Extended Data Fig. 5d). These data suggest that the positive association between ohnologues and markers is partially due to the preferential retention of TFs, other developmental regulation genes and some effectors, especially from alpha ohnologues.

Being associated with markers does not prove that ohnologues were leveraged for the generation of new cell types. However, if this pattern reflects the use of ohnologues to increasingly specialized cell types in vertebrate evolution, we expected that pairs of ohnologues were used in different cell types (Fig. 2f). To test this idea, for each cell-type family, we calculated the number of paralogue families having a copy (or copies) as markers and the number of paralogues as markers. The ratio between the two was close to 1, a value significantly higher than expected for both WGD and SSD paralogues (Fig. 2g). We found the same pattern at the cell-type level (Extended Data Fig. 5e). This result shows that if a paralogue is a marker for a specific cell type, its other paralogues are less likely to be markers for that cell type, regardless of duplication type and cell-type granularity. Notably, most marker paralogue families were used in only one of two sister cell types rather than different copies being used by sister cell types (Fig. 2h,i and see below for sister cell-type identification).

## Linking WGD to cell-type evolution

**Fig. 3:**
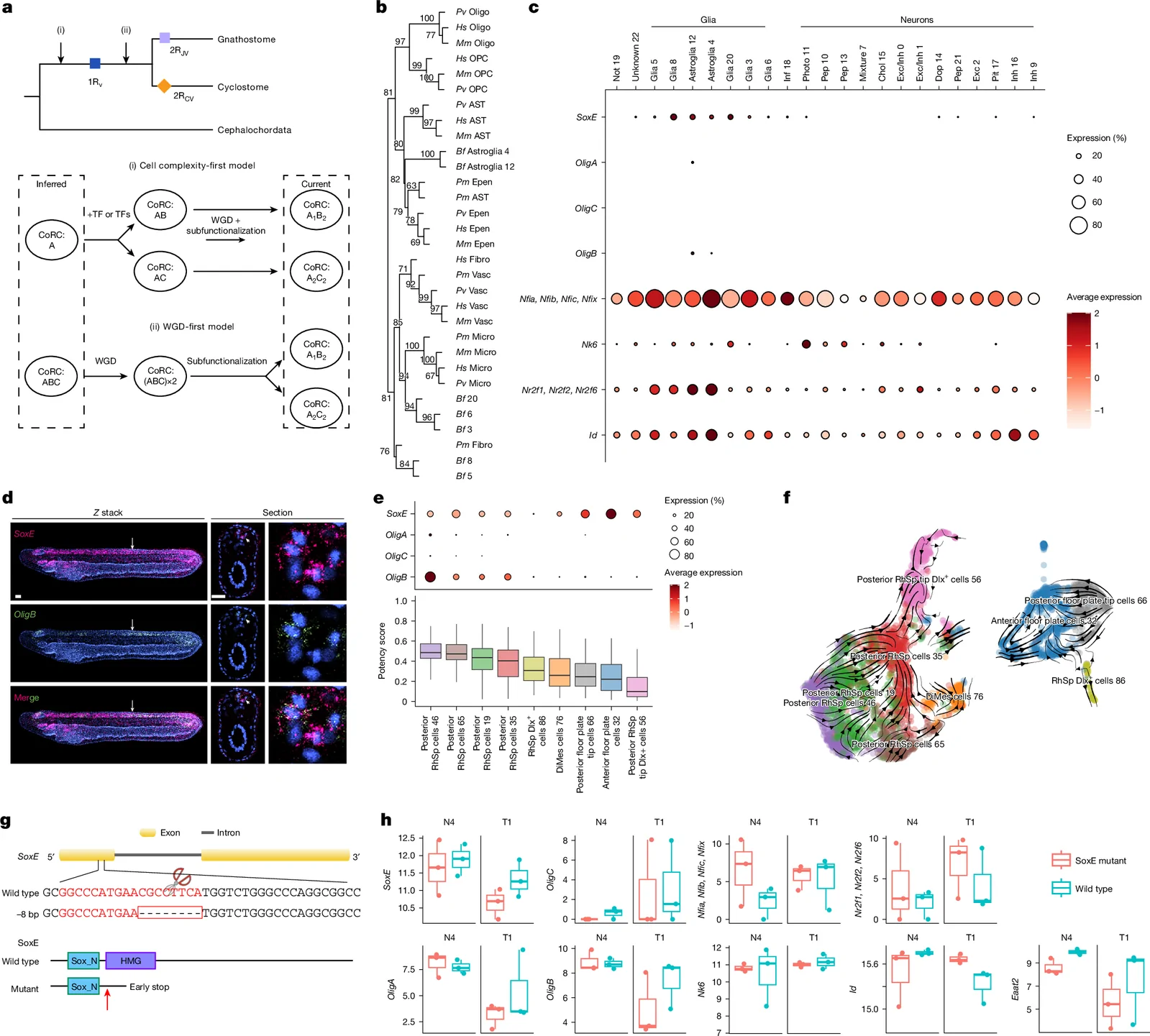
Macroglia evolution in chordates. **a**, Evolutionary models for inferring the WGD relationship to sister cell-type evolution. Rectangular dashed boxes indicate inferred ancestral and current states. **b**, Dendrograms showing hierarchical clustering of non-neuronal cell types from five chordates (human (*Hs*), mouse (*Mm*), lizard (*Pv*), lamprey (*Pm*) and amphioxus (*Bf*)) based on TF gene expression profiles (Methods). Bootstrap support (*n* = 1,000) numbers are indicated. **c**, Dot plots showing the expression of key TFs (*y* axis) between astrocytes and oligodendrocyte lineages (*x* axis) in adult amphioxus brain clusters. The dot size indicates the percentage of expressing cells, whereas the colour represents the scaled average expression. Full amphioxus cluster names are provided in Supplementary Table 2. **d**, Hybridization chain reaction and fluorescence in situ hybridization (HCR–FISH) analysis of *SoxE* (pink) and *OligB* (green) expression at stage T1 in amphioxus. Lateral views are shown on the left (anterior to the left, dorsal to the top), with merged channels indicated. Nuclei were labelled with DAPI (blue). Cross-sections at the level indicated by the arrow are shown. The arrowhead in the cross-section indicates the area enlarged on the right. Complete HCR–FISH datasets are provided in Extended Data Fig. 6f; experiments were repeated twice, with at least 15 embryos analysed per stage per replicate. Scale bar, 20 μm. **e**, Multipotency score of amphioxus CNS glia at T1. Cell number per cluster (left to right): 635, 396, 1,062, 757, 78, 226, 378, 771 and 526. Boxplot elements are as described in Fig. 2a. Colours represent cell types as in **f**. A corresponding *SoxE* and *Olig* gene dot plot is shown above. Embryonic glial clusters were annotated on the basis of markers and UMAP locations. RhSP, rhombencephalo-spinal primordium. **f**, RNA velocity for glial cells from amphioxus CNS at T1. **g**, Schematic of *SoxE* gene models and deletion, and domains of protein models of SoxE and SoxE mutants. **h**, Box plots showing log_2_ DESeq2-normalized expression levels for relevant TFs and the glia marker *Eaat2* in a SoxE homozygous mutant (*n* = 3) and wild type (*n* = 3) embryos. Box plot elements are as described in Fig. 2a. Each dot is an individual embryo genotyped by PCR and gel electrophoresis and by RNA-seq. Two-sided Wilcoxon rank-sum test, not significant for all comparisons.

The gap between correlation and causation in linking gene duplication to cell-type evolution reflects a broader challenge of connecting WGD to phenotypic complexity in early vertebrate evolution. We considered two alternative evolutionary models to evaluate causation: the cell complexity-first model and the WGD-first model (Fig. 3a). WGD postdating the emergence of novel cell types disproves a mechanistic or causative role. In the cell complexity-first model, sister cell types duplicated and became individualized by acquiring new regulators (neofunctionalization) alongside the ancestral CoRC. WGD then occurred to generate ohnologues of the CoRC members and to enable differential deployment in the cell types via passive processes. In the WGD-first model, the ancient CoRC already possessed the conserved TFs used in the individuation of two sister cell types. Following WGD, dosage selection and subfunctionalization separated CoRC ohnologues into different cell types, which drove their individuation. Given the complexity of the brain, it is possible that both routes occurred, but we aimed to determine which pathway was dominant. Resolving between models rested on how key conserved TFs that separate sister cell types are used in the outgroup. In general, we found that these were broadly expressed in amphioxus, a result consistent with the WGD-first model.

Cross-chordate mapping and regulon analyses revealed a clearer distinction among glial than neuronal families (Supplementary Fig. 8). Therefore, we focused on macroglia for a more detailed examination. The cross-chordate glia tree showed astrocytes clustering first with ependymal cells then with oligodendrocytes, with amphioxus cluster astroglia 4/12 grouping in ependymo-astrocytes (Fig. 3b and Methods). Relatedness was also supported by SAMap mappings (Extended Data Fig. 2c). We therefore considered that ependymal cells and astrocytes constitute a vertebrate sister cell-type family, whereas ependymo-astrocytes and oligodendrocytes are a gnathostome sister cell-type family. We identified 18 conserved TF orthogroups with paralogues differentially expressed between astrocytes and oligodendrocyte lineages, and 6 between astrocytes and ependymal cells (Methods and Supplementary Table 4). We report details for the former group here (for the latter, see Supplementary Fig. 9). Several of these TFs are terminal selectors that separated the sister cell types. For example, in the *SoxE* ohnologue family (which includes *Sox8*,* Sox9* and* Sox10*), Sox9 activates Nfia and Nfib, which together drive radial glia to astrocyte fate^32,33^, Sox10 governs oligodendrocyte differentiation, and Sox8 acts transiently earlier^34^. Olig2 specifies oligodendrocyte precursor cells, whereas Olig1 and Nkx6-2 promote oligodendrocyte differentiation^32,35^. Radial glia are progenitors for vertebrate macroglia, whereby some radial glia, astrocytes and oligodendrocyte lineages co-express *SoxE* and *Olig* in vertebrates^32,36^.

**Extended Data Fig. 6:**
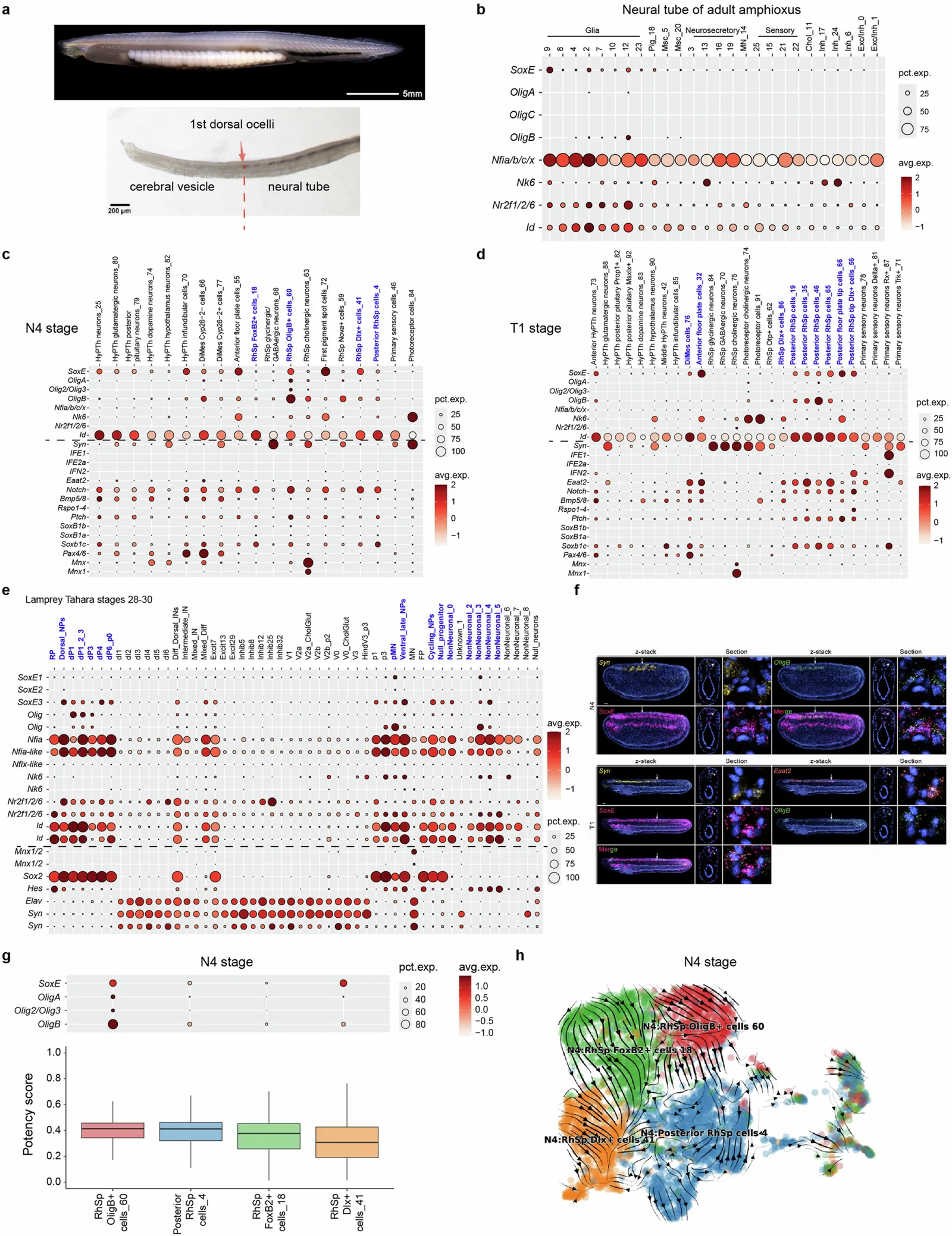
co-expression of key TFs between astrocytes and oligodendrocytes. **a**, Left lateral view of a live adult amphioxus (*Branchiostoma floridae*) under incident light (top), and schematic of the neural tube dissection separating the brain (cerebral vesicle) from the remaining neural tube (bottom). The dissection site corresponds approximately to the region around the first dorsal ocellus. **b**, Dot plots showing expression of key TFs between astrocytes and oligodendrocyte lineages in adult amphioxus neural tube. Cell clusters were annotated based on top marker genes (data not shown). Dot size indicates the percentage of cells expressing each gene within a given cluster; colour represents scaled average expression. **c**,**d**, Dot plots showing expression of key TFs between astrocyte and oligodendrocyte lineages in the amphioxus central nervous system at the N4 (**c**) and T1 (**d**) embryonic stages. Dot size and colour are as in (**b**). Glial clusters are highlighted in bold blue, defined based on glial marker gene expression below the dotted lines and confirmed by the relatedness on UMAP at each stage (data not shown). **e**, Dot plots showing key TFs between astrocyte and oligodendrocyte lineages in lamprey Tahara stages 28-30. Dot size and colour are as in (**b**). Glial clusters are highlighted in bold blue, defined based on glial marker gene expression below the dotted lines. **f**, HCR-FISH analysis of *Syn* (yellow), *SoxE* (pink), *OligB* (green), and *Eaat2* (coral orange) expression at the N4 and T1 stages. *EAAT2* shows no expression at N4 stage, therefore no ISH is shown. Lateral views are shown on the left (anterior to the left, dorsal to the top), with merged channels indicated. Nuclei were labelled with DAPI (blue). Corresponding cross-sections at the level indicated by the white arrow are also shown. The white arrow in the cross-section indicates the area enlarged on the right. **g**, Multipotency score of glia cells from amphioxus CNS at N4 stage. Number of cells per cluster (left to right: 641, 1547, 1207, 819). Boxplot elements as Fig. 2a. Colours in multipotency score represent different cell type as shown in (**h**). A corresponding *SoxE* and *Olig* genes dot plot is shown at upper panel for convenience. **h**, RNA velocity for glial cells from amphioxus CNS at N4 stage.

Amphioxus glia clusters co-expressed these conserved CoRC genes (Fig. 3c), but only cluster astroglia 12 had a few cells (*n* = 4) that colocalized *SoxE* and *OligB* (the most highly expressed *Olig* gene; Fisher’s exact test, *P* = 0.016, odds ratio (OR) = 4.5). Given this low number, and the debated boundary between brain and spinal cord homologous regions in amphioxus^37^, we generated additional data for the rest of the adult neural tube (19,140 cells; Extended Data Fig. 6a). This analysis showed much stronger colocalization between *SoxE* and *OligB* (Fisher’s exact test, *P* < 2.2 × 10^–16^, OR = 5.1; Extended Data Fig. 6b). We investigated single-cell data from amphioxus embryos^29^ and lamprey embryonic neural tubes, and confirmed that there was extensive colocalization of *SoxE* and *Olig* in glial cells in both species (Extended Data Fig. 6c–e). We experimentally confirmed co-expression (Fig. 3d, Methods and Extended Data Fig. 6f) and identified a directional trajectory from glial clusters with higher *Olig* expression towards those with lower expression, a result that matched the multipotency analysis (Fig. 3e,f, Methods and Extended Data Fig. 6g,h). These data suggest that amphioxus possesses equivalents to radial glia, which may act as progenitors and produce astroglia.

We generated an amphioxus *SoxE* mutant line to assess its role in glial development (Fig. 3g and Methods). In contrast to the severe phenotypes observed after mutations in *Sox9* and* Sox10* in vertebrates^38,39^, amphioxus *SoxE* mutations did not lead to obvious morphological defects during embryonic stages. However, they did exhibit delayed development, abnormal development of gill slits in larvae and reduced survival. Nonetheless, transcriptomic comparisons between wild-type and *SoxE*^−/−^ embryos at the N4 and T1 developmental stages revealed that glial markers (including key TFs) co-expressed with *SoxE* were downregulated in mutants (Fig. 3h). This finding indicates that *SoxE* is important for amphioxus glial differentiation.

These data identify amphioxus glia that co-express TFs separating sister cell types of vertebrate macroglia. We infer that this is the ancestral state for macroglia and that these cells are amphioxus equivalents of radial glia. In vertebrates, ohnologues of these TFs separate macroglial subtypes.

## Subfunctionalization and neofunctionalization

**Fig. 4:**
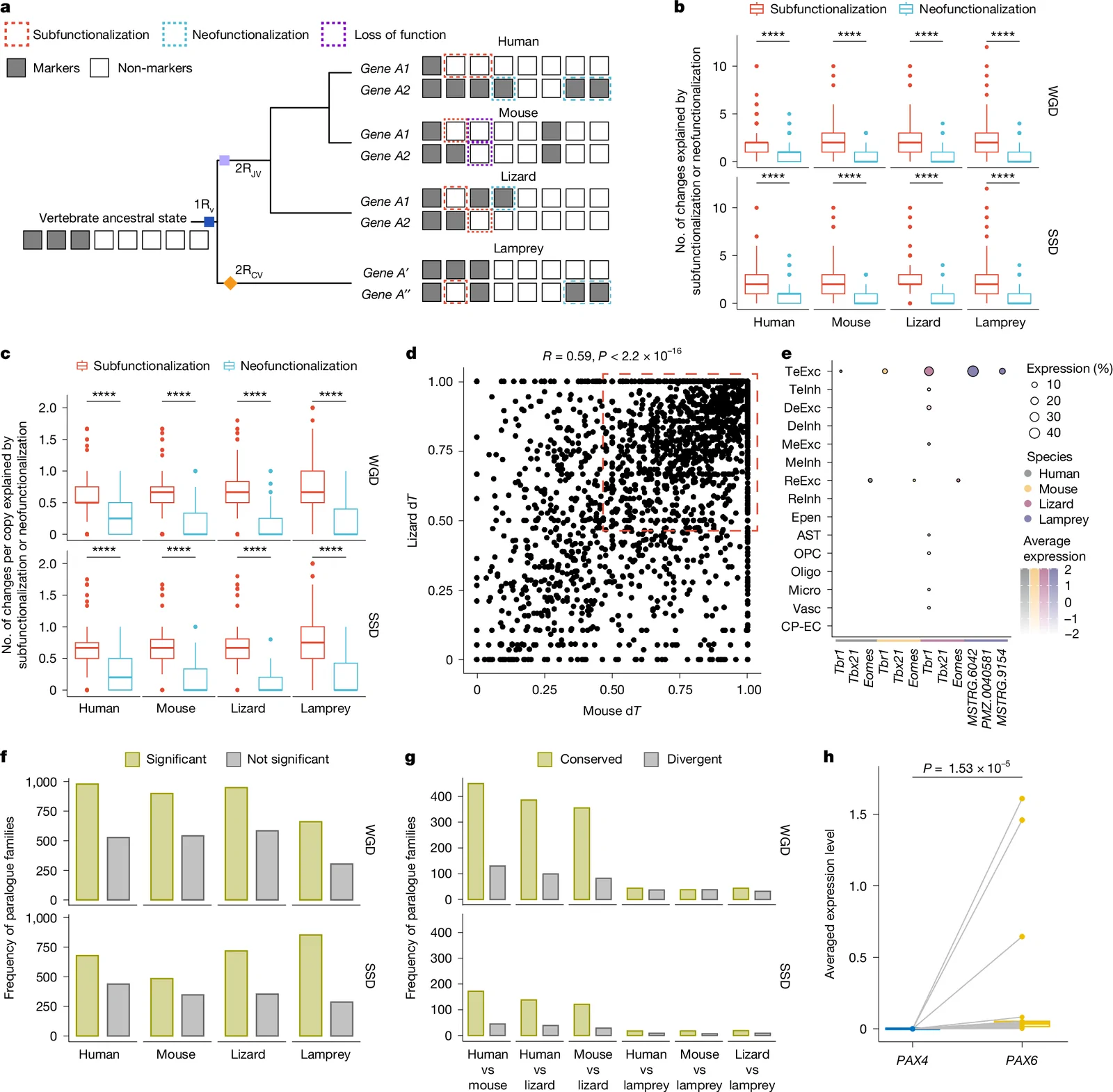
Divergence of paralogues is associated with cell-type evolution. **a**, Ancestral state inference showing subfunctionalization (splitting ancestral roles; red), neofunctionalization (gain of new roles; blue) and loss of function (purple). Each column of boxes represents a cell-type family. For example, in the third column (a cell-type family), human, lizard and lamprey (three out of four) have at least one gene used as a marker. Therefore, the ancestral state is a marker. **b**, The number of changes explained by subfunctionalization or neofunctionalization for each WGD and SSD paralogue family. Statistical comparison by two-sided paired Wilcoxon signed-rank test: ****P < 2.22 × 10^–16^ for all tests. Number of paralogue families: WGD = 1,872, SSD = 1,050. Box plot elements are as described in Fig. 2a. **c**, The number of changes per copy explained by subfunctionalization or neofunctionalization for each WGD and SSD paralogue family. *P* value estimated and number of paralogue families are as in **b**. Box plot elements are as in Fig. 2a. **d**, Expression divergence of orthogroups for mouse and lizard, estimated using Pearson’s correlation. A d*T* of 1 means copies were not expressed in the same cell-type family. The red dotted box highlights paralogue families with high expression divergence for both species. **e**, Expression pattern of the *Tbr1* subfamily. Legend details are as in Fig. 1c. **f**, The number of WGD and SSD paralogue families (with only two copies) that have and do not have a significantly dominant copy. **g**, The number of WGD and SSD paralogue families using the same genes or not as the dominant copy in species pairwise comparisons. **h**, Pseudobulk expression of *PAX6* and* PAX4* family for human. Each dot represents a cell-type family, and the grey line between genes connects the same cell-type families. Both Friedman tests and two-sided paired Wilcoxon signed-rank tests showed significance for all species, but only Wilcoxon results are shown.

**Extended Data Fig. 7:**
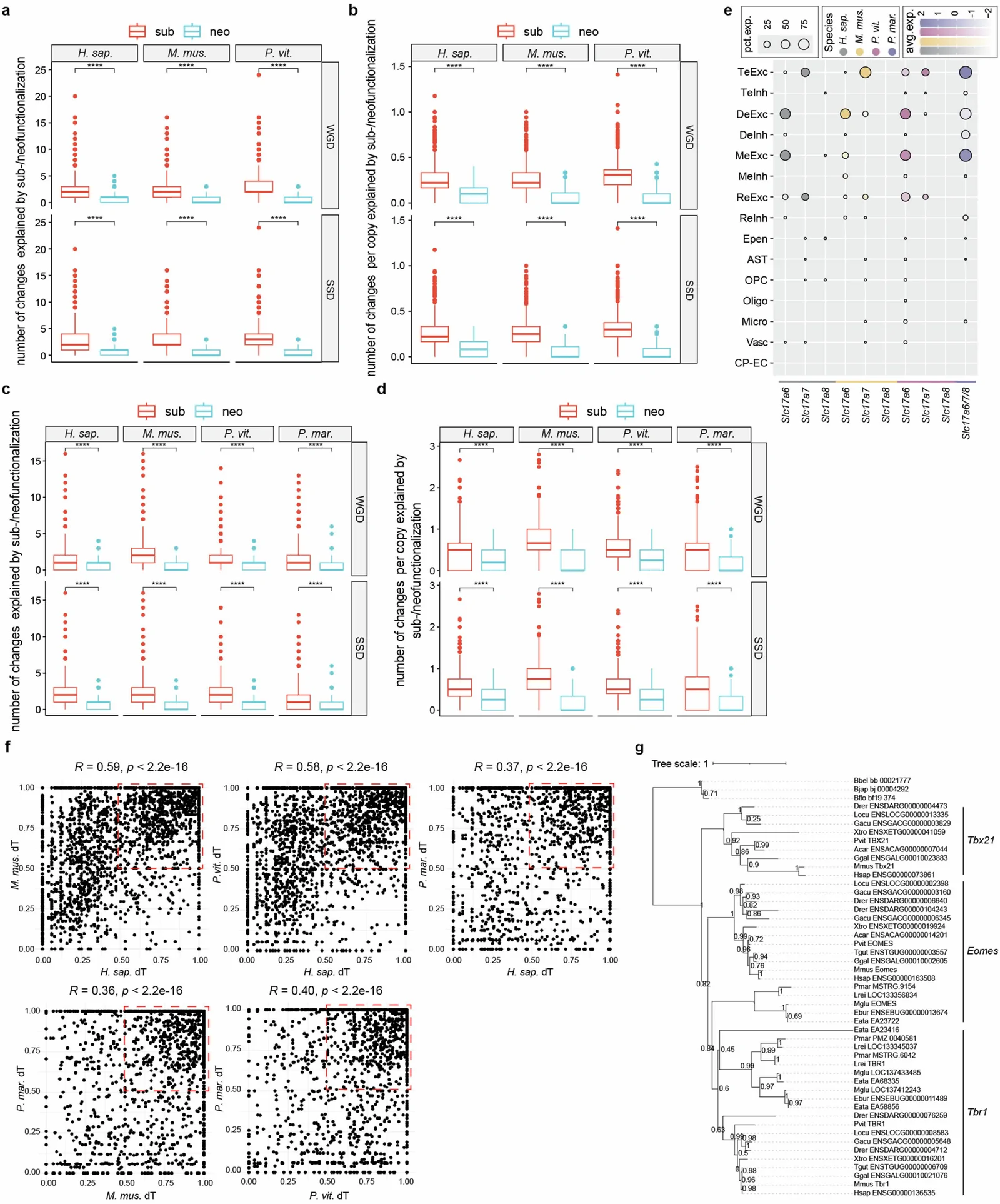
Expression shifts of paralogues. **a**,**b**, The number of changes (**a**) and changes per copy (**b**) explained by subfunctionalization (sub) and neofunctionalization (neo) for each ohnologue/SSD paralogue orthogroups in amniotes. Number of paralogue family (WGD = 1872, SSD = 1050). Boxplot elements as Fig. 2a. Ancestral state was predicted based on three amniotes. Statistical significance was accessed by paired Wilcoxon signed-rank test. ns (not significant); * (*P* < 0.05); ** (*P* < 0.01); *** (*P* < 0.001); **** (*P* < 0.0001). **c**,**d**, The number of changes (**c**) and changes per copy (**d**) explained by subfunctionalization and neofunctionalization for each ohnologue/SSD paralogue family. Number of paralogue families analysed and boxplot elements are the same as (**a**). Binarized ancestral expression states were predicted based on Trinarization scores across vertebrates. Some outliers are shared between the WGD and SSD panels in (**a**–**d**) because those orthogroups were assigned to both duplication categories during classification. Statical comparison (**a**,**d**) by two-sided paired Wilcoxon signed-rank test: **** (all *P* < 2.22e-16). **e**, Dot plot showing the expression of *Slc17a6/7/8* gene family. The size of dot represents the percentages of cells within each cluster expressing that gene. The gradient colours from white to species colour were scaled for each gene on individual species. Species of the gene is also shown as coloured line at the bottom of the plot. **f**, The average expression divergence (dT) of orthogroups compared in a pairwise manner, estimated using Pearson correlation. dT = 1 represents copies were not expressed in the same domain at all while dT=0 means all copies were expressed in the same domain. The correlation was calculated by two-sided Pearson with statical significance in all pairwise comparisons. The red dotted box highlights paralogous orthogroups exhibiting high expression divergence in both species. The amount of expression shifts toward mouse and lizard when compared with human, likely reflect methodological differences between scRNA (mouse and lizard) and snRNA (human), where scRNA offers greater depth and snRNA-seq provides broader coverage. **g**, A rooted phylogenetic tree of the *Tbr1* subfamily of T-box genes, generated by OrthoFinder. The tree scale represents a unit of evolutionary distance between gene sequences within an orthogroup. Three main genes in the *Tbr1* subfamily were highlighted on the right of the tree. Lamprey MSTRG.9152 and related genes belong to *Eomes* rather than *Tbr1* subfamily based on OrthoFinder resolved gene trees (considering species tree structure) (data not shown). Bootstrap values are indicated at the internal nodes.

Paralogous genes could be expressed by different cell types through subfunctionalization or neofunctionalization. We sought to determine which mechanism plays the predominant part for WGD and SSD paralogues. Expression was binarized as ‘marker’ or ‘not marker’, and the smallest groups of cell types using at least one paralogue as a marker in at least three out of four vertebrates were considered as the ancestral state for vertebrates (Fig. 4a and Methods). We classified ohnologue, SSD paralogue and paralogue orthogroups if at least three out of four species contained a pair of the corresponding paralogues. This analysis produced 1,872 ohnologue, 1,050 SSD paralogue and 2,693 paralogue orthogroups, with 339 overlapping between ohnologue and SSD paralogue orthogroups. We then calculated the change in each paralogue orthogroup compared with the inferred ancestral state for each species (Methods). In paralogue orthogroups, approximately 78%, 21% and <1% of total changes in marker usage could be attributed to subfunctionalization, neofunctionalization and loss of function (loss by all copies of being a marker in that cell type), respectively. Across paralogue, SSD paralogue and ohnologue orthogroups, these proportions remained relatively consistent, with the number of changes and number of changes per gene explained by subfunctionalization consistently (and significantly) higher than those explained by neofunctionalization for all species and duplication types (Fig. 4b,c). Similar patterns were found when analysing amniotes only (Methods and Extended Data Fig. 7a,b). We also performed the above approaches on expression matrices binarized on the basis of the Trinarization score^3^, which displayed similar trends (Extended Data Fig. 7c–e). This finding indicates that the use of paralogues to increasingly specialize cell types in vertebrate evolution was mainly shaped by subfunctionalization. These results provide the strongest support for the duplication, degeneration and complementation (DDC) model^9^.

We next asked whether gene expression in paralogue orthogroups shifts to similar degrees across species. We defined the expression domain on the basis of the Trinarization score^3^ at the homologous cell-type family level then calculated the average expression divergence (d*T*) among paralogues in each orthogroup for each species (Methods). Pairwise comparisons revealed that most paralogues extensively diverged in both species and some exhibited shifts mainly in one species (Fig. 4d and Extended Data Fig. 7f). For example, the *Tbr1* subfamily of T-box genes (d*T* = 1 for human, mouse and lizard, and d*T* = 0.67 for lamprey) duplicated through WGDs at the base of vertebrates, which gave rise to *Tbr1*, *Eomes* and *Tbx21* in jawed vertebrates (Extended Data Fig. 7g and confirmed elsewhere^1^). In our dataset, *Tbr1* was exclusively expressed in glutamatergic neurons of the telencephalon, whereas *Eomes* was expressed in rhombencephalon glutamatergic neurons in amniotes (Fig. 4e).

## Dosage selection across cell types

**Extended Data Fig. 8:**
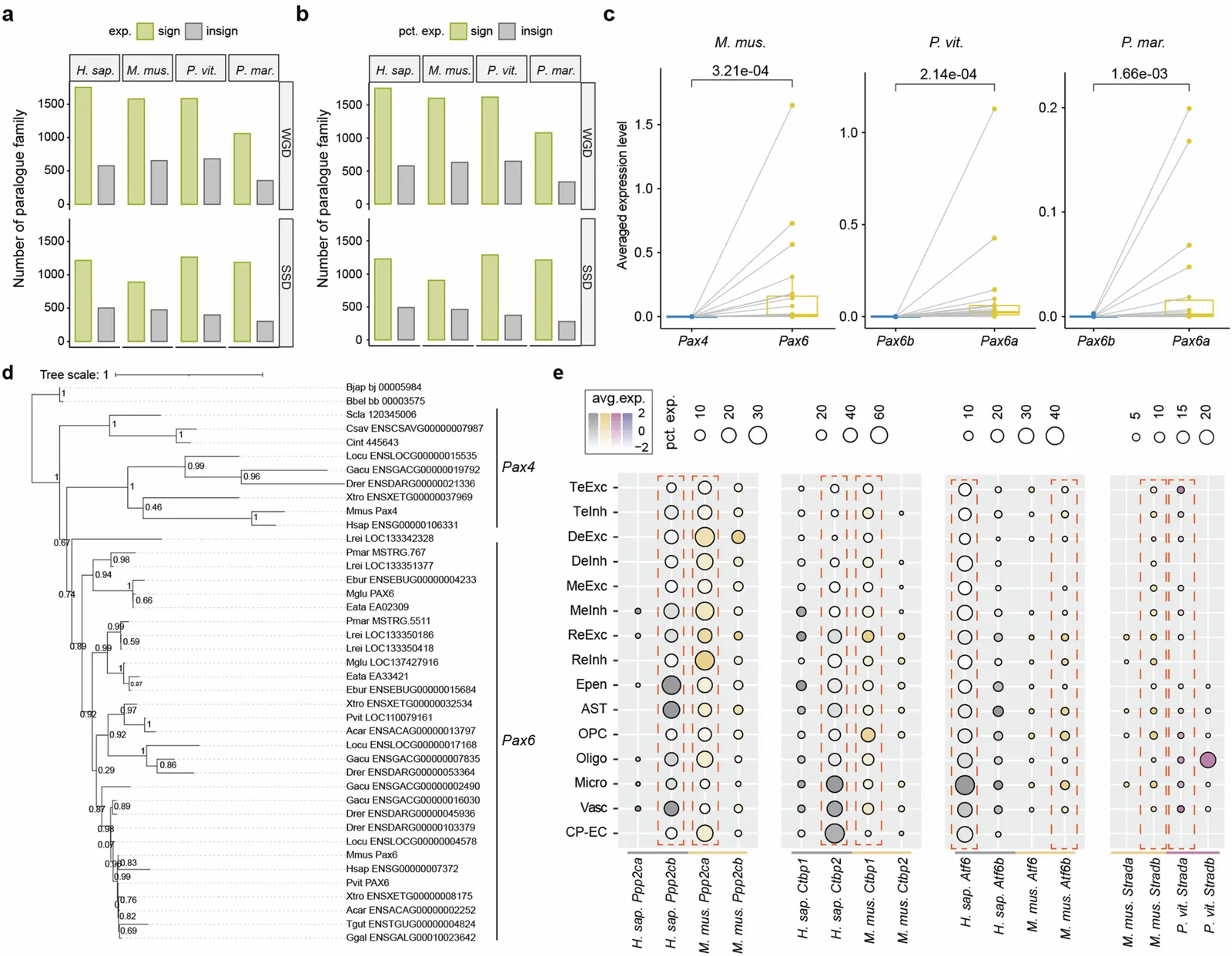
Pan-expression differences within paralogue families. **a**,**b**. The number of ohnologue/SSD paralogue families where the expression of at least one copy significantly differs from other copies. Ohnologue families and SSD paralogue families were labelled as WGD and SSD, respectively. (**a**) significance was evaluated at expression level with two-sided Friedman test. (**b**) significance was evaluated at percentage of cells expressing that gene (pct. exp.) level with two-sided Friedman test. ‘sign’ means p-value < 0.01; ‘insign’ means p-value >= 0.01. **c**, Pseudobulk expression of *Pax6-Pax4* family for mouse, lizard, and lamprey, respectively. Pseudobulk expression was calculated by the average of SAM normalised expression. Each dot represents a cell-type family and the grey line between paralogues denotes the comparison of genes at the same cell-type family. Significance was evaluated with two-sided paired Wilcoxon signed-rank test. Number of cell-type families analysed and boxplot elements are the same as Fig. 2a. **d**, A rooted phylogenetic tree of the *Pax4/6* gene family, generated by OrthoFinder. The tree scale represents a unit of evolutionary distance between gene sequences within an orthogroup. Two main categories of the *Pax4/6* were highlighted on the right of the tree. Bootstrap values are indicated at the internal nodes. **e**, Dot plots showing some cases of paralogues that are used differently by different species. The size of dot represents the percentages of cells within each cluster expressing that gene. The gradient colours from white to species colour were scaled for each gene on individual species. Species identity is also shown as coloured line at the bottom of the plot. The dotted orange boxes label the dominant copy.

Studies using bulk transcriptomes revealed gene expression changes following gene duplication^40,41^. This result could be explained by the gene balance hypothesis, which proposes that high expression of duplicated genes can be selectively disadvantageous owing to stoichiometry imbalance^42,43^. We tested whether this hypothesis extended to the cell-type level to potentially contribute to the route to subfunctionalization. Most (>65%) WGD and SSD paralogue families contained at least one copy that significantly differed from others with respect to the expression level or percentage of expressing cells (Friedman test, *P* < 0.01; Extended Data Fig. 8a,b). We limited the analysis to paralogue families with two copies and counted those with a significantly dominant copy (as assessed by expression level and percentage of expressing cells). Overall, 58–76% of these paralogue families, whether derived by WGD or SSD, had a significantly dominant copy in a pervasive way and not specific to cell types (Fig. 4f and Methods). We also found that ohnologues were likely to have more protein–protein interactions and that ohnologue TFs regulate significantly higher numbers of targets than SSD paralogue TFs. Ohnologous TFs regulated more similar targets compared with SSD paralogous TFs (Supplementary Text and Supplementary Figs. 18–20), and there were stronger evolutionary constraints on ohnologous coding sequences.

We then asked whether different species use the same gene as the dominant copy in each gene family. Human and mouse had the highest similarity compared to other species pairs (Fig. 4g), a result that reflected their phylogenetic relationship. The number of ohnologue families sharing a dominant copy was higher than SSD families across all species comparisons (Fig. 4g). For example, *PAX6* was highly expressed in astrocytes, rhombencephalon glutamatergic neurons and several other cell types, whereas *PAX4* showed limited expression (Fig. 4h and Extended Data Fig. 8c,d). This general trend can be explained by either stronger dosage selection on ohnologues and/or if SSDs emerged in a specific lineage and are not shared by the species analysed. The high degree of conservation of dominant copies across vertebrates suggests that dosage selection occurred soon after duplication (especially WGD) irrespective of the cell type and before divergence of lineages studied. This could be a result of selection following an immediate transcriptional response after genome duplication^9,40,43^. Consequently, genes can be retained sufficiently long before subfunctionalization and/or neofunctionalization^9,44^. Conversely, many paralogues (for example, *Ppp2ca*, *Ppp2cb*, *Ctbp1*, *Ctbp2*, *Atfb6*, *Atfb6b*, *Strada* and *Stradb*) were used differently by different species (Extended Data Fig. 8e).

## Genome duplication and regional identity

**Extended Data Fig. 9:**
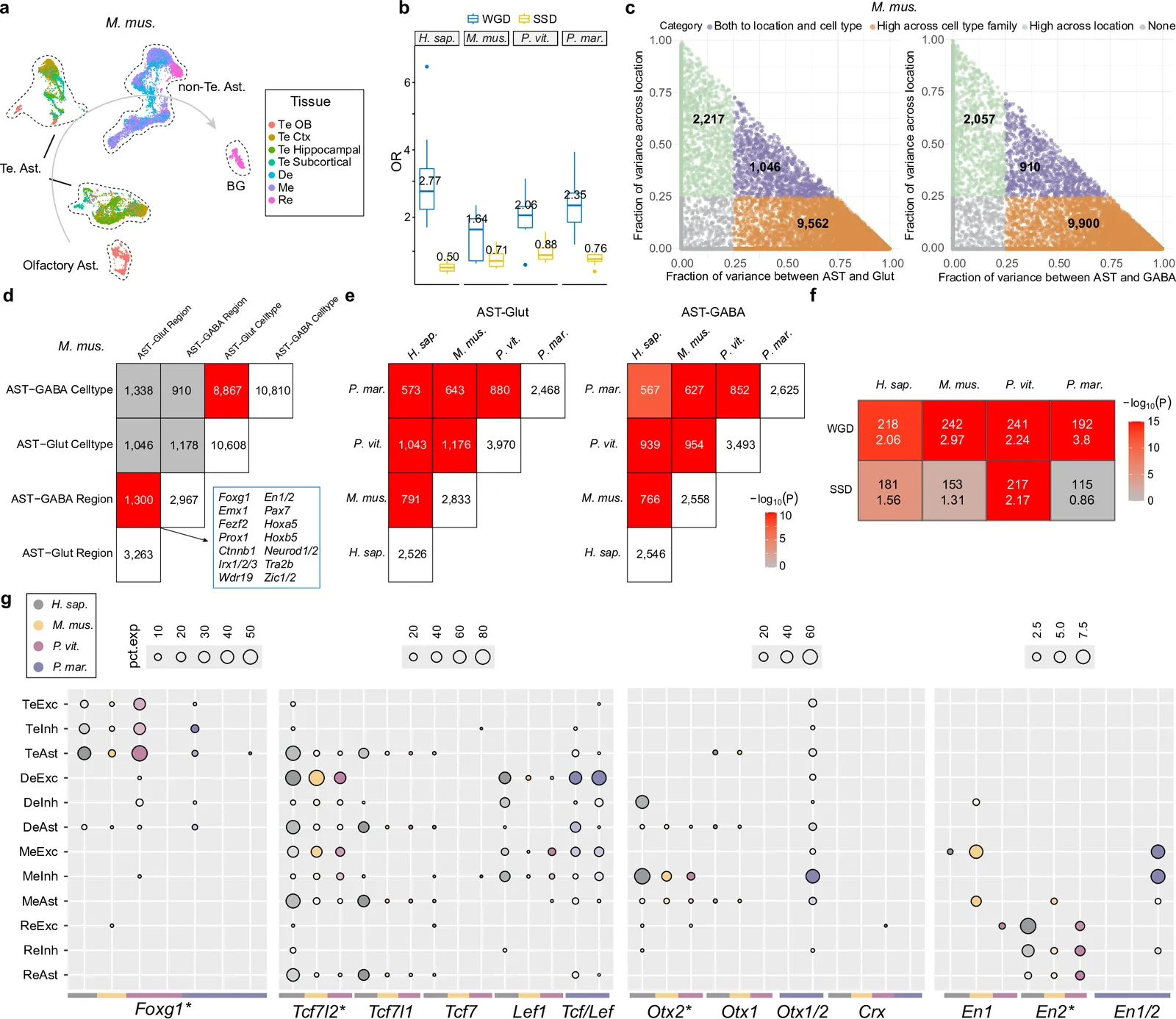
Evolution of regional programmes contributing to cell diversity. **a**, UMAP showing projection of mouse astrocytes with anterior to posterior location indicated. BG: Bergmann glia; Te: Telencephalon. **b**, OR calculated from two-sided Fisher’s exact test between WGD/SSD paralogues and DEGs of astrocyte subtypes. Each dot represents a subtype of astrocytes. Number of astrocyte subtypes analysed per species (human, 10; mouse, 9; lizard, 10; lamprey, 10). Boxplot elements as Fig. 2a. **c**, Variance decomposition of transcriptome estimates the relative contribution of cell-type family and brain divisions (Te/De/Me/Re) to variance in mouse gene expression. Green dots indicate genes with >25% contribution to location, orange with >25% contribution to cell type, purple with >25% to both, grey with <25% to both. Bold numbers are metagene numbers. **d**, Comparison matrix of gene sets identified in (**c**). The colour is red if two-sided Fishers’ exact test shows significance with OR > 1. Colour gradient represents -log_10_(*P*). The listed genes overlap between two sets of regional identity genes. **e**, The comparison matrix of AST-GABAergic neurons and AST-Glutamatergic neurons regional orthogroups across species. For each species, a regional orthogroup was defined as such if any regional identity genes of that species were in that orthogroup. Colour intensity is as in (**d)**. **f**, Comparison matrix showing relationships between paralogues (WGD and SSD paralogues) and genes in conserved regional orthogroups for each species, by Fisher’s exact test. Genes in conserved regional orthogroups were identified if regional genes in (**c**) in the vertebrate conserved regional orthogroup. The upper number for each comparison denotes the number of overlaps and the lower number the OR. **g**, Dot plots showing expression of selected genes (labelled with *) involved in regional identity and their ohnologues in neural families from different brain divisions. Dot size represents percentages of cells expressing that gene. Colour gradients from white to species colour were scaled for each gene on individual species. Species identity is shown at the bottom.

Cell types often require appropriate regional identity to achieve proper function^24^ (Supplementary Text). To explore whether WGD contributed to cell-type evolution through defining regional identity, we analysed macroglia subtypes (Supplementary Table 5 and Supplementary Fig. 10a,b). Astrocytes were the most diversified, with strong regional variance (Extended Data Fig. 9a and Supplementary Fig. 10c–e). Ohnologues were significantly associated with DEGs of astrocyte and oligodendrocyte subtypes (Extended Data Fig. 9b and Supplementary Fig. 10f–h), but not ependymal cells, in human and mouse, which may be due to low representation (Supplementary Fig. 10i,j).

**Extended Data Fig. 10:**
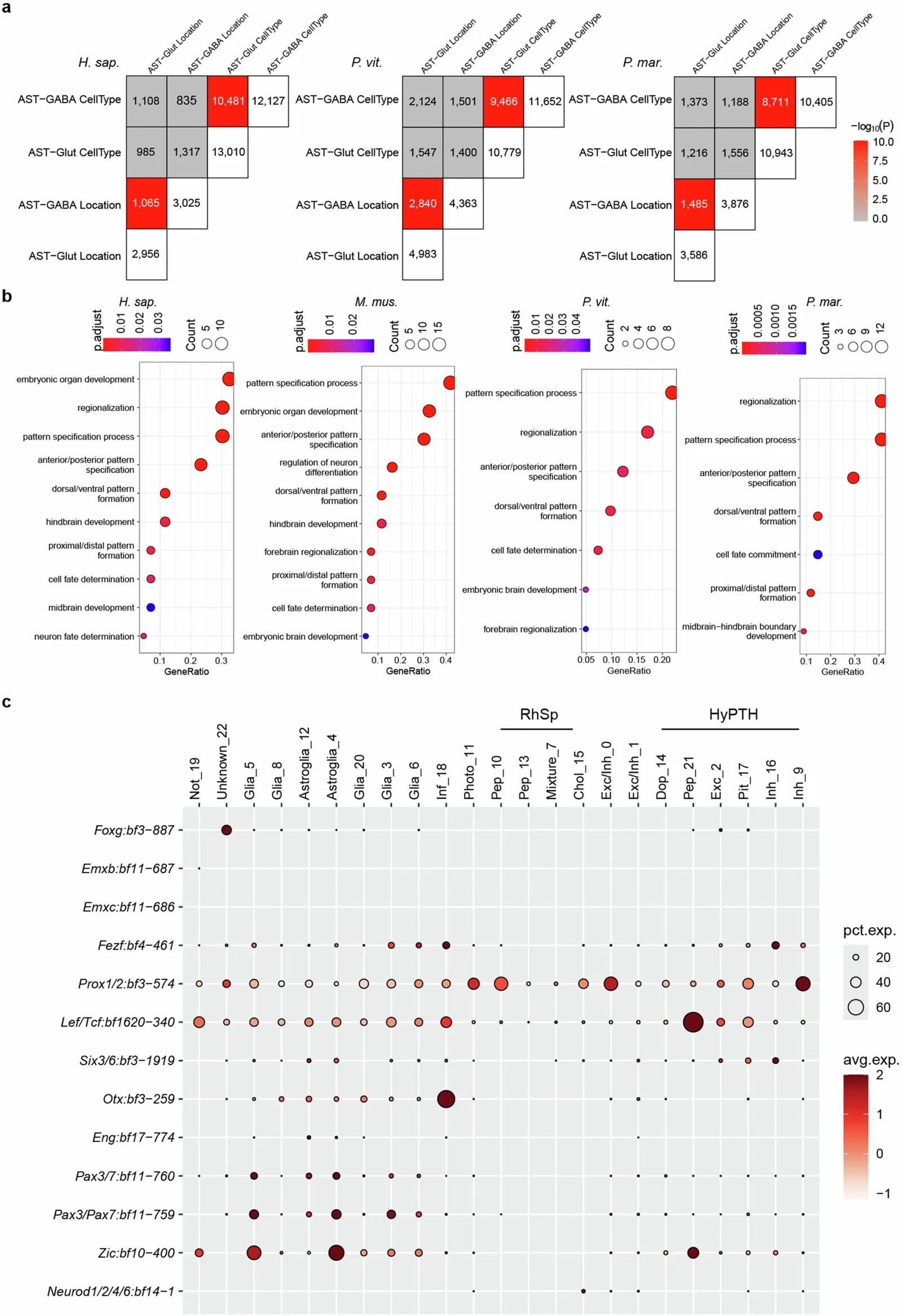
Comparison and enrichment of regional-identity genes. **a**, Heatmap showing the comparison matrix of potential regional-identity gene sets in Supp. Table 6. The colour is red if two-sided Fishers’ exact test shows significance with OR > 1. The gradient red colour represents -log_10_(p-value). **b**, Dot plots showing GO enrichment of genes in conserved regional orthogroups for each species. Genes in conserved regional orthogroups were identified if genes were highly contributing to regional identity and in the vertebrate conserved regional orthogroups. **c**, Dot plot showing vertebrate conserved regional TFs listed in Supp. Text, in amphioxus. Dot size represents the percentage of cells expressing that gene. Dot colour reflects the scaled average expression (−2 to 2) across all clusters. Dots representing clusters with <1% of cells expressing the gene were omitted to reduce noise.

We discovered genes that contributed to regional and cell-type identity (Extended Data Fig. 9c, Methods and Supplementary Table 6) and compared GABAergic neurons with astrocytes and compared glutamatergic neurons with astrocytes. Regional genes significantly overlapped across cell types (Extended Data Figs. 9d and 10a), and relevant orthogroups were significantly shared across species (Extended Data Fig. 9e). This result suggests that at least part of the regional programs were shared across neural cell-type families and conserved across vertebrates. These genes were significantly enriched in regionalization, brain development and cell-specification annotations (Extended Data Fig. 10b). Among these were several key genes implicated in brain development and cell specification (Supplementary Text). Notably, the average number of copies (2.5–3.4) in these regionalization orthogroups was significantly larger than the average size of orthogroups for each species (1.3–1.5, two-sided Wilcoxon rank-sum test, *P* < 2.2 × 10^–16^). Furthermore, genes in regionalization orthogroups were more strongly associated with ohnologues than SSD paralogues (Extended Data Fig. 9f). As observed above, these genes and their ohnologue pairs underwent significant expression shifts following WGDs (Extended Data Fig. 9g). A comparative analysis with amphioxus suggests that many vertebrate regional regulatory programs arose after WGD (for example, *Foxg1* for telencephalon), with limited conservation to amphioxus (Supplementary Text). Following WGDs, such TFs were preferentially retained and underwent expression shifts that are associated with cell-type family diversification across vertebrate brain divisions.

## Lasting effect of WGD on cell types

**Fig. 5:**
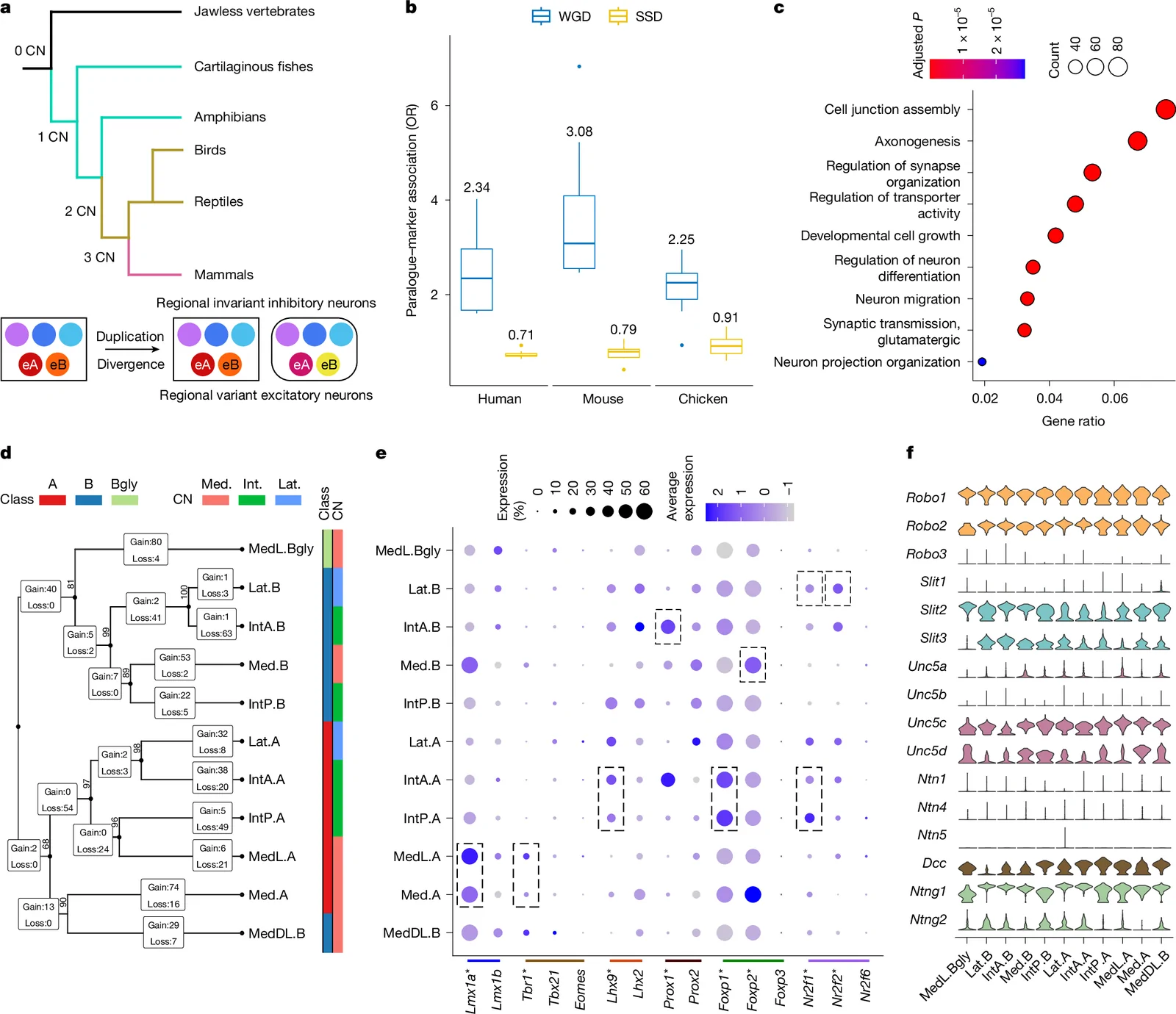
Ohnologues in CN evolution. **a**, Illustration showing CN pairs in vertebrates and the duplication and divergence of excitatory neurons in them. **b**, ORs calculated from two-sided Fisher’s exact test on WGD and SSD paralogues with cell-type DEGs. The number of CN excitatory neuron subtypes analysed: human, 8; mouse, 11; and chicken, 8. Boxplot elements are as in Fig. 2a. **c**, GO enrichment of DEGs of excitatory neurons in the CN of mice by clusterProfiler. The colour represents the adjusted *P* value and the dot size denotes the gene ratio. **d**, Dendrogram of excitatory neurons in the medial (Med.), interposed (Int.) and lateral (Lat.) CN and the number of predicted genes with gain of function and loss of function (regarding expression domain). The left colour bar represents cell-type classes (A, B and Bgly) and the right bar represents the location of corresponding CN. Bgly is a small cluster in the excitatory neuron clusters that expresses *Slc6a5* but not *Slc17a6*. The number near the node is approximately unbiased (AU) *P* values, computed by pvclust with 1,000 times multiscale bootstrap resampling. Rectangles in the branch contain the number of genes gained and lost in expression domain. The number following the semicolon represents the number of TF genes involved. **e**, Expression pattern of important TF genes (labelled with an asterisk) and their ohnologues. Dashed boxes highlight cross-species-conserved TF marker genes. The colour represents the scaled average expression for each gene. The dot size represents the percentage of cells expressing that gene in a certain cell type (percentages above 60% were capped at 60%). **f**, Violin plot of genes involved in the axon guidance system. Genes from the same orthogroup are the same colour. IntA, anterior interposed CN; IntP, posterior interposed CN; MedDL, medial dorsolateral CN; MedL, medial lateral CN. Schematic in **a** adapted with permission from ref. ^46^, AAAS.

**Extended Data Fig. 11:**
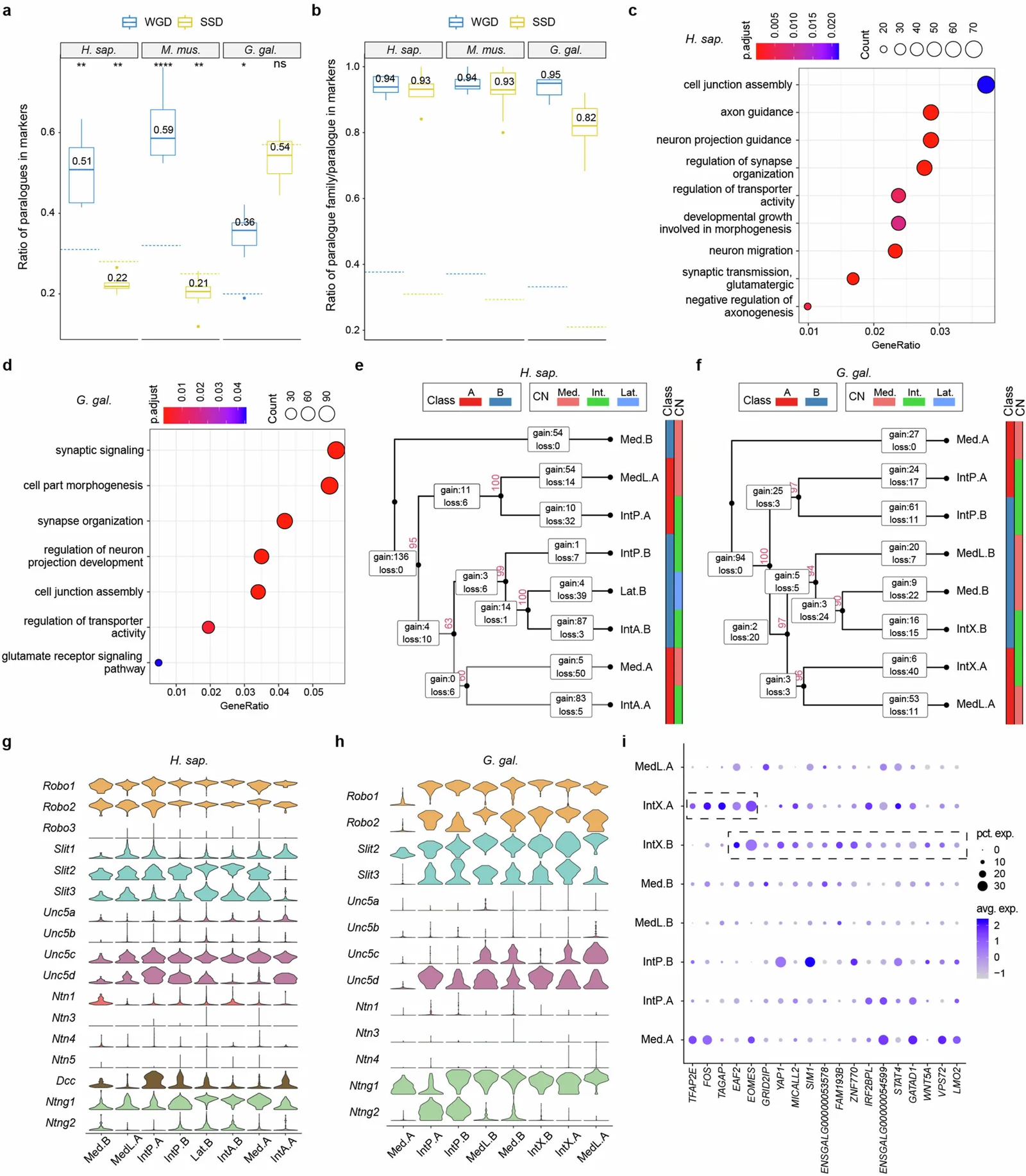
Excitatory neurons in CN and genes important in subtype differences. **a**, Proportion of ohnologues (WGD, blue) and SSD paralogues (SSD, yellow) among DEGs in the subtype of CN excitatory neurons. Background proportions of expressed protein-coding genes are indicated by blue and yellow dotted lines. Significance was evaluated by one-sample Wilcoxon signed-rank test (two-sided). ns (not significant); * (*P* < 0.05); ** (*P* < 0.01); *** (*P* < 0.001); **** (*P* < 0.0001). Number of CN excitatory neuron subtypes analysed (human, 8; mouse, 11; chicken, 8). Boxplot elements as Fig. 2a. **b**, The ratio of the number of WGD families (blue) and SSD families (yellow) that include markers to the number of paralogues that are markers for each cell type. Cell types analysed and boxplot elements are the same as (**a**). If only one copy in each gene family were used as a marker, the ratio would be 1. The number shown for each box represents the median value. The background ratio denotes the number of paralogue families to number of paralogues and were represented by a blue (WGD) and a yellow (SSD) dotted line to be used as background for each species. The *p*-value is displayed as in (**a**). **c**,**d**, GO enrichment of DEGs of CN excitatory neurons for human (**c**) and chicken (**d**). **e**,**f**, Dendrogram of excitatory neurons in CN and number of predicted TF genes with gain- and loss-of-function (expression domain) for human (**e**) and chicken (**f**). The left colour bar represents cell-type class and right bar represents location of CN. The red number near the node is Approximately Unbiased (AU) p-values, computed by pvclust with 100 times of multiscale bootstrap resampling. Rectangles in the branch contain the number of genes gain and loss in particular expression domain (CN or ancestral CN). The number following semicolon represents the number of TFs involved. **g**,**h**, Violin plots showing genes in axon guidance system are differentially expressed across CN in human (**g**) and chicken (**h**). **i**, Dot plot showing potentially important TFs in the evolution of IntX. The dotted boxes indicate up-regulated TFs for IntX.A and IntX.B compared to other subtypes.

To test whether our findings hold true in relation to cell types that definitively emerged long (at least 150 million years) after WGD^1,45^, we analysed cerebellar nucleus (CN) scRNA atlases^46^. During vertebrate evolution, an archetypal CN, including its conserved combination of cell subtypes, duplicated^46^ (Fig. 5a). Lamprey lacks a CN, cartilaginous fishes and amphibians have one CN pair, reptiles and birds have two pairs, and mammals have three pairs^47,48^. We focused on excitatory neurons in the CN as they are regionally variant and largely confined to defined subnuclei^46,49^. We performed the same analyses as above and found the same relationships between ohnologues, SSD paralogues and DEGs (Fig. 5b and Extended Data Fig. 11a,b). Consistently, 13 out of 20 experimentally validated markers of CN excitatory neurons^46^ were ohnologues (Supplementary Table 7). DEGs were enriched in axonogenesis, axon guidance, migration and synaptic organization, which reflected their functional diversity across subnuclei and between neuron classes (Fig. 5c and Extended Data Fig. 11c,d).

We used hierarchical clustering to build a cell-type dendrogram, binarized expression and inferred candidate TFs involved in the key branching events (Methods). Previously identified class A or B excitatory neurons^46^ were generally clustered together for all three species (Fig. 5d and Extended Data Fig. 11e,f). We performed differential gene expression analysis for each class per species to identify TFs conserved across three amniotes (interposed versus medial CN) or two mammals (lateral versus interposed or lateral versus medial CN), identifying *Lmx1a*, *Tbr1*, *Lhx9*, *Prox1*, *Foxp1*,* Foxp2*, *Nr2f1* and* Nr2f2* (Supplementary Table 8). Many of these genes have important roles in the chronology of CN glutamatergic neuron development^48,50–52^ and are members of ohnologues with distinct expression patterns across cell subtypes (Fig. 5e). *Lmx1a* and *Tbr1* were primarily expressed in the medial nucleus, whereas *Lhx9* was mainly expressed in lateral and interposed nuclei (Fig. 5e). Notably, sharks co-expressed *Lhx9* and *Tbr1* during early CN development^53^. Unlike the restricted *Lhx9* expression in chicken, mice showed an expanded *Lhx9* domain, marking regions that developed into the lateral CN^48^. Together, these findings indicate that subsequent changes in these TFs (especially *Lhx9*) have facilitated CN excitatory neuron duplication and divergence. We also found that many ohnologues that encoded axon guidance molecules were differentially expressed (Fig. 5f and Extended Data Fig. 11g,h), a result that reflects their projection to and from the CN^50,54,55^. Clusters of interposed X (IntX, a spatially isolated region in chickens) are considered not to have directly homologous nuclei in mice^46^, and these were clustered with medial nuclei in chickens (Extended Data Fig. 11f). We found several TFs that may be related to IntX specification (Extended Data Fig. 11i) and many were ohnologues. These findings indicate that ancient ohnologues were still involved in the duplication and divergence of potential sister cell types^46,56^ long after WGD.

## Discussion

The identification of two WGDs in early vertebrate evolution has fuelled speculation on their evolutionary importance. We showed that most major vertebrate neural cell-type families originated in the vertebrate ancestor, after divergence from amphioxus but before the cyclostome–gnathostome split. Clear one-to-one cell-type homology between vertebrates and amphioxus was generally lacking, except at higher levels for glia. These ‘diffuse’^57^ or ‘level-dependent’ relationships imply that there was lineage-specific diversification. Older SSD paralogues were more likely to be markers than younger SSD paralogues, which suggests that they had more opportunity to evolve cell-type-specific expression. This may be a consequence of time and/or of differing opportunity during different periods of evolution. However, ohnologues were more likely to be markers than SSD paralogues irrespective of gene age. Analyses of lung and eye data suggest that these are likely to be general properties of vertebrate cell types.

Cross-species comparisons indicated that subfunctionalization was the dominant evolutionary outcome for both duplication types. Although our methods may make subfunctionalization easier to detect than neofunctionalization, this result aligns with several studies^58–60^ (but see ref. ^61^). At either the expression level or the percentage of cells expressing the gene, we observed widespread dosage selection. This result explains the cellular basis for findings from bulk transcriptome analyses^40^. The DDC hypothesis and the gene balance hypothesis offer explanations for these observations.

Cell-type conservation generally straddled vertebrates but not amphioxus. This finding aligns with the relative importance of the 1R WGD, which predated lampreys and contributed more ohnologues than the 2R WGD. The significant association of ohnologues as cell-type markers is indicative of their importance for cell-type evolution, and ohnologues were enriched in TFs and other genes with developmental roles that would be needed for cell-type individuation. Our analyses showed that this association is also found for cell types that evolved much more recently in the CN of amniotes, in which ohnologues also disproportionately contribute to cell-specific properties.

These analyses showed that ohnologues are associated with cell types and contribute to their identities. However, this does not prove that they were necessary. Demonstrating causation in ancient evolutionary processes (the 2R WGDs occurred over 450 million years ago) is inherently challenging. However, establishing amphioxus datasets enabled us to explore this possibility. Models were constructed to predict ancestral regulatory states that reflected whether cell types preceded or post-dated the 1R WGD. Amphioxus data supported the latter model, as did the prevalence of subfunctionalization. Potentially, CoRC patterns like the WGD-first model could be achieved through loss. However, loss more often modifies pre-existing traits rather than generates novelties like new cell types.

We also analysed macroglia, focusing on *SoxE* and associated TFs in astrocytes, ependymal cells and oligodendrocytes. The results suggested that ancestral chordates already had radial glia co-expressing *SoxE*, *Olig* and *Nfia/Nfib/Nfix*. After WGD, ohnologous TFs in these families diverged functionally, which then supported individuation of novel cell types. Notably, the amphioxus *SoxE* mutant showed a much less severe phenotype than vertebrate *SoxE* knockouts. We suggest that this result reflects the broad expression of many key TFs in amphioxus, as described here and reflected in the WGD-first model. That is, ancestral cell types were specified by pools of TF families with functional overlap, and these then individualized into sister cell types in vertebrates through divergence between ohnologues. Notably, knockouts of TFs essential in other species (for example, *Foxj1* (ref. ^62^), *Pax6* (ref. ^63^) and *Mnxa*^64^) in amphioxus also showed weak phenotypes. This result could hence be a generalizable conclusion but needs further study.

It is important to note that WGD is not necessarily linked to cell-type innovation, as WGDs have been identified in other lineages in which evidence for similar innovation is lacking. Early vertebrate evolution may reflect the coincidence of these rare genomic events with the unique evolutionary environment in which the vertebrate phenotype elaborated. WGD will also not be the only route to cell-type innovation. Other changes, for example, in gene regulatory and protein–protein interaction networks, are probably significant, which may themselves have been influenced by WGD.

We conclude that WGDs in early vertebrate evolution played an important part in the evolution of vertebrate neural cell-types and hence of brain complexity. Specifically, the first WGD is linked to the origin of many vertebrate major brain cell types and with the second in gnathostomes adding to this, although to a lesser extent. Our analysis also showed that this effect persisted for hundreds of millions of years after WGD, with ohnologues still important for more recent cell-type diversity changes in the amniote CN. We suggest that these effects will extend beyond brains and reflect a general consequence of these WGDs.

## Methods

### Vertebrate scRNA and snRNA atlas collection, filtering and preprocessing

Cell atlases were retrieved from previous publications^2–5^. Low-quality cells in the human atlas were further filtered based on nCount (UMI) < 400. Low-quality cells in the other atlases were already filtered. To focus on neural cells in the brain, vertebrate datasets were filtered to retain only brain tissues at juvenile or adult stages. To help balance the number of cells for cross-species integration and to accommodate different proportions of neurons and glia, we randomly downsampled the human and lizard atlases to 10^5^ neurons and 10^5^ non-neurons, but retained the full brain atlases for mouse (67,937 neurons and 60,395 non-neuronal cells) and lamprey (18,166 neurons and 41,472 non-neuronal cells). Only protein-coding genes were retained for downstream analyses.

As the original atlases were generated using different pipelines, we applied a standardized preprocessing approach to ensure consistency. We performed SAM analysis on each individual atlas by directly invoking the SAMAP function from the SAMap package (which runs SAM internally)^20^. Specifically, UMI counts from each cell were first normalized to give the median total count per cell, then log_2_-transformed followed by applying the SAM function with the following parameters: preprocessing=“StandardScaler”, npcs=100, weight_PCs=False, k=20, n_genes=3000, weight_mode=‘rms’. The anndata objects were then converted to Seurat format for downstream clustering.

### Amphioxus sample collection, scRNA and snRNA library construction and raw data processing

Amphioxus (*B. floridae*) were obtained from a stock maintained by J.-K. Yu originating from Tampa, Florida. The amphioxus and their offspring were maintained at Xiamen University under previously described conditions^65^. The brain (anterior to the first dorsal ocellus) and neural tube (posterior to the first dorsal ocellus) were dissected as previously described^29^. We constructed and sequenced one scRNA-seq and one snRNA-seq library for each tissue.

For the scRNA-seq experiment, the dissected brain (from ten adult individuals) and neural tube (from eight adult individuals) tissues were respectively washed three times in ice-cold calcium-free and magnesium-free artificial seawater (CMF-ASW)^66^ and then transferred into 500 µl enzyme mix (10% trypsin and 2 mg ml^–1^ collagenase in CMF-ASW) and incubated in a 37 °C incubator with a nutating shaker for approximately 10 min. During digestion, tissues were gently pipetted every 1–2 min to facilitate dissociation, and progress was monitored under an inverted microscope. Digestion was terminated by adding 1 ml of an ice-cold quenching solution (20% fetal bovine serum and 2 mg ml^–1^ glycine in CMF-ASW). Cells were passed through a 40 µm cell strainer and centrifuged at 270*g* at 4 °C for 5 min. The supernatant was removed, and 500 µl RNase-free 0.04% BSA in 3× PBS was added to resuspend the cells. Calcein-AM (BD Biosciences, 564061) was added to the cell suspension to a final concentration of 10 µM and incubated at 37 °C for 5 min. The cells were subsequently placed on ice then immediately processed. scRNA-seq library construction was carried out in accordance with a previous study^29^. The final libraries were sequenced on an Illumina NovaSeq 6000 platform.

For the snRNA-seq experiment, we used a Nucleus Isolation kit (SHBIO, 52009-10) to obtain single nuclei of the dissected tissues. RNase inhibitors (Sigma, 3335399001) were added to the reagents before use. The samples were cut and transferred to a 5 ml tube containing lysate, mixed and lysed for 2 min on ice, then filtered through a 40 μm cell filter (Sigma, BAH136800040). The nucleus count was estimated using a microscope (Leica) with DAPI reagent. After staining with 0.4% trypan blue (Sangon Biotech E607320-0001), the nucleus was observed under a ×40 microscope (Jiangnan Novel Optics XD-202). Subsequent experiments were performed if the nuclear envelopes were intact and there were few impurities. snRNA-seq libraries were prepared using a SeekOne DD Single Cell 3′ library preparation kit (SeekGene, K00202). In brief, an appropriate number of cell nuclei was mixed with reverse transcription reagent and then added to a sample well in a SeekOne DD chip S3. Subsequently, barcoded hydrogel beads and partitioning oil were dispensed into corresponding wells separately in the chip S3. After emulsion droplet generation, reverse transcription was performed at 42 °C for 90 min and inactivated at 85 °C for 5 min. Next, cDNA was purified from broken droplets and amplified by PCR. The amplified cDNA product was then cleaned, fragmented, end-repaired, A-tailed and ligated to a sequencing adaptor. Finally, indexed PCR was performed to amplify the DNA representing the 3′ polyA part of expressing genes, which also contained the cell barcode and the unique molecular index. The indexed sequencing libraries were cleaned up using VAHTS DNA Clean Beads (Vazyme N411-01), analysed by a Qubit (Thermo Fisher Scientific, Q33226) and a Bio-Fragment Analyzer (Bioptic, Qsep400). The libraries were then sequenced on a GeneMind SURFSeq 5000 with PE150 read length.

Raw reads from scRNA-seq were processed using the BD Rhapsody WTA analysis pipeline (v.1.12.1; https://bitbucket.org/CRSwDev/cwl/src/master/) on the Seven Bridges platform (https://sevenbridges.com/). Raw reads from snRNA-seq were processed using the SeekSoul Tools pipeline. scRNA and snRNA expression matrices for each sample were then filtered and processed using Seurat (v.5.0.0). Cells or nuclei with fewer than 300 detected genes, more than 4,000 detected genes, more than 10,000 UMI detected or more than a 10% MT expression ratio were filtered out (we used stricter parameters for neural tube processed by SeekGene due to its higher ambient RNA).

### Clustering and annotation

To find good-quality and high-resolution cell clusters in the SAM preprocessed atlases, we performed hierarchical and iterative clustering for individual vertebrate cell atlases using the scrattch.hicat and scrattch.bigcat packages^67,68^ from the Allen Institute. Raw counts (UMI) were first normalized using the cpm function provided in the above packages, followed by log_2_ transformation with a pseudo-count added to prevent log_2_[0]. Cells were initially classified into broad groups and hierarchically clustered on the basis of the expression of highly variable genes, principal component analysis and Jaccard–Louvain clustering. Clustering was performed iteratively in each group using the iter_clust function, continuing until no further subclusters satisfied predefined thresholds for the number of DEGs or minimum cluster size. As our analysis did not aim to resolve extremely fine-scale cell types, we applied more relaxed parameters than those typically used with this method. DEG thresholds were defined via the de_param settings: padj.th = 0.05, q1.th = 0.4, q2.th = NULL, q.diff.th = 0.5, de.score.th = 100, min.cells = 100, and min.genes = 6. Dimensionality reduction and clustering parameters were specified as follows: dim.method = “pca”, max.dim = 80, method = “louvain”. Minimum cluster sizes were set via split.size as 800, 500, 500 and 500 for human, mouse, lizard and lamprey datasets, respectively. As we did not aim to study cell types at very high resolution, we tuned the split.size parameters for each species to generate cluster numbers at a similar level across vertebrates. Clusters were then checked and merged at the end of the iteration to ensure that they were separable with scrattch.bigcat::merge_cl. We simply used Seurat FindClusters with the Louvain algorithm and resolution = 1 for amphioxus owing to the limited number of cells and nuclei in the datasets.

We next confirmed and refined the annotation of individual vertebrate atlases by examining the expression of canonical markers (Supplementary Table 1 and Supplementary Fig. 2a–d), reference annotation in our clustering and their main dissection locations for vertebrates (Supplementary Table 2). We annotated amphioxus brain cell types on the basis of markers (Supplementary Table 1 and Supplementary Fig. 2e), mapped them to CNS cell types at the late neurula stage with MetaNeighbor (Extended Data Fig. 2a) and summarized the data into Supplementary Table 2.

### Atlas integration and cross-species mapping

Homologous gene relationships for initial weighting gene–gene graphs with cross-species edges in SAMap were generated by blast on protein-coding genes using SAMap map_gene.sh. We then performed cross-species mapping using the SAMap run function with five iterations, with the edge weight calculated and updated by Pearson’s correlation (hom_edge_mode = “pearson”) and 30 cross-species edges per cell (crossK = 30). Mutual nearest neighbourhoods were independently calculated between each pair of species (pairwise=True). For chordate comparison, we randomly downsampled 1,500 cells for each major cell-type family in vertebrates. We then used the same parameters for SAMap mapping in chordates but with crossK = 20 owing to the low cell numbers in the amphioxus data. The alignment scores between cell types across species were calculated using get_mapping_scores from SAMap. We next used the GenePairFinder function to identify gene pairs (genes between species) that positively contributed to cross-species correlation between cell types and were differentially expressed in respective atlases.

### Identification of cell-type-specific TFs and conserved sets for cell-type families

To identify TF-coding genes for each species, we used DeepTFactor^69^, a deep-learning-based tool optimized for TF prediction. Cell-type-specific TFs were identified for each major cell-type family in vertebrates using NS-Forest (v.4.0)^70^, a method designed to identify minimum combinations of necessary and sufficient marker genes for distinguishing different cell types. This method uses a random forest algorithm on preselected genes by binary scoring, a measurement of binary expression (specificity) for a gene. For our analysis, we used the binary score to rank TF specificity and extracted the top 30 TFs with the highest scores as cell-type-specific TFs with the nsforesting.NSForest function and the following parameters: gene_selection = “BinaryFirst_high”, n_top_genes = 30, n_binary_genes = 30, n_trees = 1500.

We next assigned cell-type-specific TFs to individual orthogroups and defined an orthogroup as a conserved TF orthogroup for cell-type families if at least three (out of four) vertebrates contained these TFs. This approach identified 81 orthogroups (Supplementary Table 3-1), which we manually reviewed for expression patterns across species and summarized in Supplementary Table 3-2 with supporting references. The orthogroups generated by OrthoFinder were uploaded to GitHub (https://github.com/DiracZhu1998/WGD2celltype_evolution).

For the analysis of conserved TF orthogroups between sister cell types (Fig. 3, related analysis), we first subset 5,000 cells per group and identified markers for each sister cell type using Seurat FindAllMarkers with only.pos = T at individual species. For the comparison between oligodendrocytes and ependymo-astrocytes, we limited our analysis to oligodendrocytes and astrocytes, as ependymal cells are far less numerous than astrocytes. Markers were then filtered with adjusted *P *< 0.05 and average log_2_[fold change] ≧ 0.58 and percentage of cells expressing that gene in the foreground > 0.1. We retained only TFs and defined an orthogroup as a conserved TF orthogroup for astrocytes versus ependymal cells if at least three (out of four) vertebrates contained these TFs. For the comparison between astrocytes and oligodendrocytes and between astrocytes and OPC, a conserved TF orthogroup was considered conserved if at least two (out of three) amniotes contained these TFs.

### Classification of TFs and TF enrichment analysis

TF families were downloaded from AnimalTFDB (v.4.0)^71^ (http://guolab.wchscu.cn/AnimalTFDB4/#/Download). To classify TF gene families in species not represented in the database, we assigned TF family classifications at the orthogroup level using OrthoFinder output. If any human gene in an orthogroup was annotated with a specific TF family, we classified the entire orthogroup under that family. The high overlap (>90%, not shown) in classifications based on model organisms (human, mouse and zebrafish) validated the robustness of this approach. The enrichment of a TF class was assessed using hypergeometric tests with the stats::phyper function for each TF class in each species. *P* values were further adjusted using the p.adjust(method = “fdr”) from the R Stats package.

### Identifying gene relationships for orthologues, paralogues, ohnologues and SSD paralogues

To identify gene relationships, we first collected genome assemblies and gene annotation files for the species listed in Supplementary Table 9. For each protein-coding gene, only the transcript with the longest coding sequence (CDS) was retained. CDSs were extracted from genomes based on gene annotation files and translated into proteins with in-house scripts. We then performed phylogenetic orthology inference with OrthoFinder (v.2.5.5)^72,73^. The species tree inferred from orthogroups matched with references (data not shown). Orthologues were identified on the basis of OrthoFinder output, applying a reciprocal best hit criterion. (In-)paralogues were defined as duplicated genes in the same orthogroup for each species. Ohnologues were identified based on Ohnologs (v.2.0)^74^ (details provided at GitHub (https://github.com/SinghLabUCSF/Ohnologs-v2.0), together with updates of ohnologues used (https://github.com/DiracZhu1998/WGD2celltype_evolution/tree/main/2.gene_relationships/ohnolog_inferring and see Supplementary Text for the evaluation of ohnologue detection) with a similar number of vertebrates used and updated genome and annotations. Owing to the limited availability of data for jawless vertebrates and the extensive loss of duplicated genes in this lineage^1^, ohnologue identification in lamprey remains challenging. As jawed and jawless vertebrates independently underwent the second round of WGD, we tried ohnologue detection with lamprey and without the inclusion of lamprey and found little difference between the outcomes (<0.2%). We also tried two other methods, doubletrouble^75^ and DupGen_Finder^76^, but they were not comparable to Ohnologs (v.2.0) or with previous results^1^ regarding the number of identified ohnologues and stability in different vertebrates (see details of the comparison in the Supplementary Text). Nevertheless, recent studies^7,30^ suggest that the second round of WGD in jawed vertebrates probably involved interspecific hybridization, which resulted in asymmetric gene loss. Specifically, genes from the alpha parental lineage were around four times more likely to be retained than those from the beta lineage (based on results in chicken; https://raw.githubusercontent.com/fmarletaz/hagfish/refs/heads/main/Paralogons/Vert_Evt_OGrrA.txt).

To assess the robustness of our ohnologue predictions, we compared our results to the Ohnologs (v.2) database (http://ohnologs.curie.fr), finding that 75% of human and 70% of mouse ohnologues in our dataset were also present in the database, and vice versa (see the methodology comparison in the Supplementary Text for more details). SSD paralogues were defined as paralogues that are not ohnologues.

### Paralogue gene age classification

Protein sequences were generated as described above. We aligned two protein sequences for each ohnologue and SSD paralogue pair using MAFFT (v.7.520)^77^ with the L-INS-I option (--localpair --maxiterate 1000) and converted the protein alignment into a codon alignment using PAL2NAL^78^. Then we used KaKs_Calculator (v.2.0)^79^ to calculate *K*_a_ (the rate of nonsynonymous substitutions), *K*_s_ (the rate of synonymous substitutions) and *K*_a_/*K*_s_ values. *K*_a_ and *K*_a_/*K*_s_ values were used in other analyses. *K*_s_ between paralogue pairs is used to estimate their duplication time^76^. For SSD paralogues, we estimated the duplication age based on a previous simulation in which *K*_s_ = 0.01 per million years (in other words, *K*_s_ = 1 is approximately 100 million years ago)^80^. We also retrieved duplication age information from Ensembl BioMart based on gene trees and compared the two metrics, which showed overall consistency (data not shown). It is worth noting that both approaches involve some imprecision: gene trees depend on the available taxa and on thresholds used to cluster genes into trees (similar to orthogroup classification), whereas *K*_s_ reflects the onset of divergence between duplicates (that is, related to the rediploidization time^81^).

As the two rounds of WGD in early vertebrate evolution are so close, we cannot use this method to separate 1R and 2R ohnologues. We instead separated ohnologues in jawed vertebrates into alpha and beta categories based on chicken orthology assignments from previous work^1^.

### Identification of marker genes at the cell-type family and cluster level

To reduce the potential influence of imbalanced cell-type numbers in vertebrates, we randomly subsampled 3,000 cells for each category during marker identification. Owing to the limited cell numbers in amphioxus clusters, we did not subsample amphioxus clusters during marker detection. Marker genes were identified for each species using the FindAllMarkers function of Seurat (v.5.0.0)^82^ with the Wilcoxon rank-sum test (min.pct = 0.01, logfc.threshold = 0.58, test.use = ‘wilcox’, only.pos = TRUE) at both the cell-type family level and cluster level. For related downstream analyses, only marker genes with FDR < 0.01 were used. As a few studies^83,84^ previously questioned the quality of the Seurat ‘wilcox’ output, we also identified markers using FindAllMarkers with ROC analysis (test.use = “roc”, only.pos = TRUE), which led to the same conclusions (data not shown but listed in GitHub and Figshare).

### Gene regulatory network analysis

We performed gene regulatory network analysis and identified regulons using pySCENIC^85,86^. To reduce noise introduced by the imbalance in the number of cells in each major cell-type family, we first randomly subset 2,000 cells for each major cell-type family. To reduce noise of lowly expressed genes, we filtered genes expressed by fewer than 0.5% of cells and with low total UMI (equivalent to 1 UMI detected in 1% cells).

The grn command in pySCENIC was used to infer gene–gene co-expression relationships between TFs and their potential target genes with grnboost2 algorithm. This process returned an adjacency edge list with the TF, its potential target gene and an associated importance score. The adjacency edge list was then used as input for the ctx command to identify regulons, each consisting of a TF and its target genes enriched for the binding motifs of the TF. Human and mouse TF lists were downloaded (https://resources.aertslab.org/cistarget/tf_lists/). The ctx command uses a motif annotation database and ranking databases, both of which were downloaded from Aerts Laboratory’s cistarget resources (motif ranking datasets: https://resources.aertslab.org/cistarget/databases/old/homo_sapiens/hg38/refseq_r80/mc9nr/gene_based and https://resources.aertslab.org/cistarget/databases/old/mus_musculus/mm9/refseq_r45/mc9nr/gene_based/; and motif annotation files: https://resources.aertslab.org/cistarget/motif2tf/). Next, the aucell command was used to compute regulon activity scores for each major cell-type family, and a regulon specificity score (RSS) was calculated using the regulon_specificity_scores function. The top regulons for each cell type were selected on the basis of the RSS.

### GO annotations and enrichment analyses

Owing to the lack of recent updates for the GO annotation of lizard (*Pogona vitticeps*) lamprey (*Petromyzon marinus*) and amphioxus (*B. floridae*), we re-annotated the GO annotations for these three species. GO annotations for the protein-coding genes of model organisms (*Danio rerio*, *M. musculus* and *H. sapiens*) were downloaded from Ensembl through BioMart. GO terms were associated with protein-coding genes from *Pogona vitticeps*, *Petromyzon marinus* and *B. floridae* according to their one-to-one orthologues in *H. sapiens*, *M. musculus* and *D. rerio* in an order of priority (human > mouse > zebrafish). The lizard, lamprey and amphioxus genes that could not be annotated using the above method were then BLAST-searched to the UniProtKB database^87^ (release-2024_03) using BLAST (2.9.0+)^88^ with parameters (-evalue ×10^–8^). The best hit for each query was selected based on a bit score and its corresponding GO terms (ftp://ftp.ebi.ac.uk/pub/databases/GO/goa/UNIPROT/goa_uniprot_all.gaf.gz) assigned to the respective query. In total, we annotated nearly all protein-coding genes for lizard and over 70% of lamprey and amphioxus. This level of annotation is higher than for GO in Ensembl for another amphioxus species, *Branchiostoma lanceolatum*, for which more than half of the protein-coding genes are not functionally annotated.

The datasets of GO annotations for lizard, lamprey and amphioxus were built using makeOrgPackage function from the AnnotationForge package^89^. The dataset packages for human and mouse were retrieved from Bioconductor (v.3.20) at org.Hs.eg.db^90^ and org.Mm.eg.db^91^, respectively. GO enrichment analysis was performed with clusterProfiler^92^ enrichGO with Benjamini–Hochberg adjustment and cutoff = 0.05. Only protein-coding genes expressed in corresponding datasets were used as background genes in the GO enrichment analysis. The redundancy of enriched terms was filtered by simplify() with the following parameters: cutoff=0.7, by = “p.adjust”, select_fun=min.

### Classification of protein class and over-representation analysis

To investigate protein class in ohnologues and SSD paralogues, we used ‘Functional classification viewed in graphic charts’ with bar plots in the PANTHER database^93^ (v.19.0; https://www.pantherdb.org). Over-representation analysis was conducted using the ‘Statistical Overrepresentation Test’ in the PANTHER website, with protein-coding genes in our datasets as the background. Fisher’s exact test was applied with FDR correction to assess significance. Owing to the absence of corresponding data for lizard, lamprey and amphioxus in the PANTHER database, this analysis was limited to human and mouse.

### Cross-species cell-type tree

We filtered orthogroups to retain those containing at least one gene in each of the five species. To minimize the potential influence of high copy-number SSDs, only orthogroups with fewer than or equal to five gene copies for each species were retained. We defined metagenes by summing the UMI counts of all gene copies in each orthogroup for each species. Expression normalization and identification of 3,000 highly variable metagenes were performed using Seurat’s SCTransform function^82^. We retained metagenes that were both highly variable in at least three out of the five species and were TF metagenes (if TFs were in that metagene or orthogroup).

Cross-species comparisons of cell-type-specific gene expression were based on gene specificity indices calculated using a previously developed method^16^. In brief, for each metagene *g* in cell-type *c*, we computed its specificity index (*s*) as the mean expression in *c* divided by its mean expression across all cells:

$${s}_{g,c}=\frac{{g}_{c}}{\left(\frac{1}{N}\sum _{i\in c}{g}_{i}\right)}$$

This formula shows that the number of cells per category matters. To control the cell number imbalance in cell types, we subsampled 500 cells per glial cell-type family in vertebrates and per glia cell type in amphioxus. We then generated a chordate glia tree using pvclust with the following parameters: nboot = 1000, method.hclust = “average”, method.dist = function (z){as.dist (1 - cor (z, use = “pa”, method = “spearman”))}.

### RNA velocity and multipotency analyses in amphioxus

We performed RNA velocity based on velocyto.py (v.0.17)^94^ and scVelo (v.0.3.3)^95^. Each sample from N4 and T1 stages was preprocessed to generate a loom file with annotated spliced and unspliced reads, and loom files for the same developmental stage were merged and analysed together. Both spliced and unspliced raw counts (UMI) were first normalized using scvelo.pp.normalize_per_cell, and the top 3,000 genes with the highest variance were selected, followed by log-transformation via scvelo.pp.log1p. Dimensionality reduction and neighbourhood smoothing were performed using scvelo.pp.moments with parameters n_pcs=50, n_neighbors=30. For dynamical modelling of transcriptional kinetics, we applied scv.tl.recover_dynamics and subsequently executed scv.tl.velocity(mode = ‘dynamical’).

To assess cell-type multipotency, amphioxus gene IDs were mapped to one-to-one mouse orthologues based on reciprocal best hit, and CytoTRACE2 (ref. ^96^) was applied with the parameters species = “mouse”, seed = 42.

### Generation of amphioxus *SoxE* mutants

CRISPR–Cas9-mediated gene editing was used to generate *SoxE* mutants as previously described^97^. A gRNA targeting the sequence (5′-GGCCCATGAACGCCTTCA-3′) at the beginning of HMG-encoding region was selected and synthesized. A PCR primer pair (forward: 5′-TGAGTTTAGCGGCGATCAGT-3′; reverse: 5′-TAGTTTCCCCAGCGTCTTGC-3′) spanning the target site was used to amplify the genomic region. The amplicon was digested with the restriction enzyme XmnI (5′-GAANNNNTTC-3′) to determine the gRNA efficacy and to identify the heterozygous and homozygous mutants. Heterozygotes carrying an 8 bp deletion in the target site were screened and used for the study. Homozygotes were acquired by crossing the heterozygotes.

### In situ hybridization chain reaction

Expression patterns of *SoxE*, *OligB*, *Eaat2* and *Syn* were detected by HCR (v.3) as previously described^98^. The probe sequence information is provided in Supplementary Table 10. DAPI (Invitrogen, 1 mg ml^–1^ in PBST) was used for nuclear staining. After staining, the samples were stored in antifluorescence quencher medium (S2100, Solarbio) and photographed under a confocal laser scanning microscope (LSM980 Airyscan2, Zeiss).

### Single-embryo bulk RNA-seq and downstream analyses

Gonadally mature *SoxE* heterozygous F_1_ female and male amphioxus were subjected to the thermo-based method (from 19 to 29 °C) to produce gametes. Fertilized eggs were incubated in an incubator maintained at 30 °C and 95% humidity, in which embryos developed to the N4 and T1 stages. At each stage, 15 embryos were randomly selected and each embryo was carefully placed into a PCR tube, with efforts to remove seawater while ensuring embryo survival. The PCR tube containing one embryo was snap-frozen in liquid nitrogen for 10 min, and the samples were subsequently stored at −80 °C. The samples were later sent to Tenk Genomics for Smart RNA extraction, Smart-seq2-based RNA reverse transcription, cDNA quality assessment, amplification, purification and quantification. The cDNA was returned for *SoxE* genotype identification. We designed another pair of PCR primers (forward: 5′-GAGCCCACCGAGCTCGA-3′; reverse: 5′-TAGTTTCCCCAGCGTCTTGC-3′) to amplify the *SoxE* gRNA2.5 target site and used the restriction enzyme XmnI for genotype analysis of each sample. Three wild-type and three mutant samples from the N4 and T1 stages were selected for sequencing library construction using Nextera technology by Tenk Genomics. Sequencing was performed on a BGI T7 platform with a PE150 mode, and the sequencing depth was 6 Gb. Sequencing statistics are provided in Supplementary Table 11 and Supplementary Fig. 22. Genotypes were further confirmed by read alignments (Supplementary Fig. 21). After the removal of low-quality reads and adapter sequences using fastp (v.1.0.1)^99^, clean reads were aligned to the amphioxus genome using STAR (v.2.4.0g1)^100^ with a maximum intron length of 10,000 bp (--alignIntronMax 10000), optimized for amphioxus. Gene raw counts of each sample were calculated using featureCounts (v.2.1.1)^101^ from BAM for DESeq2 (v.1.42.0)^102^ normalization and differential gene expression analysis.

### Subfunctionalization and neofunctionalization

Gene relationships were assessed on the basis of the above-described OrthoFinder output. To avoid risks of skewing from high copy-number SSDs and uncertainty on orthology inference due to gene turnover, only orthogroups with fewer than or equal to five gene copies for each species were retained. To infer ancestral states without considering gene losses (loss of all copies), we retained orthogroups with at least one copy for each species. This enabled us to do cross-species comparisons directly at the orthogroup level as at least one gene for each species was present for each orthogroup. An orthogroup was classified as an ohnologue orthogroup if it contained one pair of ohnologues in at least three out of four vertebrates, which resulted in 1,872 ohnologue orthogroups. The same approach was applied to SSDs, and 1,050 SSD paralogue orthogroups were identified. Some (339) orthogroups were considered as both an ohnologue orthogroup and SSD paralogue orthogroup.

To predict ancestral states, we next binarized expression matrices using two separate approaches: based on whether genes were classified as markers, and based on gene expression or not determined by the Trinarization score. For the second approach, a gene was considered expressed if it was estimated to be present in at least 10% of the cells, with a posterior error probability of no more than 5%. Details of the Trinarization score have been previously described^3^. We inferred ancestral states for vertebrate and amniote lineages across homologous cell-type families. For vertebrate ancestral states, a gene family was considered expressed (state = 1) in a given cell-type family if at least three out of four species used one or more copies from that paralogue family in that cell-type family. The same criterion was applied for predicting amniote ancestral states, whereby expression (state = 1) was assigned if at least two out of the three species were being considered. The extent of subfunctionalization and neofunctionalization in gene families was quantified by comparing the binarized expression patterns of individual genes to the inferred ancestral states. Specifically, the difference between the binarized expression of a gene and orthogroup ancestral state was computed, in which a value of –1 indicated subfunctionalization (unless all copies in that species were –1, which indicated loss of function; Fig. 4a) and a value of +1 denoted neofunctionalization.

### Expression divergence (d*T*) among paralogues

Gene relationships were based on the above-described OrthoFinder output. To avoid risks of skewing from high copy-number SSDs and uncertainty on orthology inference due to gene turnover, only orthogroups with fewer than or equal to five gene copies for each species were retained. To perform the pairwise comparison in shared orthogroups, orthogroups with at least one copy for any of the four species were further retained. Paralogue orthogroups were then defined as orthogroups that included one pair of paralogue genes in at least three out of four vertebrates. For a combination of paralogues in orthogroup, we calculated the expression divergence (d*T*) based on a simple formula^103^ for each species separately. Specifically, d*T* was first calculated for each pair of paralogues by the fractional difference between the number of cell-type families expressing either paralogue (*N*_either_) and the number of cell-type families expressing both paralogues (*N*_both_) relative to *N*_either_. d*T* was next averaged in a paralogue orthogroup (when there was more than one pair of paralogues) for each species.

### Cell-type nonspecific dominant expression

To compare gene expression levels between paralogues for each species, we first calculated the average normalized expression levels for each gene using the Seurat::AverageExpression function^82^, and the proportion of cells expressing specific genes (pct. exp.) with an expression count greater than 0. These calculations were performed at both the cell-type family and cluster levels. Next, we use the igraph package^104^ to construct ohnologue and SSD paralogue families based on previously identified ohnologue pairs and SSD paralogue pairs, respectively. We tested the expression levels and pct. exp. values using the friedman_test function from the rstatix package^105^, as the data did not follow a normal distribution. For species pairwise comparisons in individual ohnologue and SSD paralogue families, we applied the rstatix::wilcox_test function with Bonferroni-adjusted *P *values to identify the highest-expressed (dominant) copy in each gene family and to search for whether their orthologues are the dominant copy in another species. One-to-one orthologue relationships underpinning this were derived from above-described OrthoFinder results.

### Variance decomposition and the identification of genes highly contributing to cell-type and/or regional identity

To assess the contribution of a gene to cell-type identity and regional identity, we constructed a sum of UMI in expression matrices with three major cell-type families in the brain (excitatory neurons, inhibitory neurons and astrocytes) along with four brain divisions (telencephalon, diencephalon, mesencephalon and rhombencephalon). The pseudobulk expression was calculated by the sum of gene counts (UMI) for each gene in individual cell-type families. To balance cell number differences, 2,000 cells were randomly selected for each cell-type family in each species. We then used DESeq2 (ref. ^102^) to normalize library sizes and performed LMM for each gene with the lme4 package^106^. The restricted maximum likelihood estimators for the random effects of cell-type, regional and residual variance were normalized by their sum to give the variance components (Extended Data Fig. 9c). Genes that contributed >25% of the total variance to cell-type family or regional identity were classified as genes that highly contributed to cell-type signals and regional signals, respectively.

### Analysis of the CN

We downloaded human, mouse and chicken CN datasets^46^. These datasets were further filtered to retain only protein-coding genes and excitatory neurons, which show higher regional variants than inhibitory neurons in the CN. We detected DEGs as described above, using FindAllMarkers with parameters (wilcox, only.pos = TRUE), and only DEGs with log_2_[fold change] > 0.58 and adjusted *P* < 0.01 were retained. Scaled average expression was calculated using Seurat AverageExpression^82^ and then normalized by dividing the expression of each gene by its mean among different cell types. The transcriptomic dendrogram was calculated on the basis of scaled average expression of DEGs using pvclust with the following parameters: Spearman’s correlation-based distance 1 – cor() and average linkage with 1,000 bootstrap replicates. Expression profiles were binarized using the Trinarization score, and a gene was considered expressed if it was estimated to be present in at least 20% of the cells, with a posterior error probability of no more than 5%. We used a 20% threshold here rather than the 10% applied in the previous analysis because several documented CN-related TFs showed substantial differential expression with more than 10% cells expressing the gene. For comparisons involving serially homologous structures, such as distinct CN types, a more permissive cutoff was appropriate to avoid excluding biologically meaningful signals. The binarized data were then used to infer ancestral states based on dendrograms using maximum parsimony. Specifically, we used the phangorn package^107^, converted the binarized expression into phyDat format and applied the ancestral.pars function with the accelerated transform (ACCTRAN) approach to estimate ancestral states and return probability. Genes in each ancestral node were classified as expressed if the probability exceeded 0.5, and as not expressed otherwise. Finally, we identified gene expression gain and loss events along branching points in the tree to identify candidate genes that might be involved in the cell-type duplication and divergence.

### Ethics approval

Work with lamprey embryos was approved by the University of Oxford, Department of Zoology Animal Welfare and Ethical Review Board. Ethical review was not required for work with amphioxus.

### Reporting summary

Further information on research design is available in the Nature Portfolio Reporting Summary linked to this article.

## Online content

Any methods, additional references, Nature Portfolio reporting summaries, source data, extended data, supplementary information, acknowledgements, peer review information; details of author contributions and competing interests; and statements of data and code availability are available at 10.1038/s41586-026-10629-x.

## Supplementary information

Supplementary Information This file contains Supplementary Text (additional analyses and validation supporting the main results), Supplementary Figs. 1–22 and Supplementary References.

Reporting Summary

Supplementary Table 1 Marker genes and relevant citations for vertebrates and amphioxus.

Supplementary Table 2 Refined cluster annotation in all five species.

Supplementary Table 3 Conserved TF genes, orthogroups and relevant citations.

Supplementary Table 4 Key conserved TF genes and orthogroups between macroglia types.

Supplementary Table 5 Macroglia markers.

Supplementary Table 6 Genes contributing to regional and cell-type identity.

Supplementary Table 7 CN markers and ohnologues.

Supplementary Table 8 Key conserved TFs for CN excitatory neurons.

Supplementary Table 9 Datasets used for gene tree construction and ohnologue identification.

Supplementary Table 10 In situ hybridization probe details.

Supplementary Table 11 Amphioxus bulk RNA-seq data details.

Peer Review File

## Data Availability

The reference genome, gene models, functional annotations of protein-coding genes, full marker gene list of each cell cluster and important intermediate files are deposited in a Figshare repository (10.6084/m9.figshare.29327111)^108^. All amphioxus bulk RNA-seq, scRNA-seq and snRNA-seq data, as well as lamprey embryonic neural tube scRNA-seq data, have been deposited in the CNCB database under BioProject accession number PRJCA059549. Data analysed from publicly available sources are described in the paper, available from GitHub and Figshare repositories and other online resources, and are listed as follows: (1) human brain atlas (human_adult_GRCh38-3.0.0.h5ad) from https://github.com/linnarsson-lab/adult-human-brain; (2) mouse brain atlas (l5_all.loom) from http://mousebrain.org/adolescent/downloads.html; (3) lizard brain atlas (Pogona_vitticeps_cells_Science_2022.rds) from https://datashare.mpcdf.mpg.de/s/WBX59YhJKKebizb#editor; (4) lamprey brain atlases (adult_diencephalon.rds, adult_rhombencephalon.rds, adult_mesencephalon.rds, adult_telencephalon.rds and lamprey_adult_whole_brain.rds) from https://downloads.kaessmannlab.org/lamprey/; (5) human eye and lung scRNA, preprocessed by the Human Cell Atlas, from https://datasets.cellxgene.cziscience.com/64175889-d600-4b58-97ea-e74be80206e5.rds and https://datasets.cellxgene.cziscience.com/b351804c-293e-4aeb-9c4c-043db67f4540.rds; (6) three species of CN datasets (GSM4873765_mouse_data.RData.gz, GSM4873766_human_data.RData.gz and GSM4873767_chicken_data.RData.gz) from https://www.ncbi.nlm.nih.gov/geo/query/acc.cgi?acc=GSE160471; and (7) Ohnologs (v.2) database (http://ohnologs.curie.fr).

## Code availability

All code and scripts associated with this analysis are publicly available from the GitHub repository (https://github.com/DiracZhu1998/WGD2celltype_evolution).
