# Supplementary material for: Whole-genome duplication shaped cell-type evolution in the vertebrate brain
Source: Nature. 2026 Jun 10;656(8128):659–69. doi: 10.1038/s41586-026-10629-x (PMC13489949; doi:10.1038/s41586-026-10629-x)
Supplement: Supplementary file 1 [file 41586_2026_10629_MOESM14_ESM.pdf]

nature portfolio

Peer Review File

Whole genome duplication shaped cell-type evolution in the vertebrate brain

Corresponding Author: Professor Sebastian Shimeld

Any redactions in this file are there to maintain patient confidentiality, the confidentiality of unpublished data, or to remove third-party material.

This file contains all reviewer reports in order by version, followed by all author rebuttals in order by version.

Version 0:

Reviewer comments:

Referee #1

(Remarks to the Author)
This is an extensive study by Zhu and colleagues where the authors take advantage of single-cell RNA-seq datasets from multiple vertebrate species to explore the contribution of whole genome duplications to brain cell type evolution.

The scale of the analysis performed by the authors is impressive, and the use of comparative single-cell genomics to address a longstanding question in vertebrate evolution is a strength of the work. The study focuses on four main species: human, mouse, lizard, and lamprey- with some amphioxus data which is used as control. The central hypothesis tested is that WGDs, more than small-scale duplications (SSDs), contributed to the diversification of vertebrate brain cell types.

The results begin with a phylogenetic mapping of major neural cell type families, showing that most major vertebrate brain cell type families are already present before the cyclostome-gnathostome split. They note that some families, including oligodendrocytes and cerebellar granule cells, are more recent, emerging within jawed vertebrates and being absent from lampreys.

The delineation of these families is in itself an interesting contribution, providing a nice framework for comparing brain cell type diversity across vertebrates. However, here I missed a better analysis of the control group (the amphioxus) as an examination of the gene families that already existed prior to the genome duplications. While the authors state that "most major neural cell type families predate the cyclostome-gnathostome split," they do not present data on the presence or absence of these same cell type families prior to 1R/2R.

The next analysis quantifies paralog retention among cell type markers, revealing that ohnologues are overrepresented compared to SSD paralogs across the majority of cell type families. The authors found that this enrichment is especially pronounced for transcription factors and other developmental regulators.

Here I missed more detail on the identification of ohnologues versus SSD paralogues (total numbers of genes in each group?) and whether they were matched for set size in the enrichment analyses. Without this information, it was difficult for me to rule out the possibility that the observed enrichment simply reflects differences in baseline abundance between the groups rather than a true functional bias (Figure 1D shows equivalent numbers of genes for WGD and SSD under transcriptional regulators – is this correct?).

Next, the authors expression divergence between paralogues across cell types and find that subfunctionalization is the most common event for both ohnologues and SSD paralogs. Clear cases of neofunctionalization, where a paralog acquires a novel expression domain, are comparatively rare.They also observe that over two-thirds of duplicate families have one copy consistently more highly expressed than the other across cell types. This pattern is interpreted as evidence for dosage selection under the gene balance hypothesis. The authors also explore dosage sensitivity, reporting that ohnologues tend to be retained and expressed in a dosage-balanced fashion more often than SSD paralogues.

Finally, the authors examine cell type origin timing relative to gene duplication events, finding that cell type families that

arose after WGDs show preferential incorporation of ohnologues, and that paralogue pairs can be linked to novel cell type identities. These patterns are interpreted as evidence that WGD provided a genomic substrate for both the emergence of entirely new cell types and the diversification of existing ones.

While these observations are intriguing, I found that the overall framing of the paper strongly favors WGD as the primary driver of new cell type emergence without adequately testing alternative explanations. One competing hypothesis is that increases in regulatory complexity, such as the elaboration of higher-order regulatory network configurations, were the dominant force in generating new cell types. Without analyses that explicitly test for changes in network rewiring, the conclusions about WGD's causal role are not fully supported.

The study's framing of WGD versus SSD as the key drivers of new cell types risks overlooking other, equally important mechanisms that generate regulatory complexity.

The reliance on adult brain transcriptomic snapshots further limits the interpretation of these results. Cell type molecular profiles in the adult reflect the outcome of developmental programs, many of which involve the sequential activation and repression of paralogs over time. By ignoring the temporal dimension of cell type specification, the study risks missing instances where duplicates have critical developmental roles but little or no expression in the adult. Paralog subfunctionalization often occurs along developmental time axes rather than in the final adult state, and without developmental single-cell data, this dimension of WGD influence remains unexplored.

I appreciate the challenge the authors face in light of this problem - as following developmental trajectories and mapping cell type specific GRNs involved is a big task. It would require combining data from multiple stages and species to track how paralogues are used over time. Still, without this developmental view, it is hard to fully answer the study's main question.

A further concern is that (if I understand this correctly) the study pools all ohnologues into a single category, implicitly treating the two rounds of vertebrate WGD (1R and 2R) as equivalent. 1R predates aligns more plausibly with the origin of conserved vertebrate brain cell type families, whereas 2R likely postdates most of these and may have contributed primarily to gnathostome-specific innovations such as oligodendrocytes or certain cerebellar cell types. Without distinguishing between 1R- and 2R-derived paralogues, the claim that "WGD drove vertebrate brain cell type evolution" risks overstating the role of the second round of duplication.

(Incidentally, the discussion falls short in connecting the timing of genomic events to the evolutionary history of the cell type families. By not explicitly considering whether particular cell type families arose before, between, or after the two WGDs, the narrative leaves unclear how the sequence of duplications and other genomic changes maps onto the appearance of new cellular identities.)

Furthermore, if WGDs inherently drive the emergence of new cell types, one might expect lineage-specific WGDs in other clades (such as the cyclostome hexaploidization, the teleost duplication, or the salmonid WGD) to also give rise to novel cell type families. This seems to be implied by the title/abstract. However, the manuscript does not address this prediction, and the absence of such evidence could suggest that WGD's effect is context-dependent, with the first vertebrate WGD playing a unique role in early neural diversification, and later WGDs acting mainly to fine-tune or diversify existing cell types within a lineage.

Referee #2

(Remarks to the Author)
Shimeld et al. present a comparative analysis of single-cell transcriptomic data from four vertebrate brains, with additional outgroup data, to examine the contribution of whole genome duplication (WGD) to the evolution of neural cell types. The authors define conserved cell type families across species, identify enriched transcription factors, and find that ohnologues are overrepresented among cell type markers. They interpret this as evidence that WGD played a foundational role in the diversification of brain cell types, and that its impact has persisted for hundreds of millions of years.
This is a carefully executed study that integrates multiple datasets and analytical approaches. It offers a valuable and well-documented synthesis, and the findings will be of interest to researchers in vertebrate evolution, developmental biology, and regulatory genomics. However, the core claims are largely confirmatory rather than conceptually novel, and several key questions about mechanism, specificity, and confounding remain unresolved. These limitations affect the strength of the manuscript's central conclusion. While this work is likely to be a useful contribution to the field, I would characterize it more as incremental.
Major Points
1. The enrichment of ohnologues among marker genes may reflect general features of gene age and function rather than a causal role in innovation
2. The key result (that cell type markers are enriched for ohnologues) recapitulates a well-established pattern. WGD-derived genes, especially transcription factors, are known to be dosage-sensitive and broadly retained. They tend to be older, more highly expressed, and more deeply embedded in regulatory networks than most SSD paralogs. These properties alone make them more likely to be detected as markers. The analysis confirms and refines this pattern in the context of cell type diversity, but it does not provide strong evidence that WGD actively generated novel cell types, rather than preserving and subdividing ancestral programs.
3. The same trend is observed in other tissues, including lung and eye. This further suggests a general principle about ohnologue expression, and somewhat weakens the claim of brain specificity. A more compelling argument would require

controlling for gene expression level, age, and regulatory complexity when comparing WGD and SSD paralogs.
4. The paper does not clearly distinguish between association and mechanism.
5. The strongest mechanistic interpretation comes from the cerebellar nuclei section, where different ohnologue expression patterns correlate with duplicated cell types. However, even there, causal claims are limited. Most analyses evaluate whether ohnologues are markers, or whether both members of a duplicate pair mark different cell type families. These associations are interesting, but they do not directly show that WGD facilitated cell type innovation in a way that could not be achieved by SSD or other regulatory changes.
6. In particular, the subfunctionalization analysis relies on inferred ancestral expression states that are easier to reconstruct when expression is lost rather than gained. This introduces a bias toward detecting subfunctionalization over neofunctionalization. The authors acknowledge this briefly, but the implications are not discussed in depth.
7. Similarly, the finding that many paralog families have a dominant member across species is consistent with dosage selection, but it is not clear how such pairs then support distinct cell type identities. These dynamics are central to the argument but are only partially resolved.
8. Species and batch effects may still confound key comparisons
9. The integration across species is handled thoughtfully, using SAMap and other tools, but variance decomposition shows that batch and species effects dominate over cell type signal in the data. It remains unclear whether the observed divergence of paralog expression across species is fully separable from technical and sampling differences. The claim that similar ohnologues are dominant across species is consistent with shared expression bias rather than cell type-specific redeployment. Some key inferences, including those about subfunctionalization, would benefit from additional controls or replication in other datasets.
Minor Points
1. The distinction between "association" (a gene being a marker) and "contribution" (each paralogue in a pair marking different cell types) is not always clearly stated. These are conceptually different and should be more clearly separated in the text.
2. The definition of orthogroups and how they relate to ohnologue and SSD families is a central methodological issue. It is not always clear whether overlapping categories are treated separately, nor how double-counting is avoided.
3. The role of developmental data is mentioned in several places, but it is not clearly integrated into the argument. Since cell types are defined based on adult and juvenile brains, it is unclear how the developmental origins of cell type families are inferred.
Summary
This is a well-executed and technically sophisticated study. It offers an integrated cross-species perspective on the legacy of whole genome duplication in vertebrate neurogenesis and presents important descriptive data. However, the main claims are primarily confirmatory and rely on associations that could reflect general features of gene function rather than WGD-specific mechanisms. The paper provides a valuable resource and an useful synthesis, but the conceptual advance is modest.

Referee #3

(Remarks to the Author)
This study by Zhu et al. investigates the impact of whole-genome duplications (WGD) on the evolution of the vertebrate brain using systematic comparisons of published single-cell transcriptomic data originating from the brains of four vertebrate species (human, mouse, lizard and lamprey) (three amniotes and one cyclostome).

The authors first define cell-type families that share gene expression signatures (transcription factor codes) common to those vertebrates. By comparing gene expression in these cell-type families to data taken from an outgroup in which WGD did not occur, they report an enrichment of genes retained after WGD (“ohnologues”) among the differentially expressed genes (or marker genes), not seen for genes duplicated by other mechanisms (small scale duplications or “SSD paralogues”).

The authors observe a similar pattern with two different sets of data: cerebellar nuclei in amniotes and a subset of the Human Cell Atlas.

Comparisons within the duplicated gene pairs reveal that paralogues most often diverge from their ancestral role (“subfunctionalization”) across cell types and that their expression follows a principle of dosage selection. These observations concerning neuron-type evolution appear to be novel, although similar observations have been made before at tissue scale using bulk RNA-sequencing.

The presented conclusions are original, offer hints about possible mechanisms underlying cell-type evolution and connect them to large-scale genomic changes. However, the link between WGDs and cell-type evolution remains correlative despite the authors claiming that they “contributed to” (lines 479f) cell-type evolution. This latter characterization is probably too strong.

We would welcome clarifications and new analyses to strengthen the paper, as detailed below:

Major points:

1. Ohnologue classification: because all the findings of this study build upon the classification of duplicated genes as ohnologues or other paralogues, the applied approach should be better justified.

a. Ohnologues are, in the ms, inferred across all vertebrates collectively; yet, the lamprey lineage has undergone a separate WGD and therefore has a more complicated paralogue relationship. Would this affect the analysis and if so, how is it controlled for?
b. An asymmetric gene loss during R2 of WGD in jawed vertebrates is mentioned, resulting in different gene retention rates for the alpha and beta lineages (with higher retention rates in one of them). The different lineages should probably be analysed separately, given that they might have evolved under different selection constraints.
c. Gene age can make establishing the true historical relationship between paralogues difficult; for example, old SSDs may be detected as WGDs (see https://academic.oup.com/gbe/article/15/10/evad174/7287110). Was this examined and controlled for in the paralogue classification? If so, how? If no, it probably should.
d. The authors state: “To assess the robustness of our ohnologue predictions, we compared our results to the Ohnologs v2 database (http://ohnologs.curie.fr), finding that 80% of human and 66% of mouse ohnologues in our dataset were also present in the database.” (lines 595f)
Yet, the proportion of the ohnologues listed in the Ohnologs v2 database that were identified in the authors’ dataset is not stated. These numbers should be given.
e. The authors could consider other methods to classify gene duplications and compare them to those presented in the ms (e.g.
https://github.com/qiao-xin/DupGen_finder, https://genomebiology.biomedcentral.com/articles/10.1186/s13059-019-1650-2)?

2. Paralogue contribution to cell type evolution:
f. The authors write that “a single-cell dataset from amphioxus at late neurula stage, filtered to retain only CNS cells, was used as a ‘control’ for analysing relationships between extensive SSD paralogues and DEGs” (lines 171f). Could the fact that the control dataset originates from an early developmental stage (unlike the other datasets) affect this analysis? It is conceivable, if we assume that retained paralogues are more likely to be TFs or regulators, and therefore that they are more highly expressed in developmental datasets, that these genes among the markers will be over-represented? If so, this bias should be corrected.
g. We did not see mention or discussion of the fact that, in the paralogue-pair analysis, the patterns differ between lamprey and jawed vertebrates at both cell-type and -family levels (Fig. 2f and Extended Data Fig. 3i). Could this be a result of the fact that the inference about ohnology is not appropriate for lamprey (see above)?

3. Inference of sub- vs. neofunctionalization:
h. Inferences about sub- vs. neo-functionalization after WGD seem to be very sensitive to the details of the data analysis and on outgroup selection (discussed in https://www.nature.com/articles/s41588-018-0162-4 and https://www.nature.com/articles/s41588-018-0163-3). In this study, no outgroup was used to infer ancestral expression states. Instead, the expression of genes was compared between species, and the ancestral state inferred if a majority of species (e.g. 3 out of 4 vertebrates) showed expression (line 744f). Could this choice affect the observed result of subfunctionalization, if so, in what ways? The missing outgroup issue should be remedied, if possible.
i. High-copy number SSDs were excluded without explanation (lines 728f & 757f). This decision should be explained and justified, and a more detailed definition of SSD should be provided.

Minor points:

1. Mismatch between parameters stated in methods and code:
There are several mismatches between the parameters found in the code provided by the authors and the methods section of the paper.
2. SAMap
Methods (line 605) state: “We then performed cross-species mapping using the SAMap run function with 5 iterations, edge weight calculated and updated by Pearson correlation (hom_edge_mode = "pearson"), and 20 cross-species edges per cell (crossK = 20). Mutual nearest neighbourhoods were independently calculated between each pair of species (pairwise=True).”
However, the corresponding code
(https://github.com/DiracZhu1998/WGD2celltype_evolution/blob/main/0.atlas/6.SAMap/SAMap_run_vertebrate_neurons.py) suggests that the authors used “crossK = 30”.
3. Seurat marker calculation
Methods (lines 644-646) state the following parameters for Seurat’s FindAllMarkers “min.pct = 0.01, logfc.threshold = 0.58, test.use = "wilcox", only.pos = TRUE”, however the code
(https://github.com/DiracZhu1998/WGD2celltype_evolution/blob/main/3.markers/DEGs_Seurat/FindMarkers.R) only seems to use “test.use = "wilcox", only.pos = T”.
The code also shows that the data were subsampled to 3,000 cells per category prior to the marker calculation. This is currently not described in the methods.

```
sampled_df <- sampled_df %>% group_by(args$label) %>% slice_sample(n = 3000) %>% ungroup()
obj <- subset(obj, cells = as.character(sampled_df$cellname)))
```

4. Analysis questions:
a. Why was SAM used for preprocessing when the counts were normalized again using the scrattch pipeline?
b. The SAM code is missing from the GitHub repository.
c. Iterative clustering: Why were different values chosen for split.size between species? The data were downsampled to

roughly the same numbers of cells; hence the reason why they were treated differently at this step is not clear.
d. Trinarization score: The Trinarization score was calculated differently between analyses (min. % of cells was 10% for whole brain data, 20% for cerebellar nuclei data), detailed in lines 741f. & 794f. Why were different parameters chosen for the different datasets? Please harmonize or justify.

5. Visualization and labeling issues:
a. Figure/Legend label mismatch
The cyclostome-specific hexaploidization is referred to as “2Rcy” in the figure legend (Figure 1) but shown as “2Rcv” in the figures (Figs. 1a, 3a).
b. Figure legend description
EDFig. 10 f,g: The legend describes a dot plot whereas the figure shows a violin plot.
c. Color bar for multi-species dot plots
Multi-species dot plots lack a color bar (Figs. 1c, 4g, EDFigs. 1a-d, 2d, 5e, 7e) that encodes strength of expression for the different species or cell type families.
d. Extended Data Fig. 3a-f: it is hard to distinguish the datasets or gene sets shown in the panels. Horizontal bars labeling a-b as human eye+lung data, c-d as TF, e-f as TF targets might improve readability.
e. Fig. 3a is not easy to understand, e.g. the grey and white boxes are not explained in the legend.

(Remarks on code availability)
The code and data provided are a usable resource for the community. We have not installed and run the code, but the repository includes configuration files for conda environments and the README files for most of the analyses described in the paper.
As mentioned above some parameters described in the methods do not match the parameters found in the code. The authors should therefore check the consistency of the methods and code.

Referee #4

(Remarks to the Author)
I co-reviewed this manuscript with one of the reviewers who provided the listed reports.

Referee #5

(Remarks to the Author)
I co-reviewed this manuscript with one of the reviewers who provided the listed reports.

Version 2:

Reviewer comments:

Referee #1

(Remarks to the Author)
The revised manuscript includes expanded analyses and clarifications, and some of the claims have been moderated, as reflected in the revised title.

The authors refine their definitions of ohnologue and SSD paralogue orthogroups, separate alpha and beta ohnologues, benchmark their gene sets against external databases, and perform additional robustness checks, including analyses with and without lamprey. These additions improve the technical rigor of the study.

Upon revisiting the manuscript, I find that most of the principal observations extend well-established principles of gene duplication into a single cell comparative framework rather than introducing new conceptual advances.

The enrichment of ohnologues among vertebrate brain cell type markers, the widespread asymmetric expression of paralogues, and the prevalence of subfunctionalization across cell types are broadly consistent with established principles for WGDs. What is new here is the resolution and comparative scope, with these patterns being shown across multiple vertebrate species at single cell resolution.

The enrichment of WGD derived genes in conserved vertebrate cell type families relative to amphioxus represents the strongest advance. Overall, the novelty of the study lies more in its technical integration and comparative breadth than new ideas on how new cell types evolve.

The authors now incorporate an adult amphioxus brain dataset as the outgroup against which vertebrate patterns are interpreted. This is an important addition. However, it requires quality control comparisons (e.g., sequencing depth, gene detection rates, etc) demonstrating that the amphioxus dataset is technically comparable to the vertebrate datasets.

This is particularly important because amphioxus effectively serves as the reference baseline for the enrichment analyses. If differences in marker detection or cell type resolution reflect dataset sensitivity rather than biology, the apparent excess of vertebrate ohnologues could be influenced by technical artifacts.

A limitation of the manuscript is that it does not firmly link specific gene duplication events to specific cell type divergences. The authors show that ohnologues are enriched among marker genes and frequently diverge in expression, and they provide illustrative case studies (e.g., macroglial programs) suggesting how paralogue partitioning may have accompanied cell type diversification. However, they do not demonstrate that particular duplication events preceded and enabled defined neural cell type splits in a temporally resolved manner.

In this sense, the study supports the view that WGDs shaped the gene landscape of vertebrate brains, but it does not provide the strongest possible evidence that they were involved in the generation of new cell types. The use of “contributed” in the title feels conceptually ambiguous.

Two concerns from my initial assessment remain. First, the framing of the analysis as a comparison between WGD and SSD remains narrow. By focusing on the duplication mechanism, the manuscript does not attempt to evaluate alternative sources of increased regulatory complexity, such as expansion of cis-regulatory elements or rewiring of GRNs. Second, if WGDs were sufficient to drive the emergence of new cell type families, one might expect lineage specific WGDs to have produced clear examples of novel, conserved cell type repertoires.

The manuscript provides a well-executed comparative analysis, and it will be of interest to the field. Yet, I found the framing of causality throughout the manuscript to be ambiguous. Causality is continuously implied and then walked back, resulting in a lack of clarity regarding the strength of the conclusions.

(Remarks on code availability)

Referee #2

(Remarks to the Author)
I thank the authors for their thoughtful responses to my remarks. While I am a little ambivalent, I think they have got about as far as one can in what is fundamentally a very hard model to assess (temporal confounding, not open to causal experiments, etc). I'm hard pressed to think of real angles unaddressed that would not require dramatically more data. That data may come with time but in the meantime, this is a compelling presentation of a model that one can accept as standard.

That said, while the fundamental claims within the paper are compelling, they really don't support the modest overstatement inherent in the title.

Referee #3

(Remarks to the Author)
We thank the authors for their revisions.

Strengths:
the new data collected from adult amphioxus and additional analyses (alpha/beta WGD lineages, SSD gene age) strengthen the authors’ finding that ohnologs, especially from first WGD, are associated with increased cell type complexity and evolution in vertebrates

the sc/snRNAseq data from adult amphioxus seem to be of high quality and will likely be useful to others interested in cell-type evolution. The authors do not comment on the number of sequenced cells in comparison to total cell numbers present in amphioxus, but the 5568 sequenced cells (3217 neurons + 2351 non-neurons) compared to a total of ca. 20,000 neurons [PMID: 22676056] seem to be a sufficiently high fraction (~16%). It remains not totally clear to us, however, whether the different clustering procedure might affect the conclusions drawn about cell type relationships (see below).

Causal experiments are admittedly hard. The authors focus on the macroglia/astrocyte-oligodendrocyte cell type relationship and investigate a SoxE knockout in Amphioxus, making a reasonably convincing case for a broader and less essential expression of SoxE in Amphioxus glia compared to knockouts of Sox8/9/10 in vertebrates. This supports their hypothesis of a subsequent individualisation of sister cell types (astrocytes and oligodendrocytes) in vertebrates.

We appreciate the detailed comparison of ohnologue detection methods (Doubletrouble, DupGen_finder, and Ohnologs-v2.0) added by the authors.

Remaining questions:

The manuscript contains duplicated figures with different data for which the discrepancy is not adequately explained:
What is the difference between SFig. 6B and EDFig. 4C;
bw EDFig 4B and SFig. 6A;
bw Fig 2B and SFig 6C?

The OligB expression is barely visible in the transverse section of Amphioxus T1 (Fig 3d and EDFig. 6f)? The authors should consider changing the colors (and to be colorblind-friendly) and/or image intensity.

Methods question: clustering analysis for Amphioxus data differs from vertebrate datasets, resulting in a much lower number of clusters for Amphioxus (23 vs. 141-241 clusters). The marker genes are then compared, showing broader expression in Amphioxus leading to the conclusion that it represents a lack of sister cell type diversification pre WGD. Is this conclusion affected by (a result of) the clustering procedure?

Minor comments:
Is there a typo in the EDFig 6h figure legend? Should it say “N4 stage” instead of “T1 stage”?
Gene names for (Tbr1, Tbx21 and Eomes) in lamprey Fig. 4d. Why do lamprey gene IDs have a “MSTRG” or “PMZ” prefix?

The authors should consider softening the claim “Fig. 4 | Divergence of paralogues drive cell-type evolution.” (line 450) perhaps to “... are associated with cell-type evolution”?

Fig. 4g has species annotations that differ from the others, e.g. “Hsap” vs “H. sap.” elsewhere.

Referee #4

(Remarks to the Author)
I co-reviewed this manuscript with one of the reviewers who provided the listed reports.

Referee #5

(Remarks to the Author)
I co-reviewed this manuscript with one of the reviewers who provided the listed reports.

Version 3:

Reviewer comments:

Referee #1

(Remarks to the Author)
The authors have made an appropriate effort to address the main points raised in the previous round of review, clarifying aspects of the analysis and moderating the strength of claims that were previously overstated.

While some of the broader conceptual concerns (regarding causality and the extent of conclusions) remain partially resolved, I appreciate that these issues have been acknowledged and the manuscript revised accordingly.

The study provides a technically solid and comprehensive comparative analysis that will be of interest to the field, even if its conceptual advances are more incremental than initially framed. In light of the revisions and the overall strength of the dataset, I have no further objections.

Referee #3

(Remarks to the Author)

Referee #4

(Remarks to the Author)
I co-reviewed this manuscript with one of the reviewers who provided the listed reports.

Referee #5

(Remarks to the Author)
I co-reviewed this manuscript with one of the reviewers who provided the listed reports.

Open Access This Peer Review File is licensed under a Creative Commons Attribution 4.0 International License, which permits use, sharing, adaptation, distribution and reproduction in any medium or format, as long as you give appropriate credit to the original author(s) and the source, provide a link to the Creative Commons license, and indicate if changes were made.
In cases where reviewers are anonymous, credit should be given to 'Anonymous Referee' and the source.
The images or other third party material in this Peer Review File are included in the article's Creative Commons license, unless indicated otherwise in a credit line to the material. If material is not included in the article's Creative Commons license and your intended use is not permitted by statutory regulation or exceeds the permitted use, you will need to obtain permission directly from the copyright holder.
To view a copy of this license, visit https://creativecommons.org/licenses/by/4.0/

Response to Referees

Zhu et al

Referees' original comments are reproduced below, with our response in blue text.

Overall Response

We thank the referees for their constructive and insightful comments, which have been extremely helpful in our efforts to improve the manuscript. We were pleased to see the referees recognised the importance of this study and that it provides a valuable framework to understand vertebrate brain cell-type families, their homology across vertebrates, and the roles of gene duplication in cell-type evolution. At the same time, the referees also raised several concerns, including regarding our outgroup datasets, the potential effects of gene age, changes at additional levels to gene duplication (sequence, regulation, and motif) and their contribution to cell-type evolution, and WGD causality in cell-type evolution.

In the revised manuscript, we have carefully addressed each of the points raised by the referees. This has included developing new adult amphioxus "brain" and neural tube single-cell datasets, proposing new evolutionary models, and adding new computational analyses and experiments (including data and experiments on an amphioxus mutant line) to test the causal of WGD. Detailed point-by-point responses to each comment by each referee are provided further on in this document. Before this, we provide a summary of the major revisions we have for the resubmission:

Major revisions

Major revision 1. As the referees noted, our use of amphioxus embryo scRNA data as an outgroup to vertebrate adult brain was limiting. We have hence generated adult amphioxus brain scRNA data to use as the appropriate outgroup. Using these new data we re-investigated vertebrate cell-type families and their relationships to cell types in amphioxus. We found no clear 1-to-1 cell type homology but with relationships definable at higher hierarchical levels. These analyses are in the new Fig. 1D (reproduced below) and Extended Data Fig. 3. This lack of 1-to-1 relationships led us to question whether our interpretation might be being skewed by our use of vertebrate whole brain data, which can be dominated by the cortex. We therefore also explored cell-type relationships between the amphioxus and vertebrate hypothalamus, considered to be a very ancient and relatively stable part of the brain, finding the same patterns of relationship (Supp. Text, Supp. Figs. 3-5).
These additions remove the original limitations from comparing developmental to adult data. They also better reveal vertebrate cell-types and families that are vertebrate innovations, and further work on these is part of our response, detailed in Major Revision point 3 below.

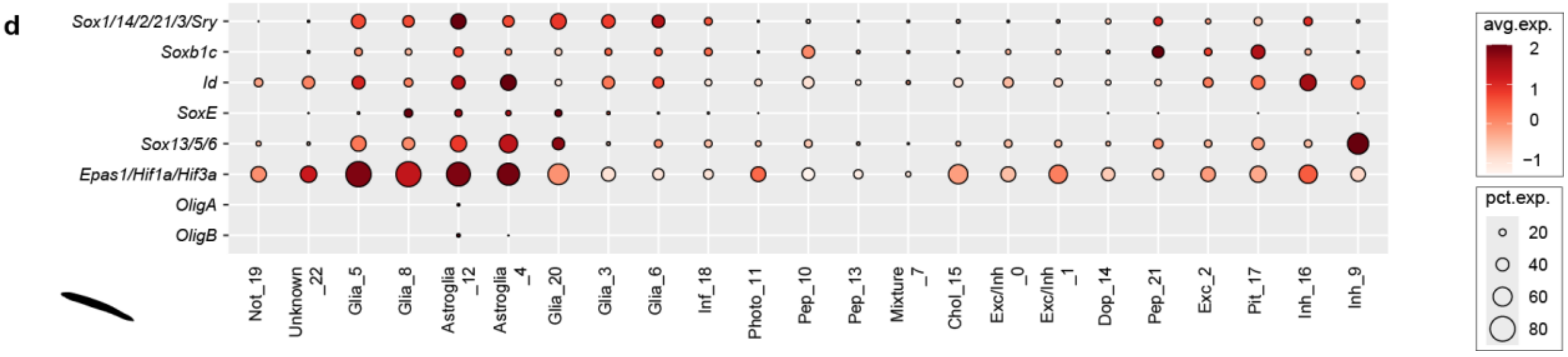

Figure 1d: Expression of conserved key TFs of vertebrate astrocytes in adult amphioxus brain cell types. A complete dot plot for all vertebrate cell-type families is shown in Extended Data Fig. 3.

<u>Major revision 2.</u> We have revisited ohnologue identification and have incorporated analysis of gene duplication age. As suggested by Referee #3, we ran a detailed comparison of ohnologue identification methods (including Doubletrouble, DupGen_finder, and Ohnologs-v2.0), and we generated a new version of ohnologue sets based on analysis of more vertebrate genomes. The Referees also rightly pointed out that gene duplication age may be important for both WGDs and SSDs. We have hence classified ohnologues into 'alpha' and 'beta' sets, plus estimated the duplication time of SSD paralogues. This allowed us to ask how gene duplication timing interacts with the likelihood that duplicated genes are cell type markers. We found that our conclusions still hold when considering this. The outcomes are in Figure 2a-c (reproduced below), Extended Data Figures 4 and 5, and Supplementary Figures 6 and 7.

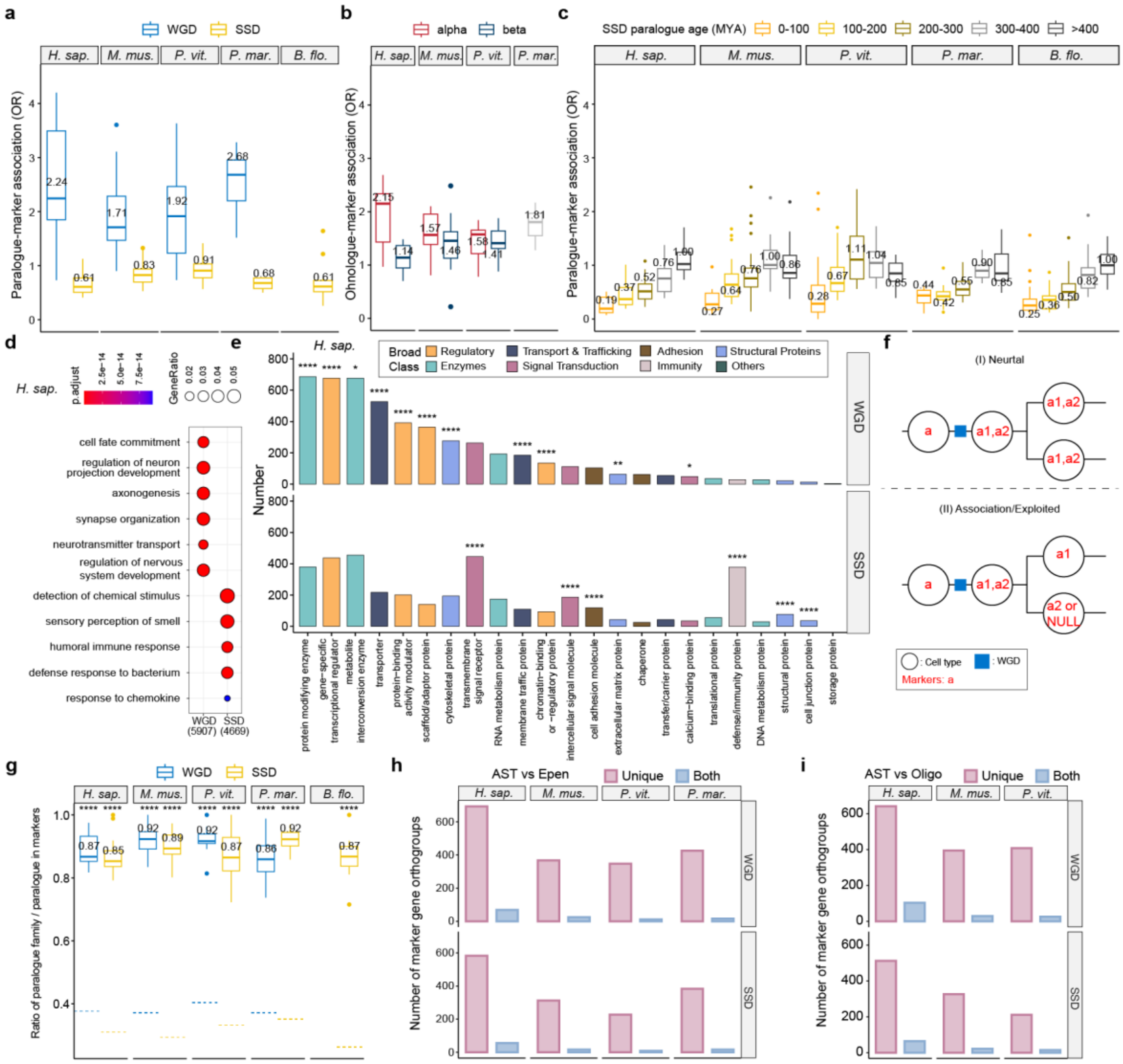

*Figure 2. **Ohnologues contributed more to cell-type evolution than SSD paralogues.** The key changes to this are new results with consideration of ohnologues lineages (**b**) and gene age of SSD paralogues (**c**). In addition, we include analysis to sister cell types to understand how paralogues differentially exploited in (**h** and **i**), showing the number of paralogue families containing markers between two pairs of sister cell types AST vs. Epen (astrocytes vs. ependymal cells for **h**) and AST vs. Oligo (astrocytes vs. oligodendrocytes for **i**). 'Unique' stands for paralogue families only used by one of the sister cell types. 'Both' represents paralogue families used by both sister cell types but different copies were differentially used as markers between sister cell types. For the full Figure legend please see the main manuscript.*

<u>Major revision 3.</u> The Referees raised the question of WGD causality in cell-type evolution, and we have undertaken a series of experiments and analyses to further explore this. It is not possible to go back in time and directly observe and test ancient events and processes, and hence 'prove' ancient causation. However, it is possible to take a formal scientific approach and falsify such hypotheses if they make testable predictions, and this is the route we have followed.

First, we establish models to help understand whether cell-type complexity evolved before or after WGD (Figure 3a, reproduced below): in brief, if cell-type complexity can be shown to have evolved before WGD, it would disprove a causative role for WGD. Our new amphioxus data allow us to explore this, and the data do not support the 'cell-type first' model. We have then exploited the new amphioxus data to experimentally explore a test case in more detail. We chose macroglia because in vertebrates there are robust data on key TF functions in their specification, allowing us to predict what the ancestral state should be. We built a cross-chordate tree of glial cells and found that amphioxus 'primitive' glial cells co-express the key TFs that separate sister cell types in the macroglia of vertebrates. We confirmed TF gene expression in amphioxus using HCR in situ hybridisation and generated and analysed an amphioxus mutant of one of the key TFs, *SoxE*. These results are in the new Figure 3 (reproduced below), Extended Data Fig. 6, and Supplementary Fig. 9.

The revisions here allow us to deduce ancestral gene expression patterns and mechanisms, and to set these against the actual state in the outgroup to test if outcomes are consistent with (i.e. do not falsify) a causative role for WGD. Our test case shows that gene duplicates were required for innovation in the vertebrate lineage. The significant challenge involved in generating amphioxus mutants means we cannot show this for all key TFs. However, we believe they provide hard evidence for a causative role for ohnologues in a brain cell type innovation and, coupled with our previous analyses of the cerebellar nuclei (see Figure 6), show how important these specific WGDs were for vertebrate evolution.

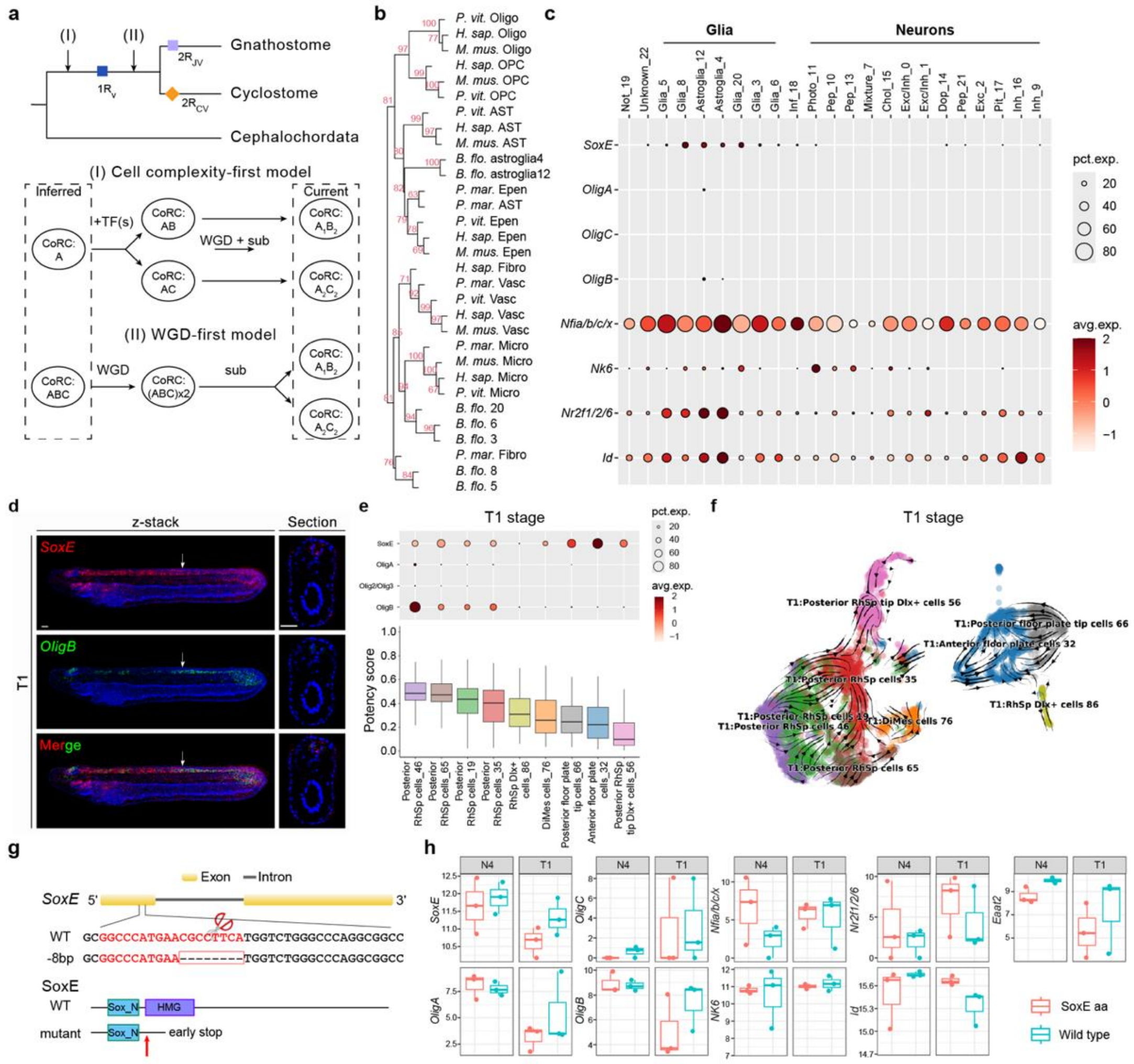

__Figure 3. Macroglia evolution.__ This is a new figure. A brief description is provided here; please see the main manuscript for the full legend. ***a,*** *Models linking WGD to sister cell type evolution.* ***b***, *Tree based on TF expression specificity distances among non-neuronal cell types across five chordates.* ***c***, *Key TF expression separating astrocyte and oligodendrocyte in the adult amphioxus brain.* ***d***, *HCR-FISH of SoxE and OligB at the T1 stage.* ***e***,***f***, *Multipotency scores and RNA velocity of amphioxus CNS glia at T1. A dot plot of SoxE and Olig is shown at upper panel.* ***g***, *SoxE gene models, deletion strategy, and protein domains.* ***h***, *DESeq2-normalized expression of key TFs and Eaat2 in SoxE mutants and wild type.*

<u>Major revision 4.</u> The Referees raised the point that the manuscript was overfocused on WGD as the primary factor in cell-type evolution, to the exclusion of other potential contributors/explanations. We agree that we had overemphasised this by omitting mention of other potential processes and have adapted the revised text accordingly, although we argue that properly investigating any one of these with the depth and rigour we have tried to apply here to gene duplication is itself a substantial new study.

The Referee's comments did, however, prompt us to think about these different levels of change in the context of gene duplication, which we think is an appropriate extension of our current study. To

investigate this, we have explored other differences between WGD and SSD, including coding sequences, protein-protein interactions, TF target genes, and regulatory relationships (motifs). The new data are quite granular so while we describe them in the results section the details are included in the Supplementary Materials (Supplementary Text and Supplementary Figures 18-20). Alongside this we have revised the discussion to present a more balanced view of gene duplication in the context of other types of genetic change and its potential importance.

Major revision 5. A question that emerged from our more detailed exploration of amphioxus is the relevance of brain regionalisation, that is of cell-types found at different locations along the AP axis. We were able to utilise published adult amphioxus CNS bulk RNA-seq data alongside our single cell analyses to further explore this. This allowed us to identify 'regionalisation' TFs persisting in adults. We compared regional-identity TFs between vertebrates and amphioxus and found that some are shared across chordates, while others are vertebrate innovations. This outcome has also been added to the revision.

Alongside these revisions we have made substantial changes to the text reflecting other comments and have tried to make figures 'smarter' and more intuitive. Below we now provide a more detailed point by point response to each Referees comment.

Point-by-point response:

Referee #1 (Remarks to the Author):

This is an extensive study by Zhu and colleagues where the authors take advantage of single-cell RNA-seq datasets from multiple vertebrate species to explore the contribution of whole genome duplications to brain cell type evolution.

The scale of the analysis performed by the authors is impressive, and the use of comparative single-cell genomics to address a longstanding question in vertebrate evolution is a strength of the work. The study focuses on four main species: human, mouse, lizard, and lamprey- with some amphioxus data which is used as control. The central hypothesis tested is that WGDs, more than small-scale duplications (SSDs), contributed to the diversification of vertebrate brain cell types.

The results begin with a phylogenetic mapping of major neural cell type families, showing that most major vertebrate brain cell type families are already present before the cyclostome-gnathostome split. They note that some families, including oligodendrocytes and cerebellar granule cells, are more recent, emerging within jawed vertebrates and being absent from lampreys.

The delineation of these families is in itself an interesting contribution, providing a nice framework for comparing brain cell type diversity across vertebrates. However, here I missed a better analysis of the control group (the amphioxus) as an examination of the gene families that already existed prior to the genome duplications. While the authors state that "most major neural cell type families predate the cyclostome-gnathostome split," they do not present data on the presence or absence of these same cell type families prior to 1R/2R.

Response 1: We appreciated Referee #1's generally positive comments here. With respect to the specific question of the 'control group' (amphioxus) and cell-type families predating 1R/2R, this requires appropriate data from the outgroup, adult amphioxus. At the time of the 1st submission we only had data from one of our previous studies[1], which was embryonic, and we agree that this did limit our original analyses. We have therefore generated and analysed single-cell RNA data from amphioxus adult brain, and have used this to address the referee's comments. We annotated 21/23 amphioxus brain cell clusters, including dopaminergic neurons, excitatory neurons, inhibitory neurons, infundibular organ cells, peptidergic neurons, pituitary gland cells, astroglia, etc (Supp. Table S2-Bflo, Supplementary Fig. 1 and 2e). We then compared vertebrate brain cell-type families to amphioxus using SAMap, and through the comparison of vertebrate conserved key TFs, to understand the origin of vertebrate cell-type families. We found similarities between amphioxus astroglia and vertebrate macroglia (including ependymal cells, astrocytes, and oligodendrocytes) and these are explored more deeply with respect to mechanism (see below). Amphioxus neurons generally map to diverse neuronal cell-type families found in vertebrates (new Extended Data Fig. 2c,d). However, amphioxus cell types do not express conserved TFs defining vertebrate cell-type families in a concise and exclusive way (new Fig. 1d and Extended Data Fig. 3), suggesting vertebrate cell-type families we defined are vertebrate innovations.

One concern we considered here was whether our amphioxus-vertebrate neuron cell-type comparison was being impacted by the dominance of cortical cells in vertebrate datasets, as this part of the brain is known to be hugely expanded in the latter. We therefore also explored a more evolutionary conserved region, the hypothalamus, and still found no clear 1-to-1 relationships between amphioxus and mouse. The Referee's specific question on whether cell-type families predate WGDs is also related to concerns over mechanism raised by Referee #2, and we provide a full response there (see Response 11 for Referee #2) including new analyses and experiments on macroglia evolution.

The next analysis quantifies paralog retention among cell type markers, revealing that ohnologues are overrepresented compared to SSD paralogs across the majority of cell type families. The authors found that this enrichment is especially pronounced for transcription factors and other developmental regulators.

Here I missed more detail on the identification of ohnologues versus SSD paralogues (total numbers of genes in each group?) and whether they were matched for set size in the enrichment analyses. Without this information, it was difficult for me to rule out the possibility that the observed enrichment simply reflects differences in baseline abundance between the groups rather than a true functional bias (Figure 1D shows equivalent numbers of genes for WGD and SSD under transcriptional regulators – is this correct?).

Response 2: The total number of genes in each group was added in lines 205-207 of the revision. Because the GO analysis used only protein-coding genes *expressed* in the brain as the background, the numbers of WGD/SSD paralogues (including not expressed) are slightly higher than those used in the enrichment analyses (Fig. 2d and Extended Data Fig. 5a). For the referee's specific question regarding 'Figure 1D' in the original manuscript, we assume they mean original Figure 2D (Fig. 2e in the revised version) here as this is the panel relevant to their question. We generated a new version of ohnologues (based on more vertebrates compared to our original version) with higher overlaps compared to Ohnologs-v2.0 and to Marlétaz and colleagues' results[5] (we now provide detailed method comparisons, including at # Referee 3's suggestion evaluation of some other ohnologue detection approaches, in the Supplementary Text). In this revised ohnologue set, the number of ohnologues classified as transcriptional regulators was higher than those for SSD paralogues (Fig. 2e). Ohnologues were significantly enriched as transcriptional regulators, while SSD paralogues were not (as we saw before). Additionally, SSD paralogues are less likely to be markers (Fig. 2a). These results highlight the importance of WGD.

Next, the authors expression divergence between paralogues across cell types and find that subfunctionalization is the most common event for both ohnologues and SSD paralogs. Clear cases of neofunctionalization, where a paralog acquires a novel expression domain, are comparatively rare.They also observe that over two-thirds of duplicate families have one copy consistently more highly expressed than the other across cell types. This pattern is interpreted as evidence for dosage selection under the gene balance hypothesis. The authors also explore dosage sensitivity, reporting that ohnologues tend to be retained and expressed in a dosage-balanced fashion more often than SSD paralogues.

Finally, the authors examine cell type origin timing relative to gene duplication events, finding that cell type families that arose after WGDs show preferential incorporation of ohnologues, and that paralogue pairs can be linked to novel cell type identities. These patterns are interpreted as evidence that WGD provided a genomic substrate for both the emergence of entirely new cell types and the diversification of existing ones.

Response 3: We thank # Referee 1 for the summary here. The conclusions summarised above hold under revised ohnologue sets and a stricter definition of WGD/SSD orthogroups (lines 373-376).

While these observations are intriguing, I found that the overall framing of the paper strongly favors WGD as the primary driver of new cell type emergence without adequately testing alternative explanations. One competing hypothesis is that increases in regulatory complexity, such as the elaboration of higher-order regulatory network configurations, were the dominant force in generating new cell types. Without analyses that explicitly test for changes in network rewiring, the conclusions about WGD's causal role are not fully supported.

The study's framing of WGD versus SSD as the key drivers of new cell types risks overlooking other, equally important mechanisms that generate regulatory complexity.

Response 4: This is an important point, and we thank Referee #1 for raising it. It is related to additional points raised by another Referee below (Referee #2 Major point 5), and we address them together here. First, we have not been clear enough in our language, in that our analysis of WGDs in cell-type evolution does not preclude that other mechanisms might also have roles. We agree that there are many other possible mechanisms for altering a cell type's core regulatory complex (CoRC), including the emergence of and changes to binding sites, mutations in transcription factors enabling them to bind pre-existing sites, the evolution of new transcription factors/cofactors, and novel protein–protein interactions. We have amended the text in several places (e.g., lines 679-682 in the discussion) to make it clear that WGDs role is not exclusive of these.

As mentioned in our response above under 'Major Points' we have not addressed all of these in addition to gene duplication here as to do these robustly each be another major piece of work. However the Referee's comment prompted us to think more about how these additional sorts of change might interact with gene duplication, which we do think is within scope and something we should have done before. To explore this further, we have performed new analyses in human and mouse on coding sequences, gene regulatory relationships, and protein–protein interactions, to test how WGD and SSD interact with these (Supp. Text). We found ohnologue pairs are more constrained regarding coding sequences (Supp. Fig. 18) and TF target genes (Supp. Fig. 20), and that ohnologue proteins tend to interact more with other proteins. We therefore think WGD provided stable redundancy in core regulatory proteins (TFs, co-factors, and signalling systems) because they are more likely to be dosage sensitive. This created a buffer that allowed gradual subfunctionalization of ohnologs across tissues/cell types. In contrast, SSDs are *less likely* to persist long enough under constraint to contribute to stable, cell-type–specific regulation in the same way. We have updated the text (lines 425-430) to incorporate this in a more holistic view that considers these other factors, based on above new analyses.

The reliance on adult brain transcriptomic snapshots further limits the interpretation of these results. Cell type molecular profiles in the adult reflect the outcome of developmental programs, many of which involve the sequential activation and repression of paralogs over time. By ignoring the temporal dimension of cell type specification, the study risks missing instances where duplicates have critical developmental roles but little or no expression in the adult. Paralog subfunctionalization often occurs along developmental time axes rather than in the final adult state, and without developmental single-cell data, this dimension of WGD influence remains unexplored.

I appreciate the challenge the authors face in light of this problem - as following developmental trajectories and mapping cell type specific GRNs involved is a big task. It would require combining data from multiple stages and species to track how paralogues are used over time. Still, without this developmental view, it is hard to fully answer the study's main question.
Response 5: At one level we agree with the Referee here, since novel developmental lineages must have emerged when novel cell types evolved. However, we already know the relationship between developmental trajectories and cell types can be complex: near-identical cells can develop from different trajectories, and homologous cell lineages can produce different cell types in different body parts[6]. While focusing on adult cell types will not pick up whether paralogues are being used in systematically different ways during development, we do not think this undermines the finding that they are being used in different ways in adult cell types, as what we are largely seeing here is the TFs or CoRCs for terminal differentiation, for an individualised cell type with specific function and self-sustained regulatory programme.

A further concern is that (if I understand this correctly) the study pools all ohnologues into a single category, implicitly treating the two rounds of vertebrate WGD (1R and 2R) as equivalent. 1R predates aligns more plausibly with the origin of conserved vertebrate brain cell type families, whereas 2R likely postdates most of these and may have contributed primarily to gnathostome-specific innovations such as oligodendrocytes or certain cerebellar cell types. Without distinguishing between 1R- and 2R-derived paralogues, the claim that "WGD drove vertebrate brain cell type evolution" risks overstating the role of the second round of duplication.
Response 6: All three Referees pointed this or similar aspects out and it is an important point. We can't separate cyclostome 1R/2R ohnologues due to extensive gene losses in cyclostomes[5]. We instead do it for gnathostomes, as shown below. Simakov and Marlétaz[7] found extensive asymmetrical loss at 2R such that in jawed vertebrates genes from species 'alpha' were more likely to be retained than those in species 'beta'. Therefore, the second WGD in jawed vertebrates was less important than the first one in early vertebrates in terms of the number of retained genes. This was also pointed out by Referee #3 (their Major point 1). We show a hypothetical example here for better

demonstration: a gene duplicated in the 1st WGD to give rise to copies 1 and 2. Speciation events then occurred, giving rise to species 'alpha' and 'beta'. Then the second WGD was by inter-specific hybridisation between 'alpha' and 'beta', resulting in alpha 1, alpha 2, beta 1, and beta 2 s. Any ohnologues between 1 and 2 within the same species were derived from 1R, while any ohnologues between 'alpha' and 'beta' duplicates were derived from 2R. Therefore, separating 1R/2R into pooled ohnologues is meaningless (as pooling breaks the pair relationships in ohnologues and leads to the same gene set).

To avoid complexity here we separated ohnologues in jawed vertebrates into 'alpha' and 'beta' categories (as also suggested by Referee #3 their major point 1b) based on orthology assignments from Marlétaz and colleagues[5], and found that our conclusions regarding the association between ohnologues and markers remain consistent. Interestingly, the enrichment between ohnologues and markers is stronger for 'alpha' than 'beta' ohnologues (especially for human, Fig. 2b). We also compared 'alpha' and 'beta' ohnologues on GO enrichment and found 'alpha' enriched more important terms in development (new Extended Data Fig. 5b). These provide additional evidence suggesting 1R is more important in jawed vertebrates, as is 'alpha' (when compared to 'beta') and we have updated the manuscript to include this new analysis and its outcome (new Fig. 2).

(Incidentally, the discussion falls short in connecting the timing of genomic events to the evolutionary history of the cell type families. By not explicitly considering whether particular cell type families arose before, between, or after the two WGDs, the narrative leaves unclear how the sequence of duplications and other genomic changes maps onto the appearance of new cellular identities.)

Response 7: We agreed with this and in addition have also performed additional analyses relevant to this comment in response to comments from the other Referees (please see Response 1 for Referee #1 and Response 11 for Referee #2). We have rewritten the discussion accordingly based on these new analyses. It is worth noting that cell-type homology is a hierarchical concept, for example astrocytes are a vertebrate innovation however, at a higher hierarchical level (for example Chordata), we can find homologous glia (as ancestor cell types of vertebrate astrocytes, ependymal cells, and oligodendrocytes). A more detailed exploration of this is part of our investigation of mechanism, and is more fully detailed in Response 11 below.

Furthermore, if WGDs inherently drive the emergence of new cell types, one might expect lineage-specific WGDs in other clades (such as the cyclostome hexaploidization, the teleost duplication, or the salmonid WGD) to also give rise to novel cell type families. This seems to be implied by the title/abstract. However, the manuscript does not address this prediction, and the absence of such evidence could suggest that WGD's effect is context-dependent, with the first vertebrate WGD

playing a unique role in early neural diversification, and later WGDs acting mainly to fine-tune or diversify existing cell types within a lineage.

Response 8: We are sorry we did not make this sufficiently clear, as we had not meant to imply that WGD is *always* linked to cell-type evolution: there are many examples of animal WGDs that show no evidence of being linked to such evolutionary changes (or phenotypic complexity). The emergence of novel cell types, like other major adaptations, must also be related to selection, that is the environment at the time. In other words, WGD contribution to cell-type evolution will be context-dependent, as we would expect for any form of mutation affecting the phenotype. The key point we seek to test is whether early vertebrate WGD *did* contribute in this way. We have updated the manuscript (lines 673-683 in the Discussion and including in the abstract) to reflect this.

We think the idea that the Referee's idea that '*the first vertebrate WGD playing a unique role in early neural diversification, and later WGDs acting mainly to fine-tune or diversify existing cell types within a lineage*' is very interesting. As discussed just above (Response 6), the second WGD in jawed vertebrates is considered to be less important than the first WGD in terms of paralogue retention, but that does not mean it had no role. For example, *Sox10* originates from the 'beta' and *Sox8* from the 'alpha'; *Sox10* plays a more critical role in oligodendrocyte development than *Sox8*, as demonstrated by knockout and induction experiments[8,9]. We have discussed this more clearly (lines 689-693 in the Discussion) as it follows naturally from the experimental work on *SoxE* genes that we have added to the revision.

Asking whether further WGDs had similar impacts, especially the 3R duplication in teleost fish, would be interesting to do, but it requires cell-type atlases from the relevant taxa such as fishes with and without these additional WGDs, which have yet to be generated. This would also take the focus away from the core question of early vertebrate evolution We hence think this would be well beyond the scope of this study, and we do not address this with new work in the revision.

Referee #2 (Remarks to the Author):

Shimeld et al. present a comparative analysis of single-cell transcriptomic data from four vertebrate brains, with additional outgroup data, to examine the contribution of whole genome duplication (WGD) to the evolution of neural cell types. The authors define conserved cell type families across species, identify enriched transcription factors, and find that ohnologues are overrepresented among cell type markers. They interpret this as evidence that WGD played a foundational role in the diversification of brain cell types, and that its impact has persisted for hundreds of millions of years. This is a carefully executed study that integrates multiple datasets and analytical approaches. It offers a valuable and well-documented synthesis, and the findings will be of interest to researchers in vertebrate evolution, developmental biology, and regulatory genomics. However, the core claims are

largely confirmatory rather than conceptually novel, and several key questions about mechanism, specificity, and confounding remain unresolved. These limitations affect the strength of the manuscript's central conclusion. While this work is likely to be a useful contribution to the field, I would characterize it more as incremental.

Response 9: We thank the Referee for both their acknowledgment of the strengths of our work and the considered criticism. We agree that some of the questions we address are not fully novel, having essentially been around in one form or another for many decades. However, previous work has been through case studies of single genes which were inevitably unable to quantify effects, or through bulk transcriptomics which lack the resolution to connect to cell types. They do not answer the questions: these haven't been investigated with the systematic consideration of cell types, cell-type families, and gene families as we have done here.

A few studies have tried to demonstrate cell-type homology across vertebrate/chordate organs, but without consideration of the hierarchical nature of cell homology or the importance of CoRCs they conflate homology with similarity. The key conceptual point here is the levels of homology and how to properly define them in a systematic and comparable way. The 'cell' is a fundamental biological unit, and specialised cell types are a key property of complex multicellular systems. By considering proper levels of homology comparison (cell-type family and cell subtype/clustering) in our research, and by connecting gene duplications with cell-type evolution, we are demonstrating the contribution of duplications to these defined units, not broad correlations with a window in evolutionary history when considerable changes happened (see new Fig. 2f-I and Fig. 3).

We disagree that demonstrating this is confirmatory/incremental. However, we do agree that not fully considering "mechanism" (WGD casual roles) in our original analysis may have contributed to this view, and we have addressed this. We detail this below alongside Referee #2s specific comments: please refer to Response 11 for Referee #2.

Major Points

1. The enrichment of ohnologues among marker genes may reflect general features of gene age and function rather than a causal role in innovation

Response 10: This is an excellent point, and we thank the Referee for raising it. We have addressed duplication categories ('alpha' vs. 'beta') for ohnologues, please refer to Response 6 for Referee #1. For SSD paralogues, we have reanalysed our data using two ways to measure duplication age: 1). information from Ensembl BioMart based on gene trees; 2). The rate of synonymous substitutions (Ks). There are some differences between the gene age distributions identified by these metrics, but they are generally consistent. We then separated SSD paralogues into 5 groups based on duplication time windows estimated by Ks and performed the same analyses as we had done before on all SSDs. We find that our conclusion that SSDs are less likely to be cell type markers etc still hold for SSDs irrespective of species and duplication age and we have incorporated these new data into the revised

manuscript (Fig. 2c and Extended Data Fig. 4c). In general, recent SSDs show more negative associations than ancient SSDs, but the most ancient SSDs are still lower than WGDs.
With respect to gene function, marker TFs contribute to distinct regulatory states, whereas marker effectors contribute to distinct functions. Therefore we think it is reasonable that the enrichment of ohnologues among marker genes reflects their functional importance in cell-type evolution and this is not inconsistent with our findings. However, as Referee #2 raised and we agree, this is still not causation. We demonstrate ohnologues are used differentially by sister cell types in new Fig. 2h and i, suggesting association between ohnologues and cell-type evolution. Exploration of causality is detailed below in the next response (Response 11 to Referee #2).

2. The key result (that cell type markers are enriched for ohnologues) recapitulates a well-established pattern. WGD-derived genes, especially transcription factors, are known to be dosage-sensitive and broadly retained. They tend to be older, more highly expressed, and more deeply embedded in regulatory networks than most SSD paralogs. These properties alone make them more likely to be detected as markers. The analysis confirms and refines this pattern in the context of cell type diversity, but it does not provide strong evidence that WGD actively generated novel cell types, rather than preserving and subdividing ancestral programs.
3. The same trend is observed in other tissues, including lung and eye. This further suggests a general principle about ohnologue expression, and somewhat weakens the claim of brain specificity. A more compelling argument would require controlling for gene expression level, age, and regulatory complexity when comparing WGD and SSD paralogs.
4. The paper does not clearly distinguish between association and mechanism.
5. The strongest mechanistic interpretation comes from the cerebellar nuclei section, where different ohnologue expression patterns correlate with duplicated cell types. However, even there, causal claims are limited. Most analyses evaluate whether ohnologues are markers, or whether both members of a duplicate pair mark different cell type families. These associations are interesting, but they do not directly show that WGD facilitated cell type innovation in a way that could not be achieved by SSD or other regulatory changes.
Response 11: We are dealing with these 4 comments together as they are interrelated. First, with respect to point 3, we had not meant to imply that the brain was special in this regard: Indeed, one of our final discussion points (lines 539-541 of the original manuscript) was suggesting that the same processes would be found for other tissues. We have made sure this is clear in the revised text (lines 617-619 in Discussion). Similarly, with respect to the final part of point 5 and as we address under Response 4 to Referee #1, we did not mean to imply that innovation can *only* be achieved by WGD. Please see this response for how this have been dealt with in the revised manuscript.
The central point in comments 2-5 from Referee #2 is that we do not demonstrate 'mechanism', that is we do not show a causal role for WGD in cell-type evolution. This is in effect a rephrasing of a long-

standing criticism of hypotheses linking WGD to vertebrate evolution, suggesting that rather than being mechanistically important or causative for evolution, WGD is a ‘passenger’, with the patterns we see in ohnologue expression in living taxa having resulted from passive processes like reciprocal subfunctionalization. This criticism has historically been hard to address, as both WGD and the evolution of phenotypic complexity map to the same part of the evolutionary tree. Further, as we say in the summary at the start of this document, it is also not possible to directly test what happened hundreds of millions of years ago. Fossil data has provided some insight[10], but cannot inform on gene expression or tell us much about cell types as these characters are not preserved.

We believe the correct scientific approach to this is through falsification of a hypothesis of causation, and that depth of insight available from our analyses into gene regulatory signatures of cell types can help us resolve this. Specifically, if WGD postdates the evolution of novel cell types, we disprove a mechanistic/causative role. We propose two evolutionary models here to help distinguish the alternatives. The figure below is reproduced from Figure 3a of our revised submission. We start by imagining an ancient cell type with a CoRC (the suite of regulatory genes that defines its identity). Both models must explain the cell identities seen on the right of the figure, which are the observed data in living taxa.

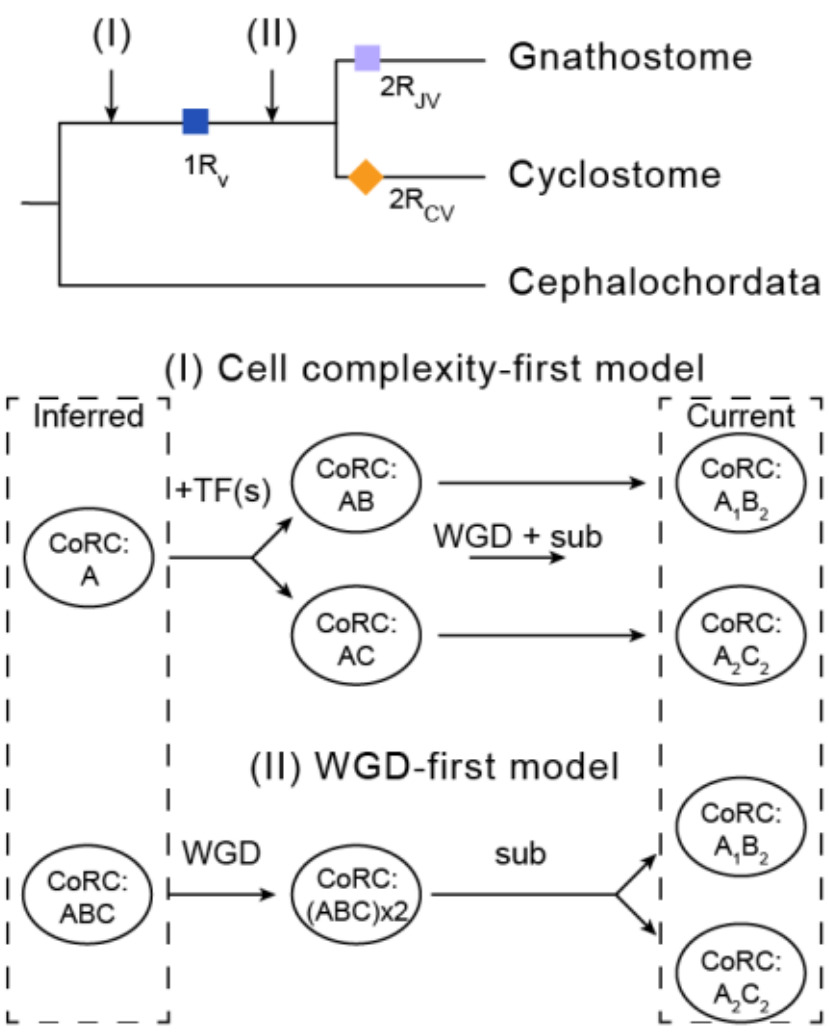

- In Model 1 (cell-complexity-first), the CoRC diversifies into two sister cell types by one or both of them acquiring *new* regulators (neofunctionalization) alongside the ancestral CoRC, causing their individuation. WGD then happens, generating ohnologues of the CoRC members and allowing them to then be differentially deployed in the cell types via passive processes unrelated to the origin of novel cell types.
- In Model 2 (WGD-first), the ancient CoRC *already* possessed the conserved TFs used in individuation of different sister cell types. Following WGD, dosage selection and

subfunctionalization separate CoRC ohnologues into different cell types, driving their individuation (as they now possess different CoRCs).

Both models explain the distribution of ohnologues as markers that we see in our analysis of single cell data. It is important to note that they are not mutually exclusive: given the complexity of brain cell-type evolution, it is possible that both have occurred. The question is which was dominant. As Referee #2 noted, gain-of-function (neofunctionalization) is much harder than loss-of-co-expression (subfunctionalization), which would tip the balance towards Model 2. This, though, is not proof of what *did* happen. However, we have addressed this more definitively in two ways, both of which are made feasible by our new amphioxus adult data.

One way is by identifying concrete examples where we can test the mechanism through analysis and lab work. We chose macroglia evolution as tractable for this. Astrocytes and oligodendrocytes are evolutionarily and developmentally related sister cell types (new Fig. 3b) in the central nervous system. We identify several conserved ohnologue TF gene families having the potential to separate astrocytes and oligodendrocytes. Several key TFs stand out, including Sox8/9/10, Nfia/b/x, and Olig1/2, etc. Many have been experimentally validated as key terminal selectors. Sox9 activates Nfia, and they bind together to drive astrocyte fate[11,12]; Nfia and Sox10 are reciprocal antagonists, through binding to each other[13]; Sox8/10 and Olig1/2 work together to drive oligodendrocyte fate[13]. Using our new amphioxus data, we find amphioxus already possesses radial glia, co-expressing *SoxE*, *Olig*, and *Nfia/b/x* family genes[14]. If model 1 is the case, these TFs would not be predicted to be co-expressed in amphioxus glia, but to be gained in the vertebrate ancestor prior to 1R.

However this alone is still correlation and not proof of mechanism. To do this we need to understand function, which can be done experimentally. We addressed this by generating an amphioxus *SoxE* mutant line (see Fig. 3g), in which glial cells display reduced expression of key TFs as well as the glial marker *Eaat2* (new Fig. 3h). These findings indicate that *SoxE* is important for glial differentiation.

Second, we also undertook a systematic analysis of the new amphioxus brain data as the models make different predictions. Model 1 predicts a more narrow and exclusive expression of vertebrate conserved TFs in amphioxus. Model 2 predicts vertebrate conserved TFs will be used more broadly and less specifically. Our analysis of the amphioxus brain data supports Model 2, and we show this in Fig. 3c, Extended Data Fig. 3, and Extended Data Fig. 6b.

In summary, while we acknowledge the Referees' initial concern regarding the lack of causal evidence, our new data and analysis mean we can infer ancestral states, and we have then been able to follow up on this with analysis and with experiment on macroglia to tie ohnologues to cell-type innovations. We have rewritten the manuscript in several places to accommodate this new work, including in the results (centred on the new Figure 3) and the Discussion.

6. In particular, the subfunctionalization analysis relies on inferred ancestral expression states that are easier to reconstruct when expression is lost rather than gained. This introduces a bias toward detecting subfunctionalization over neofunctionalization. The authors acknowledge this briefly, but the implications are not discussed in depth.

Response 12: We agree with this comment. We acknowledged this more explicitly and make sure it is clearly indicated as a caveat (lines 621-629 and 651 in the Discussion).

7. Similarly, the finding that many paralog families have a dominant member across species is consistent with dosage selection, but it is not clear how such pairs then support distinct cell type identities. These dynamics are central to the argument but are only partially resolved.

Response 13: This question is covered in Response 11 for Referee #2 (new Fig. 3).

8. Species and batch effects may still confound key comparisons

9. The integration across species is handled thoughtfully, using SAMap and other tools, but variance decomposition shows that batch and species effects dominate over cell type signal in the data. It remains unclear whether the observed divergence of paralog expression across species is fully separable from technical and sampling differences. The claim that similar ohnologues are dominant across species is consistent with shared expression bias rather than cell type-specific redeployment. Some key inferences, including those about subfunctionalization, would benefit from additional controls or replication in other datasets.

Response 14: We respond to these two together and are sorry about the misunderstanding, we were not clear enough in detailing exactly how we had dealt with this. As the referee notes, species and batch effects can be significant and this is well documented in previous studies by one of the authors of this manuscript[15]. SAMap finds the closest cell types across species, but this method does not 'correct' expression levels for species and batch effects in a way that would be needed for direct comparison of gene expression levels in the same cell type across species. We therefore avoided this by comparing the expression level of genes only *within* a species. This means species and batch effects are controlled by using data from a single study. The patterns identified by comparison within species were then compared across species (for example in Fig. 2a,b,f and Fig. 3b-g in our original manuscript). We have deleted the variance decomposition analysis on species signals (Fig. 2g in the

original manuscript) to avoid this misunderstanding: this was a hangover from our early exploration of the data, and not relevant to the revised manuscript.

Minor Points

1. The distinction between "association" (a gene being a marker) and "contribution" (each paralogue in a pair marking different cell types) is not always clearly stated. These are conceptually different and should be more clearly separated in the text.

Response 15: We agree we had not been sufficiently precise and specific with these terms and that they should not be considered synonymous. In revising the manuscript, we have necessarily revisited this throughout as we tackle the question of mechanism/causality, and now use much more specific phrasing.

2. The definition of orthogroups and how they relate to ohnologue and SSD families is a central methodological issue. It is not always clear whether overlapping categories are treated separately, nor how double-counting is avoided.

Response 16: Thank you for pointing this out as it is important, and we have reconsidered this to improve our methods. We now separate ohnologue/SSD paralogue orthogroups from ohnologue/SSD paralogue families. We used orthogroups in cross-species analysis and we have made the concept of ohnologue orthogroups or SSD paralogue orthogroups stricter in our revision (lines 373-376). Specifically, at least 3 out of 4 vertebrates having a pair of ohnologues can be defined as ohnologue orthogroups, and similar for SSD paralogue orthogroups. Fewer (339) orthogroups can be defined by both categories compared to those in our original manuscript. However, we obtained similar results as before based on updated orthogroups and reach the same conclusion as previously (new Fig. 4).

3. The role of developmental data is mentioned in several places, but it is not clearly integrated into the argument. Since cell types are defined based on adult and juvenile brains, it is unclear how the developmental origins of cell type families are inferred.

Response 17: As mentioned earlier (please see Response 1 for Referee #1), we now have an amphioxus adult brain dataset for comparison to vertebrates (Fig. 1-3).

Summary

This is a well-executed and technically sophisticated study. It offers an integrated cross-species perspective on the legacy of whole genome duplication in vertebrate neurogenesis and presents important descriptive data. However, the main claims are primarily confirmatory and rely on associations that could reflect general features of gene function rather than WGD-specific

mechanisms. The paper provides a valuable resource and an useful synthesis, but the conceptual advance is modest.

Response 18: Thank you for the positive views on the strengths of our study however, as we mention earlier, we disagree that this is a modest conceptual advance. Our study is the first to show this lack of clear 1-to-1 cell-type homology between amphioxus and vertebrates but to also show that evolutionary relationships are identifiable at the higher hierarchical levels that our evolutionary approach allows. We also show how this fundamental biological unit that underpins the complexity of multicellular organisms, the cell type, has been affected by pivotal genomic events early in vertebrate evolution, and how this has continued to impact vertebrate evolution long after they occurred. While we accept that we had not crystallised the distinction between association and causation in our original manuscript, we believe the additional experiments and analyses we have done address this (new Fig. 3).

Referee #3 (Remarks to the Author):

This study by Zhu et al. investigates the impact of whole-genome duplications (WGD) on the evolution of the vertebrate brain using systematic comparisons of published single-cell transcriptomic data originating from the brains of four vertebrate species (human, mouse, lizard and lamprey) (three amniotes and one cyclostome).

The authors first define cell-type families that share gene expression signatures (transcription factor codes) common to those vertebrates. By comparing gene expression in these cell-type families to data taken from an outgroup in which WGD did not occur, they report an enrichment of genes retained after WGD (“ohnologues”) among the differentially expressed genes (or marker genes), not seen for genes duplicated by other mechanisms (small scale duplications or “SSD paralogues”).

The authors observe a similar pattern with two different sets of data: cerebellar nuclei in amniotes and a subset of the Human Cell Atlas.

Comparisons within the duplicated gene pairs reveal that paralogues most often diverge from their ancestral role (“subfunctionalization”) across cell types and that their expression follows a principle of dosage selection. These observations concerning neuron-type evolution appear to be novel, although similar observations have been made before at tissue scale using bulk RNA-sequencing.

The presented conclusions are original, offer hints about possible mechanisms underlying cell-type evolution and connect them to large-scale genomic changes. However, the link between WGDs and cell-type evolution remains correlative despite the authors claiming that they “contributed to” (lines

479f) cell-type evolution. This latter characterization is probably too strong.

We would welcome clarifications and new analyses to strengthen the paper, as detailed below:

Response 19: Thank you for both the generally positive summary and suggestions here. We agree "contribution" was too strong for our original manuscript as we didn't resolve the "mechanism" in our original manuscript. As discussed above, we have altered the text accordingly and in addition we propose evolutionary models and added new datasets, analyses, and experiments to support the causal roles of WGD to vertebrate cell type evolution (at least for macroglia and cerebellar nuclei evolution, Fig. 3 and Fig. 6 (please also refer to our Response 11 to Referee #2 for detailed explanation).

Major points:

1. Ohnologue classification: because all the findings of this study build upon the classification of duplicated genes as ohnologues or other paralogues, the applied approach should be better justified.

a. Ohnologues are, in the ms, inferred across all vertebrates collectively; yet, the lamprey lineage has undergone a separate WGD and therefore has a more complicated paralogue relationship. Would this affect the analysis and if so, how is it controlled for?

Response 20: We agree that the identification of ohnologues is a key part of the paper, and we updated our ohnologue lists and have provided methods description and comparison based on updated assembly and annotations, and a method comparison in Supplementary Text. With respect to lamprey ohnologues, the best way to detect these would be through using several cyclostome genomes with comparison to outgroups without WGD. This is challenging at present due to the relative lack of high-quality genome and gene set data from cyclostomes. Further, due to extensive loss of cyclostome duplicated genes, it is difficult to separate lamprey ohnologues into 1R and 2R[5] without this. However, we do not think that this has an impact on our analysis. The Ohnologs-v2.0 pipeline compares paralogous blocks within species, across vertebrates, and to outgroup species without WGD. Since the first WGD is shared by jawless and jawed vertebrates and the further WGDs in jawless vertebrates are due to hexaploidization, we think identifying ohnologues collectively does not affect outcomes, as a fair percentage of ohnologues (>= 70%) are identified as shared between our (updated version) and Ohnologs v2. Additionally, we compared our ohnologue detection results with and without lamprey, and the differences are trivial (<0.2% in terms of pooled ohnologue differences, please refer to Supp. Text for details). Further, we used Marlétaz and colleagues' lamprey ohnologues to do the association study, and found the same pattern (please see lamprey results in Fig 2a,b). This also relates to other points below (see Responses 23 and 24 for Referee #3) and, as mentioned below, we have included a section on Ohnologue identification in the Supplementary Text exploring this in more detail.

b. An asymmetric gene loss during R2 of WGD in jawed vertebrates is mentioned, resulting in different gene retention rates for the alpha and beta lineages (with higher retention rates in one of them). The different lineages should probably be analysed separately, given that they might have evolved under different selection constraints.

Response 21: This is an important point and, as discussed above (Response 6 to Referee #1), we have now performed ohnologue association and enrichment analysis based on 'alpha' ohnologues and 'beta' ohnologues. We found additional evidence (stronger association with markers and more important GO enrichment) supporting 'alpha' is more important, alongside having more retained genes (new Fig. 2b, Extended Data Fig. 4b, Extended Data Fig. 5b, Supp. Fig. 6,7).

c. Gene age can make establishing the true historical relationship between paralogues difficult; for example, old SSDs may be detected as WGDs (see https://academic.oup.com/gbe/article/15/10/evad174/7287110). Was this examined and controlled for in the paralogue classification? If so, how? If no, it probably should.

Response 22: we agree with both these points, and we have carried out additional work accordingly. These are dealt with in full in Response 6 to Referee #1 and Response 10 to Referee #2, but to briefly recapitulate here we found our conclusions hold after considering both the different retention rates at 'alpha' and 'beta', and when considering SSD duplicate gene age.

d. The authors state: "To assess the robustness of our ohnologue predictions, we compared our results to the Ohnologs v2 database (http://ohnologs.curie.fr), finding that 80% of human and 66% of mouse ohnologues in our dataset were also present in the database." (lines 595f)

Yet, the proportion of the ohnologues listed in the Ohnologs v2 database that were identified in the authors' dataset is not stated. These numbers should be given.

Response 23: We agree with this. We have updated the ohnologue sets to higher quality based on more vertebrates and have done the reverse comparison (please refer to "Ohnologs-v2.0 and comparison between our updates and v2.0" in Supp. Text lines 113-142). More than 75% of human ohnologues identified in our dataset were also present in Ohnologs-v2.0, and vice versa. Similarly, over 70% of mouse ohnologues overlapped between the two datasets. These suggest both versions are largely consistent sets of ohnologues.

e. The authors could consider other methods to classify gene duplications and compare them to those presented in the ms (e.g. https://github.com/qiao-xin/DupGen_finder, https://genomebiology.biomedcentral.com/articles/10.1186/s13059-019-1650-2)?

Response 24: We also thank the Referee for pointing us to these other methods of duplicate identification. We have tested them for detecting ohnologues and SSD paralogues; however, none consider 'across vertebrate robustness' and 'outgroup' without WGD and therefore they result in greatly varied numbers of identified ohnologues. We have provided a full comparison and method description in the supplementary text to show how methods compare and explain why we adopted our chosen method.

2. Paralogue contribution to cell type evolution:

f. The authors write that "a single-cell dataset from amphioxus at late neurula stage, filtered to retain only CNS cells, was used as a 'control' for analysing relationships between extensive SSD paralogues and DEGs" (lines 171f). Could the fact that the control dataset originates from an early developmental stage (unlike the other datasets) affect this analysis? It is conceivable, if we assume that retained paralogues are more likely to be TFs or regulators, and therefore that they are more highly expressed in developmental datasets, that these genes among the markers will be over-represented? If so, this bias should be corrected.

Response 25: This point has been considered above (Response 1 to Referee #1) and as detailed there, we now have an amphioxus adult brain dataset, allowing comparison of adult cell atlas data throughout.

g. We did not see mention or discussion of the fact that, in the paralogue-pair analysis, the patterns differ between lamprey and jawed vertebrates at both cell-type and -family levels (Fig. 2f and Extended Data Fig. 3i). Could this be a result of the fact that the inference about ohnology is not appropriate for lamprey (see above)?

Response 26: We have performed analysis on another version of ohnologues identified based on orthology assignments from Marlétaz and colleague's results[5]. We found our results and conclusions are consistent. The outcomes are in the updated Fig. 2b.

3. Inference of sub- vs. neofunctionalization:

h. Inferences about sub- vs. neo-functionalization after WGD seem to be very sensitive to the details of the data analysis and on outgroup selection (discussed in https://www.nature.com/articles/s41588-018-0162-4 and https://www.nature.com/articles/s41588-018-0163-3). In this study, no outgroup was used to infer ancestral expression states. Instead, the expression of genes was compared between species, and the ancestral state inferred if a majority of species (e.g. 3 out of 4 vertebrates) showed expression (line 744f). Could this choice affect the observed result of subfunctionalization, if so, in what ways? The missing outgroup issue should be remedied, if possible.

Response 27:

Previous work on inferring ancestral states using an outgroup has used bulk transcriptome expression from major tissues for such comparison. We do agree that this was justified at the time, as while bulk RNAseq data mask cell type complexity in a tissue, it is unequivocal that major tissues such as 'brain' and 'gut' can be homologised between these distant lineages at this level. However, when we analyse at the cell-type level using single cell data, our results show the differences are too profound for this to be reliable: specifically, our new analysis of amphioxus adult brain shows there are no vertebrate-style cell-types in the adult amphioxus, except at such high hierarchical levels that they lose useful resolution. We hence think inferring ancestral states based on comparison of several vertebrates is more appropriate than using amphioxus.

i. High-copy number SSDs were excluded without explanation (lines 728f & 757f). This decision should be explained and justified, and a more detailed definition of SSD should be provided.

Response 28: We should have been clearer on this. Every genome has a small number of gene families which exist in high copy number. We excluded these for two reasons:

1. Many duplications are lineage specific, or of uncertain orthology between species if shared, due to high gene turnover.
2. Keeping these genes in risks skewing the SSD pair comparisons towards these high copy number families.

We have updated the text to more explicitly explain this (lines 1067-1070 & 1097-1099).

Minor points:

Response 29: We really appreciate Referee #3 for their detailed look at the code and suggestions and other comments here. We essentially agree with their comments/suggestions and have addressed them in the revision. We also made several changes to figures and legends to make them 'smarter'.

1. Mismatch between parameters stated in methods and code:

There are several mismatches between the parameters found in the code provided by the authors and the methods section of the paper.

2. SAMap

Methods (line 605) state: "We then performed cross-species mapping using the SAMap run function with 5 iterations, edge weight calculated and updated by Pearson correlation (hom_edge_mode = "pearson"), and 20 cross-species edges per cell (crossK = 20). Mutual nearest neighbourhoods were independently calculated between each pair of species (pairwise=True)."

However, the corresponding code (https://github.com/DiracZhu1998/WGD2celltype_evolution/blob/main/0.atlas/6.SAMap/SAMap_run_vertebrate_neurons.py) suggests that the authors used "crossK = 30".

Response 30: We used “crossK = 30” and we have corrected it accordingly (Methods, line 810).

3. Seurat marker calculation

Methods (lines 644-646) state the following parameters for Seurat’s FindAllMarkers “min.pct = 0.01, logfc.threshold = 0.58, test.use = "wilcox", only.pos = TRUE”, however the code (https://github.com/DiracZhu1998/WGD2celltype_evolution/blob/main/3.markers/DEGs_Seurat/FindMarkers.R) only seems to use “test.use = "wilcox", only.pos = T”.

The code also shows that the data were subsampled to 3,000 cells per category prior to the marker calculation. This is currently not described in the methods.

```
sampled_df <- sampled_df %>% group_by(args$label) %>% slice_sample(n = 3000) %>% ungroup()
obj <- subset(obj, cells = as.character(sampled_df$cellname)))
```

Response 31: Thank you for pointing this out. We subsampled to 3,000 cells to minimize potential bias caused by imbalanced cell-type numbers during marker detection, and we have added corresponding descriptions to clarify this (Methods, lines 917-919).

4. Analysis questions:

a. Why was SAM used for preprocessing when the counts were normalized again using the scrattch pipeline?

Response 32: Sorry for the confusion (script: Run.iterative_clustering.R, lines 21-23), we did not normalise twice. Instead, we performed scrattch pipeline directly to the count matrix (UMI), not to the SAM normalised matrix. This follows the scrattch pipeline (https://taxonomy.shinyapps.io/scrattch_tutorial/#section-data-selection), where the counts are first converted to counts per million reads (CPM) then log2-transformd. We have clarified this in the corresponding Methods of the revision (lines 713-715).

b. The SAM code is missing from the GitHub repository.

Response 33: The SAM code is not included because it is a built-in component of the SAMap package. We ran SAM by invoking the SAMAP function, which internally performs the SAM preprocessing, so no separate code was required. We have clarified this in the Methods (line 711-713).

c. Iterative clustering: Why were different values chosen for split.size between species? The data were downsampled to roughly the same numbers of cells; hence the reason why they were treated differently at this step is not clear.

Response 34: We agree explanation was needed. Our aim was not to define cell types at very high resolution; approximately 200 clusters per species provided sufficient granularity to capture major

subtypes. Because clustering can be affected by factors such as cell number, data quality, species, sampling composition, single-cell/single-nucleus, and cell complexity, we adjusted split.size to achieve cluster numbers at similar level across vertebrates. We have explained it in corresponding part of the manuscript (Methods, lines 791-793).

d. Trinarization score: The Trinarization score was calculated differently between analyses (min. % of cells was 10% for whole brain data, 20% for cerebellar nuclei data), detailed in lines 741f. & 794f. Why were different parameters chosen for the different datasets? Please harmonize or justify.

Response 35: Thank you for pointing this out and prompting us reconsider this analysis. We chose 20% because we observed that many key TFs (experimentally validated) were differentially expressed across CN excitatory neuronal subtypes, with expression detected in >20% of less-expressing cell types. This likely reflects the serial homologous excitatory neurons among different CNs[16], where key TFs may not strictly distinguish them in a binary form (Methods, lines 1152-1156). In the revised version, we focused only on TFs (Fig. 6d) and adopted an alternative approach (lines 562-564) to investigate conserved TFs potentially separating CNs.

5. Visualization and labeling issues:

a. Figure/Legend label mismatch

The cyclostome-specific hexaploidization is referred to as “2Rcy” in the figure legend (Figure 1) but shown as “2Rcv” in the figures (Figs. 1a, 3a).

Response 36: We have corrected to “2Rcv” in the Figure 1a legend.

b. Figure legend description

EDFig. 10 f,g: The legend describes a dot plot whereas the figure shows a violin plot.

Response 37: We have corrected in the revision (new EDFig.10 g,h).

c. Color bar for multi-species dot plots

Multi-species dot plots lack a color bar (Figs. 1c, 4g, EDFigs. 1a-d, 2d, 5e, 7e) that encodes strength of expression for the different species or cell type families.

Response 38: We have added colour bars to Figure 1c, EDFigs. 7e, 8e in the revision. For others, we converted them into single-colour dot plot (Extended Data Fig. 1d and Supplementary Fig. 2).

d. Extended Data Fig. 3a-f: it is hard to distinguish the datasets or gene sets shown in the panels. Horizontal bars labeling a-b as human eye+lung data, c-d as TF, e-f as TF targets might improve readability.

Response 39: Thank you for the suggestions. We have amended with labelling in the revision to improve readability (Extended Data Fig. 4d,e, h-k).

e. Fig. 3a is not easy to understand, e.g. the grey and white boxes are not explained in the legend.

Response 40: We agree and we have added the grey and white boxes meanings and provided more detailed description of the model in the revision (Fig. 4a) to improve readability.

Referee #3 (Remarks on code availability):
The code and data provided are a usable resource for the community. We have not installed and run the code, but the repository includes configuration files for conda environments and the README files for most of the analyses described in the paper.
As mentioned above some parameters described in the methods do not match the parameters found in the code. The authors should therefore check the consistency of the methods and code.

Referee #4 (Remarks to the Author):
I co-reviewed this manuscript with one of the Referees who provided the listed reports.
Referee #5 (Remarks to the Author):
I co-reviewed this manuscript with one of the Referees who provided the listed reports.

References:

1. Dai, Y. *et al.* Evolutionary origin of the chordate nervous system revealed by amphioxus developmental trajectories. *Nat Ecol Evol* https://doi.org/10.1038/s41559-024-02469-7 (2024) doi:10.1038/s41559-024-02469-7.
2. Wagner, G. P. *Homology, Genes, and Evolutionary Innovation*. (Princeton University Press, Princeton ; Oxford, 2014).
3. Muller, G. B. & Wagner, G. P. Novelty in Evolution: Restructuring the Concept. *Annu. Rev. Ecol. Syst.* **22**, 229–256 (1991).
4. Corrigan, E. K. *et al.* Conservation and alteration of mammalian striatal interneurons. *Nature* **647**, 187–193 (2025).
5. Marlétaz, F. *et al.* The hagfish genome and the evolution of vertebrates. *Nature* **627**, 811–820 (2024).
6. Arendt, D. *et al.* The origin and evolution of cell types. *Nat Rev Genet* **17**, 744–757 (2016).
7. Simakov, O. *et al.* Deeply conserved synteny resolves early events in vertebrate evolution. *Nat Ecol Evol* **4**, 820–830 (2020).
8. Stolt, C. C., Lommes, P., Friedrich, R. P. & Wegner, M. Transcription factors Sox8 and Sox10 perform non-equivalent roles during oligodendrocyte development despite functional redundancy. *Development* **131**, 2349–2358 (2004).

9. Liu, Z. *et al.* Induction of oligodendrocyte differentiation by Olig2 and Sox10: Evidence for reciprocal interactions and dosage-dependent mechanisms. *Developmental Biology* **302**, 683–693 (2007).

10. Donoghue, P. & Purnell, M. Genome duplication, extinction and vertebrate evolution. *Trends in Ecology & Evolution* **20**, 312–319 (2005).

11. Rowitch, D. H. & Kriegstein, A. R. Developmental genetics of vertebrate glial–cell specification. *Nature* **468**, 214–222 (2010).

12. Kang, P. *et al.* Sox9 and NFIA Coordinate a Transcriptional Regulatory Cascade during the Initiation of Gliogenesis. *Neuron* **74**, 79–94 (2012).

13. Glasgow, S. M. *et al.* Mutual antagonism between Sox10 and NFIA regulates diversification of glial lineages and glioma subtypes. *Nat Neurosci* **17**, 1322–1329 (2014).

14. Hines, J. H. Evolutionary Origins of the Oligodendrocyte Cell Type and Adaptive Myelination. *Front. Neurosci.* **15**, 757360 (2021).

15. Musser, J. M. & Wagner, G. P. Character trees from transcriptome data: Origin and individuation of morphological characters and the so-called "species signal". *J Exp Zool Pt B* **324**, 588–604 (2015).

16. Kebschull, J. M. *et al.* Cerebellar nuclei evolved by repeatedly duplicating a conserved cell-type set. *Science* **370**, eabd5059 (2020).

Response to Referees

Zhu et al

Referees' original comments are reproduced below, with our response in blue text.

Point-by-point response:

Referee #1 (Remarks to the Author):

The revised manuscript includes expanded analyses and clarifications, and some of the claims have been moderated, as reflected in the revised title.

The authors refine their definitions of ohnologue and SSD paralogue orthogroups, separate alpha and beta ohnologues, benchmark their gene sets against external databases, and perform additional robustness checks, including analyses with and without lamprey. These additions improve the technical rigor of the study.

Upon revisiting the manuscript, I find that most of the principal observations extend well-established principles of gene duplication into a single cell comparative framework rather than introducing new conceptual advances.

The enrichment of ohnologues among vertebrate brain cell type markers, the widespread asymmetric expression of paralogues, and the prevalence of subfunctionalization across cell types are broadly consistent with established principles for WGDs. What is new here is the resolution and comparative scope, with these patterns being shown across multiple vertebrate species at single cell resolution.

The enrichment of WGD derived genes in conserved vertebrate cell type families relative to amphioxus represents the strongest advance. Overall, the novelty of the study lies more in its technical integration and comparative breadth than new ideas on how new cell types evolve.

The authors now incorporate an adult amphioxus brain dataset as the outgroup against which vertebrate patterns are interpreted. This is an important addition. However, it requires quality control comparisons (e.g., sequencing depth, gene detection rates, etc) demonstrating that the amphioxus dataset is technically comparable to the vertebrate datasets.

This is particularly important because amphioxus effectively serves as the reference baseline for the enrichment analyses. If differences in marker detection or cell type resolution reflect dataset sensitivity rather than biology, the apparent excess of vertebrate ohnologues could be influenced by technical artifacts.

Response 1: This is an important point, and we should have provided and compared more technical

statistics. The amphioxus datasets encompass a broad gene repertoire and a substantial number of transcripts with relatively low mitochondrial expression ratio (Supplementary Fig. 1A,B). Most known major cell types of amphioxus brain were recognised within it, so we are confident as to its quality (Supplementary Fig. 1C and Supp. Table 2). We now also provide more detailed statistics on the preprocessing of the amphioxus brain datasets in the supplementary text (see Supp. Text: Sufficient cell-type sampling and high-quality of amphioxus brain datasets).
With respect to the cell-type resolution question, this aligns with a question raised by Referee# 3, and we answer these together in Response 10 for Referee #3.

A limitation of the manuscript is that it does not firmly link specific gene duplication events to specific cell type divergences. The authors show that ohnologues are enriched among marker genes and frequently diverge in expression, and they provide illustrative case studies (e.g., macroglial programs) suggesting how paralogue partitioning may have accompanied cell type diversification. However, they do not demonstrate that particular duplication events preceded and enabled defined neural cell type splits in a temporally resolved manner.
In this sense, the study supports the view that WGDs shaped the gene landscape of vertebrate brains, but it does not provide the strongest possible evidence that they were involved in the generation of new cell types. The use of "contributed" in the title feels conceptually ambiguous.
Response 2: We take the point raised here by the Referee, and as described elsewhere we have toned down the title and other parts of the manuscript on these issues.

Two concerns from my initial assessment remain. First, the framing of the analysis as a comparison between WGD and SSD remains narrow. By focusing on the duplication mechanism, the manuscript does not attempt to evaluate alternative sources of increased regulatory complexity, such as expansion of cis-regulatory elements or rewiring of GRNs. Second, if WGDs were sufficient to drive the emergence of new cell type families, one might expect lineage specific WGDs to have produced clear examples of novel, conserved cell type repertoires.
Response 3: In the absence of comprehensive single-cell brain ATAC-seq datasets (some bulk datasets exist, e.g.[5], but are not good enough for this sort of analysis) across a broad range of vertebrate species, it is not possible to disentangle additional layers of regulatory evolution, particularly those involving cis-regulatory elements and regulatory network rewiring. Resolving this would require substantial work, minimally single-cell ATAC-seq profiling of brain tissue from key phylogenetic positions including amphioxus, lamprey, and lizard, and would require several years to complete. As we pointed out previously, we think this is at the level of a separate study. However we did perform some regulation analyses where we felt this was appropriate (i.e. related to gene duplication). This was reported in the previous revision, specifically looking at 'regulation motifs'

contributed by WGD and SSD in human and mouse, the full data are in Supp. Fig. 20 and it is mentioned in the results.

The manuscript provides a well-executed comparative analysis, and it will be of interest to the field. Yet, I found the framing of causality throughout the manuscript to be ambiguous. Causality is continuously implied and then walked back, resulting in a lack of clarity regarding the strength of the conclusions.

Response 4: As mentioned elsewhere all three Referees suggested we soften our tone on this. We agree and we have done this throughout this version of the manuscript.

Referee #2 (Remarks to the Author):

I thank the authors for their thoughtful responses to my remarks. While I am a little ambivalent, I think they have got about as far as one can in what is fundamentally a very hard model to assess (temporal confounding, not open to causal experiments, etc). I'm hard pressed to think of real angles unaddressed that would not require dramatically more data. That data may come with time but in the meantime, this is a compelling presentation of a model that one can accept as standard.

That said, while the fundamental claims within the paper are compelling, they really don't support the modest overstatement inherent in the title.

Response 5: We thank Referee #2 for their positive comments on our revision. As mentioned above we agree that the title and several statements were still a bit strong and have therefore revised the title and elsewhere to adopt a more cautious and softened tone throughout.

Referee #3 (Remarks to the Author):

We thank the authors for their revisions.

Strengths:

the new data collected from adult amphioxus and additional analyses (alpha/beta WGD lineages, SSD gene age) strengthen the authors' finding that ohnologs, especially from first WGD, are associated with increased cell type complexity and evolution in vertebrates

the sc/snRNAseq data from adult amphioxus seem to be of high quality and will likely be useful to others interested in cell-type evolution. The authors do not comment on the number of sequenced cells in comparison to total cell numbers present in amphioxus, but the 5568 sequenced cells (3217 neurons + 2351 non-neurons) compared to a total of ca. 20,000 neurons [PMID: 22676056] seem to be a sufficiently high fraction (~16%). It remains not totally clear to us, however, whether the different clustering procedure might affect the conclusions drawn about cell type relationships (see below).

Response 6: We thank Referee #3 for this point. As noted, our brain dataset includes 3,217 neurons.

The figure of approximately 20,000 neurons from PMID: 22676056 is the figure for the whole CNS, so the brain only will have substantially fewer than this. With the vast size of vertebrate brains, especially human, single cell datasets generally capture far less of a percentage of vertebrate brain cells than our amphioxus dataset. The important point, however, is whether we are capturing rare cell types. Amphioxus brains contain some well-described rare cell types, like dopaminergic neurons and infundibular cells, and we clearly identify these. Therefore, we think our sampling is adequate to identify most cell populations in the adult amphioxus brain. But we agree that this should be acknowledged (now in the Supplementary Text).

Causal experiments are admittedly hard. The authors focus on the macroglia/astrocyte-oligodendrocyte cell type relationship and investigate a SoxE knockout in Amphioxus, making a reasonably convincing case for a broader and less essential expression of SoxE in Amphioxus glia compared to knockouts of Sox8/9/10 in vertebrates. This supports their hypothesis of a subsequent individualisation of sister cell types (astrocytes and oligodendrocytes) in vertebrates.

We appreciate the detailed comparison of ohnologue detection methods (Doubletrouble, DupGen_finder, and Ohnologs-v2.0) added by the authors.
Response 7: We thank Referee #3 for their good summary and positive comments here. We essentially appreciate and agree with their suggestions on the details of our figures and legends.

Remaining questions:
The manuscript contains duplicated figures with different data for which the discrepancy is not adequately explained:
What is the difference between SFig. 6B and EDFig. 4C;
bw EDFig 4B and SFig. 6A;
bw Fig 2B and SFig 6C?
Response 8: These analyses are fundamentally the same, but they are conducted at different cell-type resolutions/levels. We now add additional information (family vs. cluster level listed in Supp. Table 2) to clarify the difference between these figures in the corresponding legends.

The OligB expression is barely visible in the transverse section of Amphioxus T1 (Fig 3d and EDFig. 6f)? The authors should consider changing the colors (and to be colorblind-friendly) and/or image intensity.
Response 9: Thank you for the suggestion and we have changed accordingly to colourblind-friendly colours and believe they are easier to see now. In addition, we have added a magnified view of the neural tube to facilitate interpretation in Fig. 3d and EDFig. 6f.

Methods question: clustering analysis for Amphioxus data differs from vertebrate datasets, resulting in a much lower number of clusters for Amphioxus (23 vs. 141-241 clusters). The marker genes are then compared, showing broader expression in Amphioxus leading to the conclusion that it represents a lack of sister cell type diversification pre WGD. Is this conclusion affected by (a result of) the clustering procedure?

Response 10: Given the cell number in amphioxus brain single-cell datasets (~5,600 cells), increasing the clustering resolution to generate cluster numbers to a similar level of vertebrate datasets will likely result in over-partitioning and unstable clusters. For the biological question we aim to address—whether vertebrate-specific novel cell-type families are present in amphioxus—we believe the resolution is sufficient and that increasing it would not affect the outcomes. Our conclusions do not depend on fine-grained subdivision of amphioxus cell populations but rather on broader transcriptional co-expression patterns. Even if we were to partition the amphioxus brain into a much larger number of smaller clusters, the key TFs remain broadly co-expressed. As shown in the UMAP below (shown for vertebrate macroglia key TFs as an example) , these TFs are relatively pan-expressed rather than confined to spatially restricted subregions within clusters. Therefore, increasing clustering resolution or changing the clustering method would not alter any our biological interpretations.

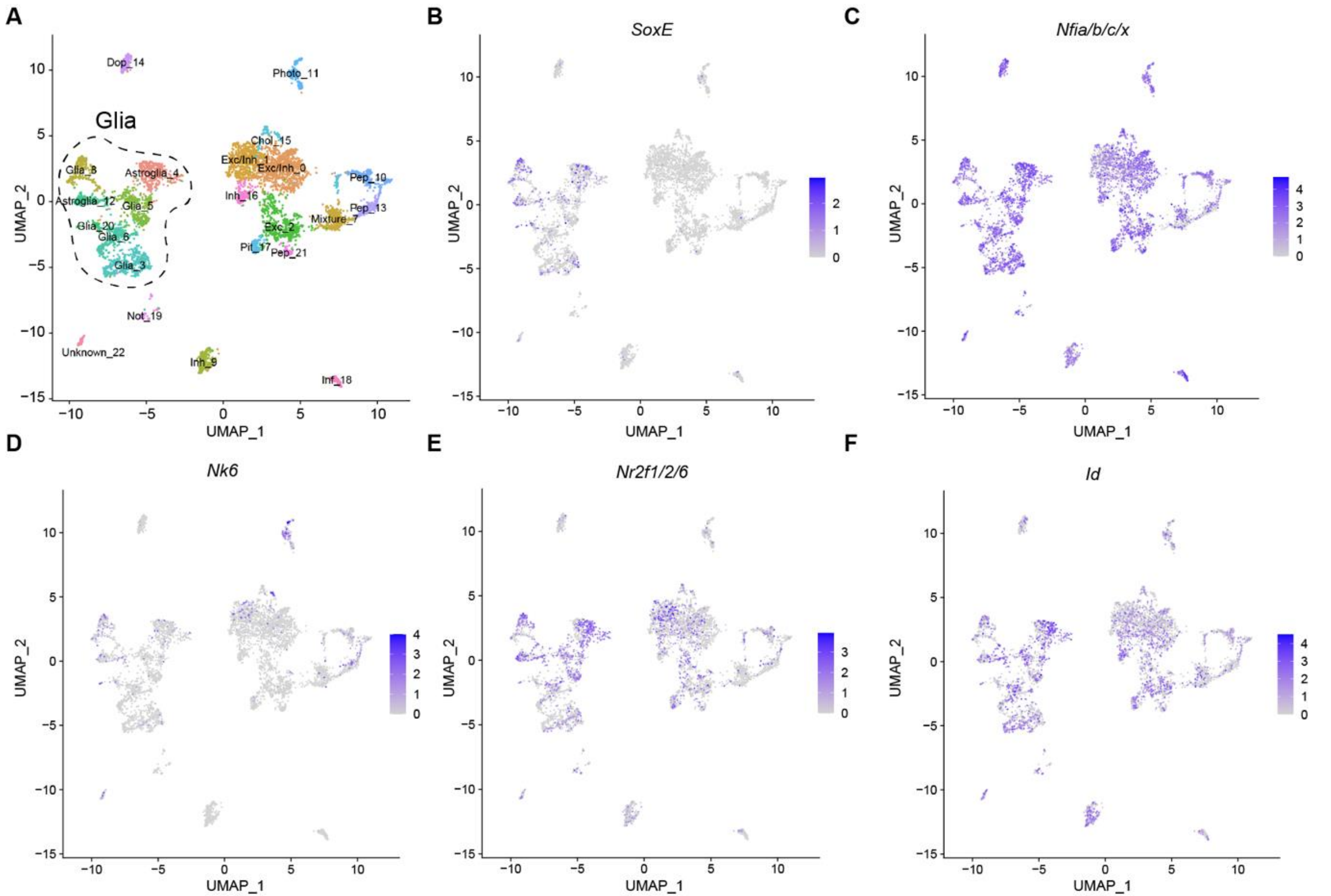

(**A**) UMAP visualization of identified adult amphioxus brain atlas with cell-type annotation. (**B-F**) Key conserved TFs (except Olig genes which have limited expression in adult amphioxus brain) separating astrocytes and oligodendrocytes. Colour of dots represent normalised expression level. These UMAP plots show the board expression pattern of these key TFs in glial population.

Minor comments:
Is there a typo in the EDFig 6h figure legend? Should it say “N4 stage” instead of “T1 stage”?
Gene names for (Tbr1, Tbx21 and Eomes) in lamprey Fig. 4d. Why do lamprey gene IDs have a “MSTRG” or “PMZ” prefix?

Response 11: Thank you for spotting the typo, this has been updated. For the lamprey genes shown in Fig. 4d, we used the gene annotation file from Lamanna and colleagues[9]. The prefixes “MSTRG” and “PMZ” are the prefix of gene IDs in the lamprey annotation. We retained these to ensure traceability for readers who may wish to cross-reference the annotation file. Looking back on this we realised we had also missed the bootstraps off the trees in figures ED7g and 8d, and have now included these.

The authors should consider softening the claim “Fig. 4 | Divergence of paralogues drive cell-type evolution.” (line 450) perhaps to “... are associated with cell-type evolution”?

Response 12: We agree the previous title should be softened as suggested by all three Referees and we have changed it accordingly.

Fig. 4g has species annotations that differ from the others, e.g. “Hsap” vs “H. sap.” elsewhere.

Response 13: Thank you for spotting this, we have revised the species label in Fig. 4 to “*H. sap*.” to ensure consistency.

Referee #4 (Remarks to the Author):
I co-reviewed this manuscript with one of the reviewers who provided the listed reports.

Referee #5 (Remarks to the Author):
I co-reviewed this manuscript with one of the reviewers who provided the listed reports.

References:

1. Horie, R. *et al.* Shared evolutionary origin of vertebrate neural crest and cranial placodes. *Nature* **560**, 228–232 (2018).
2. Abitua, P. B., Wagner, E., Navarrete, I. A. & Levine, M. Identification of a rudimentary neural crest in a non-vertebrate chordate. *Nature* **492**, 104–107 (2012).

3. Markos, A. *et al.* Cell type and regulatory analysis in amphioxus illuminates evolutionary origin of the vertebrate head. *Nat Commun* **15**, 8859 (2024).
4. Muller, G. B. & Wagner, G. P. Novelty in Evolution: Restructuring the Concept. *Annu. Rev. Ecol. Syst.* **22**, 229–256 (1991).
5. Marlétaz, F. *et al.* Amphioxus functional genomics and the origins of vertebrate gene regulation. *Nature* **564**, 64–70 (2018).
6. Candiani, S., Moronti, L., Ramoino, P., Schubert, M. & Pestarino, M. A neurochemical map of the developing amphioxus nervous system. *BMC Neurosci* **13**, 59 (2012).
7. Goriely, A. Eighty-six billion and counting: do we know the number of neurons in the human brain? *Brain* **148**, 689–691 (2025).
8. Williams, R. W. Mapping Genes that Modulate Mouse Brain Development: A Quantitative Genetic Approach. in *Mouse Brain Development* (eds Goffinet, A. M. & Rakic, P.) vol. 30 21–49 (Springer Berlin Heidelberg, Berlin, Heidelberg, 2000).
9. Lamanna, F. *et al.* A lamprey neural cell type atlas illuminates the origins of the vertebrate brain. *Nat Ecol Evol* https://doi.org/10.1038/s41559-023-02170-1 (2023) doi:10.1038/s41559-023-02170-1.
