# Supplementary material for: Whole-genome duplication shaped cell-type evolution in the vertebrate brain
Source: Nature. 2026 Jun 10;656(8128):659–69. doi: 10.1038/s41586-026-10629-x (PMC13489949; doi:10.1038/s41586-026-10629-x)
Supplement: Supplementary file 2 [file 41586_2026_10629_MOESM1_ESM.pdf]

nature portfolio

https://doi.org/10.1038/s41586-026-10629-x

Supplementary information

Whole-genome duplication shaped cell-type evolution in the vertebrate brain

In the format provided by the authors and unedited

Supplementary Information for

Whole genome duplication shaped cell type evolution in the vertebrate brain

Yuanzhen Zhu[1,*], Shuai Zhang[2], Jiankai Wei[1,3,4], Diego Dolgetta-Garcia[1], Katia Jindrich[1], Huimin Liu[2], Chenggang Shi[2,5,6], Rongrong Pan[2,7], Yuwei Chen[2], Yan Xu[2], Qiye Li[8,9], Günter P. Wagner[10,11], Peter W.H. Holland[1], Guang Li[2,*], Sebastian M. Shimeld[1,*]

Affiliation:

[1]Department of Biology, University of Oxford, Oxford, OX1 3SZ, UK. [2]State Key Laboratory of Cellular Stress Biology, School of Life Sciences, Xiamen University, Xiamen, China. [3]Fang Zongxi Center for Marine EvoDevo, MoE Key Laboratory of Marine Genetics and Breeding, College of Marine Life Sciences, Ocean University of China, Qingdao 266003, China. [4]MoE Key Laboratory of Evolution and Marine Biodiversity, Institute of Evolution and Marine Biodiversity, Ocean University of China, Qingdao 266003, China. [5]Mountain Ecological Restoration and Biodiversity Conservation Key Laboratory of Sichuan Province, Chengdu Institute of Biology, Chinese Academy of Sciences, Chengdu 610213, China. [6]China-Croatia Belt and Road Joint Laboratory on Biodiversity and Ecosystem Services, Chengdu Institute of Biology, Chinese Academy of Sciences, Chengdu 610213, China. [7]Department of Basic Medical Sciences, Qiannan Medical College for Nationalities, Duyun, Guizhou 558000, China. [8]State Key Laboratory of Genome and Multi-Omics Technologies & Shenzhen Key Laboratory of Forensics, BGI Research, Shenzhen 518083, China. [9]BGI Research, Wuhan 430074, China. [10]Department of Ecology and Evolutionary Biology, Yale University, USA. [11]Department of Evolutionary Biology University of Vienna, Vienna, Austria.

* Corresponding authors:

yuanzhen.zhu@biology.ox.ac.uk (Y.Z.), guangli@xmu.edu.cn (G.L.), and sebastian.shimeld@biology.ox.ac.uk (S.M.S.)

Table of contents

Supplementary Table Description Page 3

Supplementary Text Pages 4-11

Sufficient cell-type sampling and high-quality of amphioxus brain single-cell atlas

Identifying homologous cell types between amphioxus and vertebrates in the hypothalamus

Comparison of ohnologue detection methods

WGD vs SSD at additional levels

Comparative analysis between vertebrate and amphioxus regarding regional identity programmes

Supplementary Figures Pages 12-34

SI References Pages 34-35

Supplementary Tables (available as separate files)

Supplementary table 1: Marker genes and relevant citations for vertebrates and amphioxus

Supplementary table 2: Refined cluster annotation in all 5 species

Supplementary table 3: Conserved transcription factor genes, orthogroups, and relevant citations

Supplementary table 4: Key conserved transcription factors genes and orthogroups between macroglia types

Supplementary table 5: Macroglia markers

Supplementary table 6: Genes contributing to regional and cell-type identity

Supplementary table 7: Cerebellar nuclei markers and ohnologues

Supplementary table 8: Key conserved transcription factors for cerebellar nuclei excitatory neurons

Supplementary table 9: Datasets used for gene tree construction and ohnologue identification

Supplementary table 10: In situ hybridisation probe details

Supplementary table 11: Amphioxus bulk RNA-seq data details

Supplementary Text:

Additional analyses and validation supporting the main results.

Supplementary Figures (S1-S22):

Additional figures supporting the main results.

Supplementary Text

Sufficient cell-type sampling and high-quality of amphioxus brain single-cell atlas

Our amphioxus adult brain datasets contain 3217 neurons (and 2,351 non-neuronal cells), out of approximately 20,000 neurons in the whole amphioxus CNS[1] (~16%). The brain have substantially fewer cells than this. Human and mouse single-cell brain datasets had 100,000 neurons out of 61–99 billion in human brains[2] (~1.6×10^{-4} %) and ~70,000 neurons out of ~75 million in mouse brains[3] (~0.09%). Despite this sampling, major cell groups and cell types were reliably identified for both amphioxus and vertebrates (see Supp. Table 2). Our additional analysis on the hypothalamus dataset (see below) led to the same conclusions as analysis of whole brain data. The important point is that we clearly identified some well-described rare cell types, like dopaminergic neurons and infundibular cells, in our amphioxus brain atlas. Therefore, we consider our amphioxus sampling sufficient to identify the major cell populations in the adult amphioxus brain.

Each sample generated more than 350 million total reads, with over 94% passing quality control and sequencing saturation exceeding 87%. STAR uniquely aligned more than 65% reads for all samples. On average, more than 75,000 reads per cell were detected per sample. We also observed a high number of genes (median > 1,000) and transcripts (median > 2,600) captured per cell, along with a low proportion of mitochondrial gene expression (mostly <10%) (Supp. Fig. 1). This shows high and comparable quality compared to vertebrate brain single-cell datasets described in individual references. The amphioxus replicates were also consistent (Supp. Fig. 1A), and major cell types in amphioxus brain were clearly identifiable (Supp. Fig. 1C). These together show a sufficient sampling depth and confirm the high-quality of our amphioxus brain datasets.

Identifying homologous cell types between amphioxus and vertebrates in the hypothalamus

In the search for vertebrate cell-type family homology in amphioxus, there is biological imbalance due to the abundance of cortical cells in the vertebrate brain atlases, which results from the major change in size and complexity of cortex especially in mammals. To find

homologous cell types at finer scales, and hence try and understand homology at different levels, we therefore focused on the hypothalamus—a region considered to be relatively conserved[4,5]. We obtained scRNA data from mouse hypothalamus[6] and used these data for comparative analysis. To balance resolution and interpretability, we adopted the median clustering level "C25" defined by Steuernagel and colleagues in this study[6]. This level captures sufficient cell-type heterogeneity within excitatory and inhibitory neurons (for example, a clear cluster of pro-opiomelanocortin-expressing neurons) without reaching the highest resolution in their study, where the boundaries between cell-type identity and cell state variations can become blurred.

Previous studies have described dopaminergic neurons of vertebrates as "orthologous" or "homologous" to dopaminergic neurons in *Ciona* sensory vesicle[4] or the amphioxus hypothalamo-prethalamic primordium (HyPTh)[7]. However, we think these statements are not substantiated by more detailed analysis of the data now available and explain our reasoning below.

First, dopaminergic neurons in the mouse hypothalamus are diverse. At the mouse atlas clustering level "C25", there are 3 clusters highly expressing *Tyrosine hydroxylase* (*Th*: the marker gene encoding the rate-limiting enzyme in dopamine synthesis). However, only one TF gene (*Foxp2)* and two effectors (*Th* and *Glp1r*) out of approximately 40 marker genes are shared among these 3 clusters (please refer to Supp. Table 5 from Steuernagel and colleagues' study[6]), highlighting the evolutionary diversity of dopaminergic neurons in mouse.

We further identified top TFs for amphioxus dopaminergic neurons, and for *Ciona* coronet cells (which are dopaminergic neurons) in scRNA data from larval CNS[8]. The most specific of these TFs shared between amphioxus and *Ciona* is *Ptf1a* (Supp. Fig. 3) which has also been experimentally validated to label all coronet cells[4] and to regulate terminal differentiation of coronet cells in *Ciona*[9]. This supports homologous relationships between these dopaminergic neurons in amphioxus and *Ciona*. In contrast, *Ptf1a* is not used as a marker (and also not highly expressed) in mouse dopaminergic neurons within the hypothalamus, indicating they are not homologous cell types (though might have a similar function in dopamine release). Interestingly, a *Pvalb+* interneuron cluster (ReInh_89) in the mouse whole brain atlas used *Ptf1a* as a top marker, this could suggest a homologous relationship to amphioxus/*Ciona* dopaminergic neurons, but this would mean a shift in neurotransmitter usage.

To systemically assess homology, we also performed SAMap[10] to down-sampled mouse hypothalamus datasets (containing no more than 1,000 cells per cluster at the level of "C25") to identify transcriptomic similar cells. We found mapping patterns of glial cells between vertebrate hypothalamus and amphioxus adult brain (Supp. Fig. 4) are similar to those between whole adult brain comparisons (Extended Data Figure 2c). We found that amphioxus neuronal cell types (Exc/Inh_0 and Exc/Inh_1) broadly mapped to both excitatory neurons and inhibitory neurons between species, also as we observed in whole brain comparison.

On the contrary, amphioxus Pep_10/13 and Exc_2/Pit_17 that exclusively mapped to mouse C25-7: GLU-7 and C25-23: ParsTuber, respectively, providing better evidence of one-to-one relationships that might indicate homology (Supp. Fig. 4). C25-7: GLU-7 cells are peptidergic neurons highly and specifically expressing *Pmch* (Pro-melanin-concentrating hormone), *Cartpt* (Cocaine and amphetamine regulated transcript), *Tacr3* (Tachykinin receptor 3), *Otx1*, *Igf1*, and *Zic1/4* (see Supp. Table 5 in Steuernagel and colleagues study[6]). However, amphioxus Pep_10 and Pep_13 did not express *Otx* and *Zic*, indicating that while these mappings may capture functional similarity, they differ in their regulation undermining a conclusion of true homology.

C25-23: ParsTuber are pituitary cells that highly and specifically express *Pitx1/2*, *Six1*, *Cga*, and *Tshb* (the latter two are functional genes encode different subunits that form active thyroid-stimulating hormone). However, amphioxus Pit_17 only specifically expresses *Six1/2/4/5* but not *Pitx*. We also observed a small percentage of *Pitx* and *Six1/2/4/5* co-expression in amphioxus Exc/Inh_1 cluster and we further found a subcluster in re-clustering of Exc/Inh_1 with strong co-expression of *Pitx* and *Six1/2/4/5* (Supp. Fig. 5).

Taken together, our analyses across cell-type levels indicate that establishing robust homologous relationships to vertebrate cells at finer levels is challenging. Relying solely on the similarity of a few effector genes is problematic as cells can switch these, as seen by others with neurotransmitter shifts identified in a recent study[11]. It also requires a systematic and phylogenetic evaluation of the conserved key TFs that define cell-type identity. Overall we conclude the proposed homology between amphioxus/*Ciona* dopaminergic neurons and vertebrate dopaminergic neurons is not robustly supported. We also speculate potential homology of vertebrate pituitary cells in amphioxus, although further data and analyses are needed to fully elucidate these possibilities.

Comparison of ohnologue detection methods

We evaluated three methods (doubletrouble[12], DupGene_finder[13], and Ohnologs-v2.0[14]) for detecting ohnologues and found Ohnologs-v2.0 generated more stable and consistent results across vertebrates. We briefly describe each method and summarise results and comparative outcomes below.

Doubletrouble results

Doubletrouble identifies potential in-paralogues by self-BLAST and detects syntenic regions by syntenet (which uses MCScanX). Paralogues that are anchor pairs within syntenic regions are classified as segmental duplicates (or potential ohnologues), and all other duplicates are classified as SSD paralogues. Ohnologue detection in doubletrouble heavily depends on the quality of individual genome assembly and annotation since this method only considers a single species. The reported proportion of ohnologues detected by doubletrouble (in paralogues) varies a lot, from less than 1% to around 75% in vertebrates (shown in Supplementary Text S4 of the doubletrouble study[12]). Only about 200 paralogues in amniotes and 300 paralogues in lamprey were identified as ohnologues with default parameters. Given more flexible parameters (Blast e-value 1e-10, anchors = 3 or 5, max_gaps (number of upstream and downstream genes to search for anchors) = 50 or 100, we found a range of 300-2,000 ohnologues in four vertebrates, which is still far below that detected using Ohnologs-v2.0 and ohnologues derived from Marlétaz and colleagues' study[15]).

DupGen_finder results

DupGen_finder applies similar methods as doubletrouble with consideration of inter-species comparison to distinguish transposed and ancestral duplications. With the default parameters, we identified 94 to 812 pairs of ohnologues, also very far below Ohnologs-v2.0 and Marlétaz and colleagues' results[15].

Ohnologs-v2.0 and comparison between our updates and v2.0

The Ohnologs-v2.0 pipeline utilizes orthologue and paralogue information retrieved from Ensembl BioMart, generated based on gene trees. For vertebrate 1/2R ohnologues (not for teleost 3R), it identifies syntenic blocks and anchors both within individual species and compares to outgroup species without WGD. It also quantifies ohnologue pairs by comparing

other vertebrates and outgroup species with the consideration of phylogenetic sampling and weighting. We used Ohnologs-v2.0 with updated versions of assembly and annotation (Supp. Table 9). Upon examining paralogue information retrieved from Ensembl BioMart (v110), we found many well characterized ohnologues were incorrectly classified as older duplications rather than at the base of Vertebrata. For example, clear ohnologues *Sox8/9/10* and *Lhx6/8* in Ensembl BioMart are identified as duplicated in the Bilateria but are known to be Ohnologue groupings.

We therefore used (In-)paralogues defined in our Methods (duplicated genes in the same orthogroup for each species) as input for Ohnologs-v2.0. We eventually generated relatively stable ohnologue pairs, supported by other vertebrates. For example, 18 out of 21 jawed vertebrates had *Sox8/9/10* identified as ohnologues (please refer to GitHub: https://github.com/DiracZhu1998/WGD2celltype_evolution/tree/main/2.gene_relationships/ohnolog_inferring).

Overall, we identified 5748, 6422, 4390, 3485 pairs of ohnologues and 6206, 6344, 5616, 4273 pooled ohnologues for human, mouse, lizard, and lamprey, respectively. To evaluate the impact of updated genome assemblies and annotations on ohnologue detection, we compared our results and Ohnologs-v2.0 (intermediate parameter settings). More than 75% of human ohnologues identified in our dataset were also present in Ohnologs-v2.0, and vice versa. Similarly, over 70% of mouse ohnologues overlapped between the two datasets. These suggest both versions are largely consistent sets of ohnologues.

Because only the first round of WGD is shared between jawless and jawed vertebrates, we assessed the impact of including or not including lamprey in our ohnologue detection pipeline. We found the resultant ohnologue differences were very small, with less than 0.2% differences in human, mouse, and lizard. These results indicate that the presence of lamprey sequences has negligible effects on ohnologue identification by the Ohnologs-v2.0 pipeline in these species.

Comparison of our ohnologue set with that of and Marlétaz and colleagues

Human, mouse, and lizard ohnologues were not specifically identified in Marlétaz and colleagues' study[15]. However, over 70% of ohnologues in human, mouse, and lizard in our list were identified as ohnologues in Marlétaz and colleagues' chicken ohnologue set (https://github.com/fmarletaz/hagfish/blob/main/Paralogons/Vert_Evt_OGrrA.txt), as judged by comparison following conversion of gene IDs from our species to chicken IDs with reciprocal best hit (RBH) analysis. Conversely, there were slightly more ohnologues detected

in Marlétaz and colleagues' results than ours, and around 45% of chicken ohnologues were identified as ohnologues in our human, mouse, and lizard datasets. Considering the number of RBH (~13,000) between chicken and human/mouse/lizard, we think these overlaps are acceptable.

The only species shared between our research and Marlétaz and colleagues' research is the sea lamprey (*Petromyzon marinus*). The lamprey gene IDs listed in Marlétaz and colleagues' results were found in a previous assembly kPetMar1.pri (GCF_010993605.1) and latest assembly UKy_Petmar_22M1.pri1.0 (GCF_048934315.1). Instead, we used the same assembly and annotation as Lamanna and colleagues' lamprey brain scRNA-seq study[16] which was based on the Pmar_germline 1.0 (GCA_002833325.1) assembly and updated annotation. Only 12,536 and 12,760 RBHs were identified between protein-coding genes from our version (Pmar_germline 1.0) and kPetMar1.pri or UKy_Petmar_22M1.pri1.0, respectively. Around 60% of protein-coding genes in kPetMar1.pri or UKy_Petmar_22M1.pri1.0 had RBHs in our version (Pmar_germline 1.0). This low overlap of gene sets for the same species hindered downstream ohnologue comparison between Ohnologs-v2.0 and Marlétaz and colleagues' methods.

We identified 3,485 ohnologue pairs and 4,273 pooled ohnologues for lamprey (Pmar_germline 1.0), while Marlétaz and colleagues' identified 6,166 pooled ohnologues. Only 45% of lamprey ohnologous identified in our datasets were recognized as ohnologues in Marlétaz and colleagues' results, and 37% vice versa, under the circumstances of only about half of total coding genes finding their RBH.

Despite this, however, we showed that our conclusions hold true when we convert Marlétaz and colleagues' ohnologue results and NCBI IDs to the gene ID version used in single-cell lamprey atlas (Fig. 2b). We hence think the difference has limited influence on our conclusions, but we recommend that future studies evaluate and refine the gene annotation of lamprey to improve its quality.

WGD vs SSD at additional levels

To understand WGD and SSD differences at additional levels, we examined coding sequences, protein-protein interactions (PPIs), and regulatory motifs.

We calculated Ka/Ks ratios for both WGD and SSD paralogue pairs and found that most ratios were below 1, indicating overall purifying selection across both categories. Comparison of Ka/Ks ratios between WGD and SSD paralogues revealed that ohnologues are subject to stronger purifying selection than SSD paralogues in all four vertebrate species (Supplementary Fig. 18).

We downloaded human and mouse protein-protein interaction data (PPIs) from the STRING database (v12.0; https://string-db.org)[17] and found that ohnologues were significantly more likely to participate in PPIs, whereas SSD paralogues were less likely to do so (Fisher's exact test: odds ratio ≈ 2, $P < 2.2e-16$ for both human and mouse ohnologues; odds ratio ≈ 0.7, $P < 2.2e-16$ for both human and mouse SSD paralogues). These findings are consistent with the gene balance hypothesis[18], which posits that genes encoding components of protein complexes are preferentially retained following WGD due to stoichiometric sensitivity. Moreover, ohnologous TFs regulated significantly more target genes than SSD-derived TFs (Supplementary Fig. 19), supporting the idea that dosage balance extends beyond protein complexes to encompass GRNs[18].

Ohnologous TFs initially share target gene sets following WGD but subsequently diverge in regulatory function. We analysed three regulatory motif shapes (Supplementary Fig. 20) using the approach of Almeida-Silva et al[19]. After excluding self-regulation, the human GRN contained 446 TFs and 13,043 targets, and the mouse GRN 390 TFs and 10,761 targets in total (in our datasets). All motifs were significantly enriched among either ohnologues or SSD paralogues, except the Bifan motif in SSD paralogues. Notably, all three motifs showed stronger enrichment among ohnologues, indicating that WGD-derived TFs have a higher contribution to new regulatory motifs. The most obvious difference between WGD and SSD paralogues on motif enrichment was the V-shaped motif, which was markedly enriched among ohnologues (Supplementary Fig. 20), suggesting greater conservation of regulatory relationships between TFs and target genes derived by WGD.

Comparative analysis between vertebrate and amphioxus regarding regional identity programmes

Brain regionalisation occurs early in vertebrate development and the transcriptomic differences across brain regions correspond to progenitor cell position, which has lasting effects on cell specification and differentiation[20–22]. A recent study suggests that neurons and astrocytes in the same region share some region-specific markers[23]. This is why we compared astrocytes and neurons to identify shared regional identity programmes in main text.

As described in main text, we identified some key TFs in vertebrate brain development and specification, including *Foxg1*, *Emx1*, *Fezf2*, and *Prox1* (telencephalon)[24–27]; *Tcf7l2*, and *Six3* (diencephalon)[28,29]; *Otx2*, *En1/2,* and *Pax7* (mesencephalon)[30–32]; *En1/2*, *Pax3*, *Zic1/2*, and *Neurod1/2* (rhombencephalon)[31,33,34].

To understand amphioxus CNS regionalisation and compare it with vertebrates, we analysed adult bulk RNA-seq data[35], identifying TFs with strong anterior-posterior regional expression (Supplementary Fig. 11). Many correspond to embryonic TFs known to regulate regionalisation[5], and some (e.g., *Arx*, *Fezf*, *Lhx2/9*, *Shox2*, *Hox3*) showed conserved expression in vertebrates (Supplementary Fig. 12-17). However, none of vertebrate regional TFs mentioned above were in the amphioxus regional TFs, except *Fezf* (Extended Data Fig. 10c). This suggests regional identities of major cell-type families partly originated in the vertebrate ancestor, with some regulatory programmes conserved across chordates.

Supplementary Figures

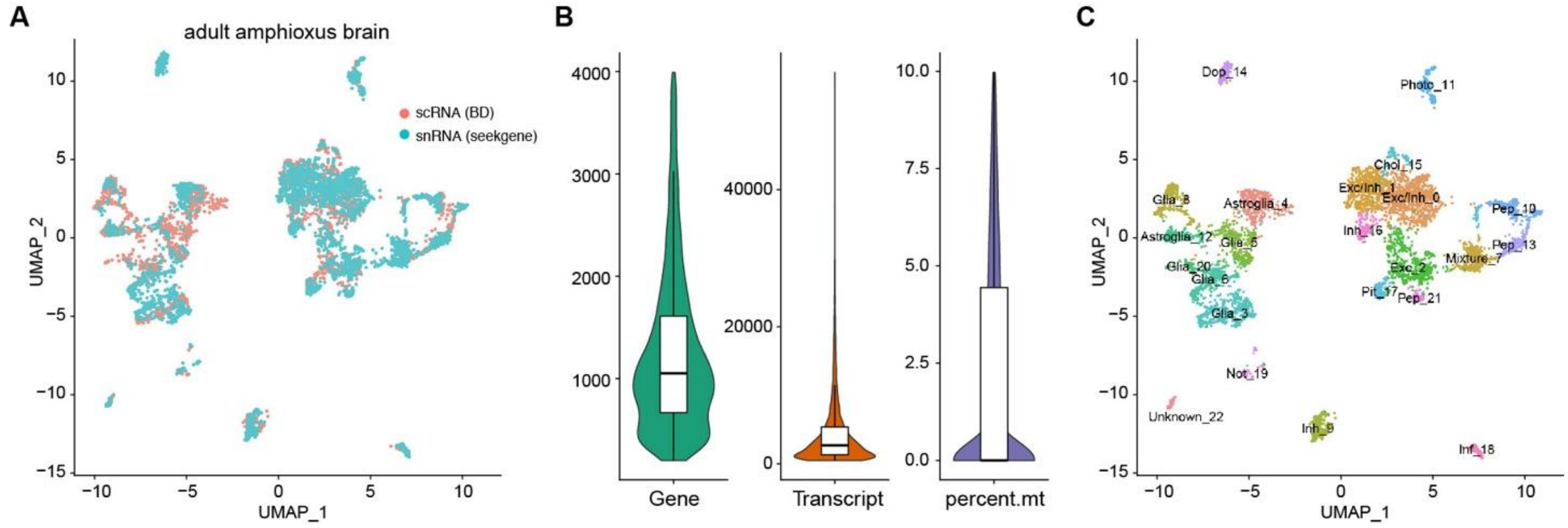

Supplementary Fig. 1 Amphioxus brain atlas and quality. (A) UMAP visualization of the integrated adult amphioxus brain single-cell atlas. Two colours represent the two biological replicates, generated by scRNA-seq and snRNA-seq. (**B)** Violin plots showing the distribution of the number of detected genes, transcripts (UMI), and the percentage of mitochondrial transcripts expression per cell. The width represents the probability density. The central line indicates the median, and the box denotes the interquartile range. (**C)** UMAP visualization of identified adult amphioxus brain atlas with cell-type annotation.

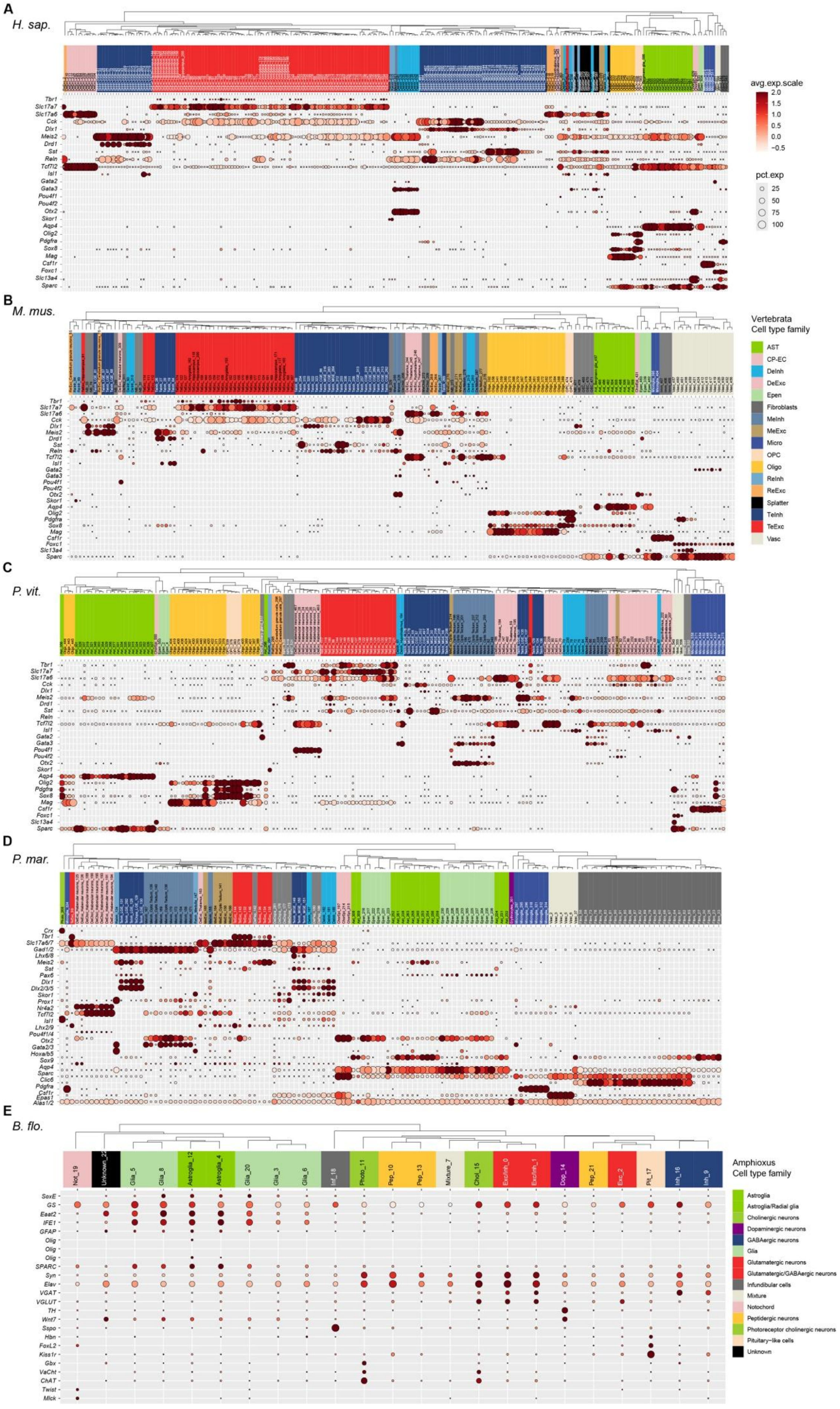
A
H. sap.
avg.exp.scale
2.0
1.5
1.0
0.5
0.0
−0.5
pct.exp
25
50
75
100
B
M. mus.
Vertebrata
Cell type family
AST
CP-EC
DeInh
DeExc
Epen
Fibroblasts
MeInh
MeExc
Micro
OPC
Oligo
ReInh
ReExc
Splatter
TeInh
TeExc
Vasc
C
P. vit.
D
P. mar.
E
B. flo.
Not_19
Unknown_22
Glia_5
Glia_8
Astroglia_12
Astroglia_4
Glia_20
Glia_3
Glia_6
Inf_18
Photo_11
Pep_10
Pep_13
Mixture_7
Chol_15
ExcInh_0
ExcInh_1
Dop_14
Pep_21
Exc_2
Pit_17
Inh_16
Inh_9
Amphioxus
Cell type family
Astroglia
Astroglia/Radial glia
Cholinergic neurons
Dopaminergic neurons
GABAergic neurons
Glia
Glutamatergic neurons
Glutamatergic/GABAergic neurons
Infundibular cells
Mixture
Notochord
Peptidergic neurons
Photoreceptor cholinergic neurons
Pituitary-like cells
Unknown

Supplementary Fig. 2 Expression of markers and identification of clusters in vertebrates. (A-E) Dot plots showing canonical marker genes at the cluster level, generated from iterative clustering for four vertebrates – human (**A**), mouse (**B**), lizard (**C**), and lamprey (**D**) – and from Seurat Louvain clustering for amphioxus (**E**). A subset of markers (see details in Supplementary Table 1) is shown. Dot size indicates the percentages of cells within each cluster expressing that gene (pct. exp.), while dot colour reflects the scaled average expression (−2 to 2) across all clusters. Dots representing clusters with <1% of cells expressing the gene were omitted to reduce noise. The dendrogram was calculated as follows: (1) calculating the average unique molecular identifier (UMI) counts of marker genes at the cluster level; (2) removing the top and bottom 10% of highly and lowly expressed markers to reduce bias; and (3) constructing the tree using pvclust[36] with 100 bootstrap (nboot=100) and parameters method.hclust = "average", method.dist = function(z){as.dist(1-cor(z,use="pa",method="spearman"). For gene families with multiple copies in lamprey, only the copy with the highest expression was shown. For example, among the three *Tbr1* subfamily copies in lamprey, two were expressed in TeExc cells, but only *MSTRG.6042* is displayed.

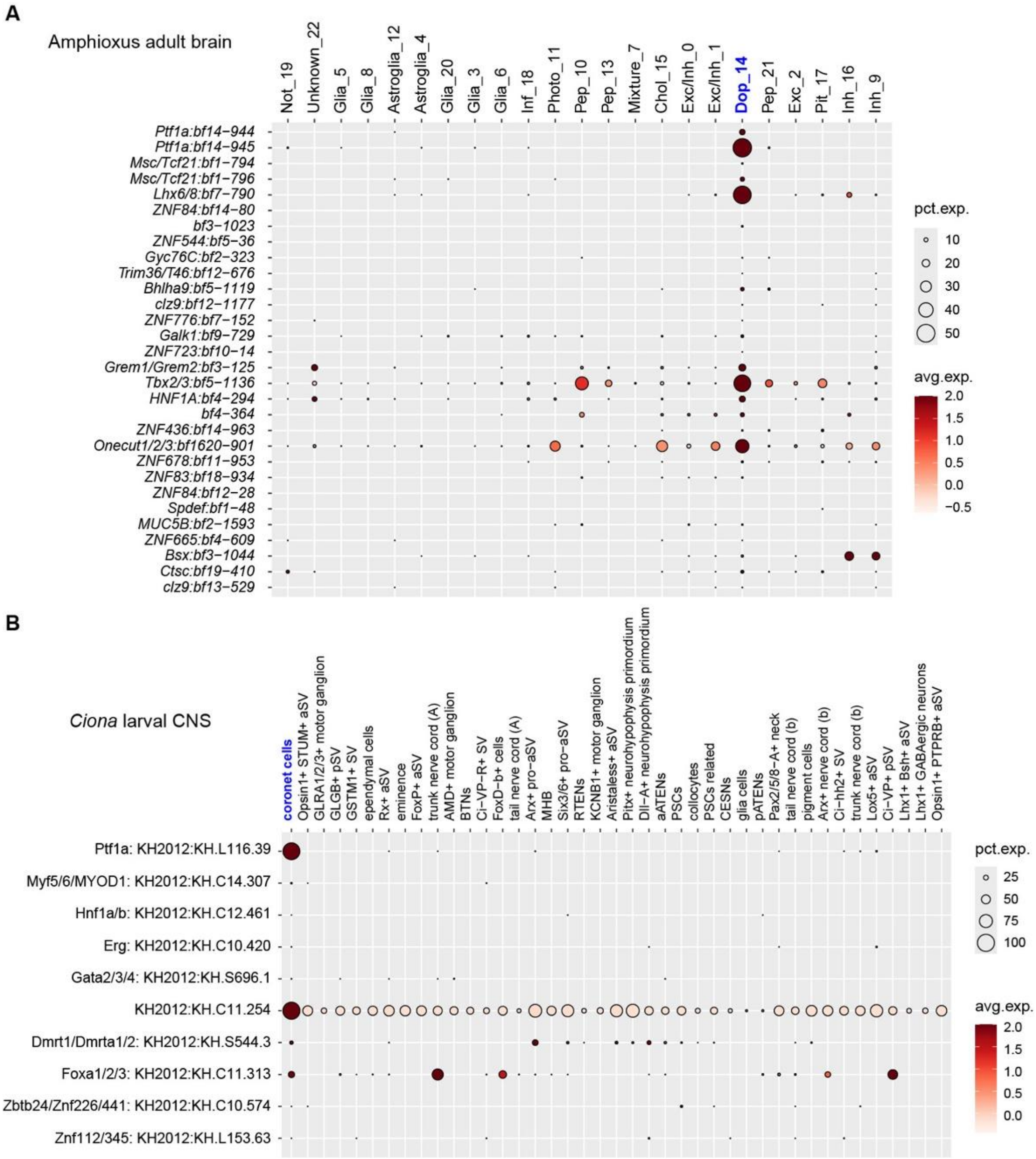

Supplementary Fig. 3 Top TF genes of dopaminergic neurons. (A, B) Dot plots showing top specific TF genes of dopaminergic neurons in adult amphioxus brain (**A**) and *Ciona* larval CNS (**B**). Dopaminergic neuron clusters are highlighted in blue. Top specific TF genes were predicted using the nsforesting.NSForest function as in methods, with fewer TFs considered (n_top_genes = 10, n_binary_genes = 10) for *Ciona*. This method enables the identification of highly specific genes, including those expressed in only a small fraction of cells. Dot size represents the percentage of cells within each cluster expressing that gene (pct. exp.). The colour gradient for each dot is scaled average expression for each gene within the species.

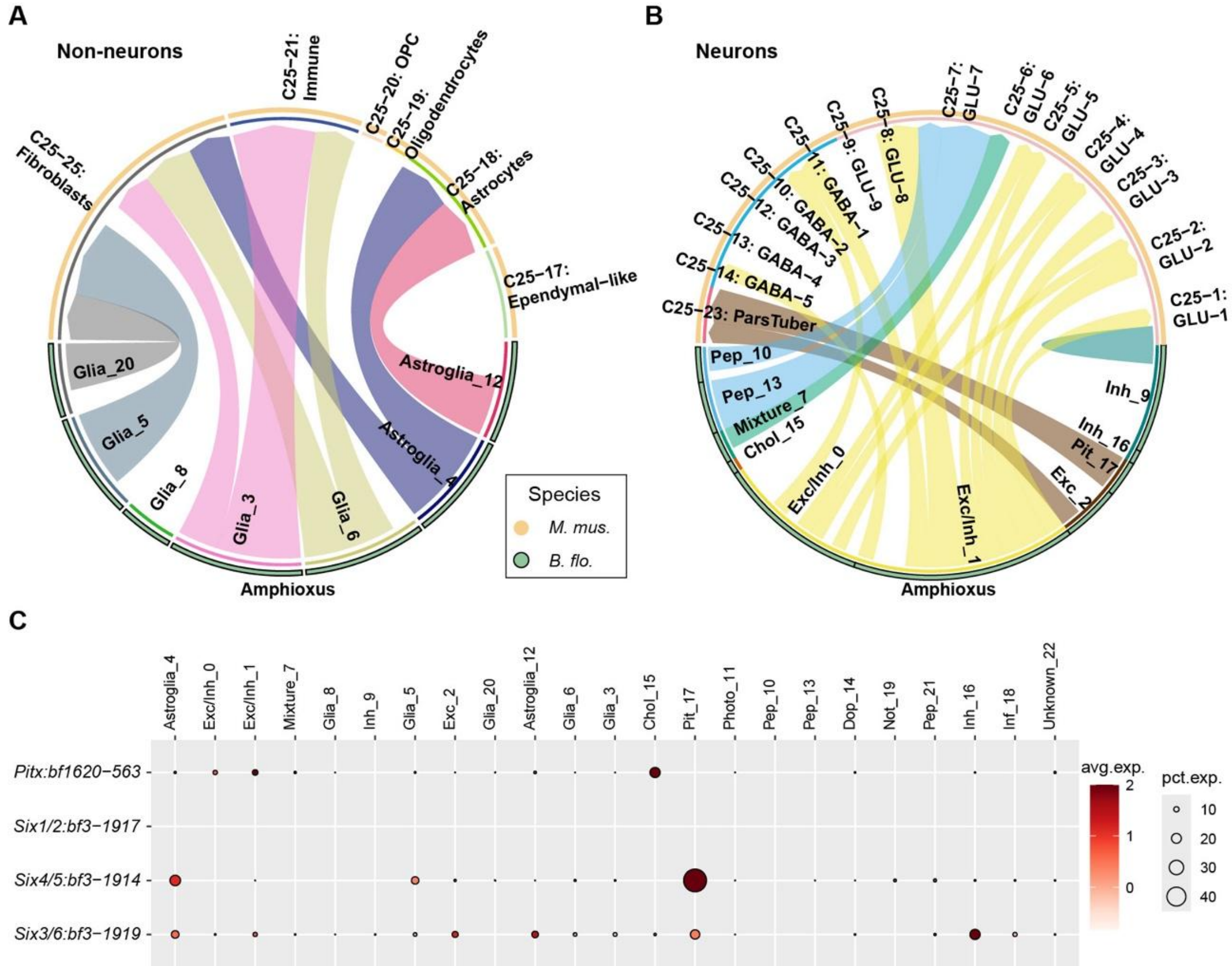

Supplementary Fig. 4 Mapping between adult amphioxus brain and mouse hypothalamus. (A, B) Chord plots showing mappings within non-neuronal and neuronal cell types from adult amphioxus brain to mouse hypothalamus, respectively. The outer coloured circle represents different species. The inner coloured circle denotes the colour of major cell-type families in vertebrates and cell types in amphioxus. Major cell-type families of vertebrates and cell types of amphioxus were also bold labelled with their abbreviations. **(C)** Dot plot showing gene homologs of two key TFs of mouse C25-23: ParsTuber pituitary cells in amphioxus. Dot size represents the percentages of cells within each cluster expressing that gene (pct.exp.). The colour gradient for each dot is scaled average expression for each gene within the species.

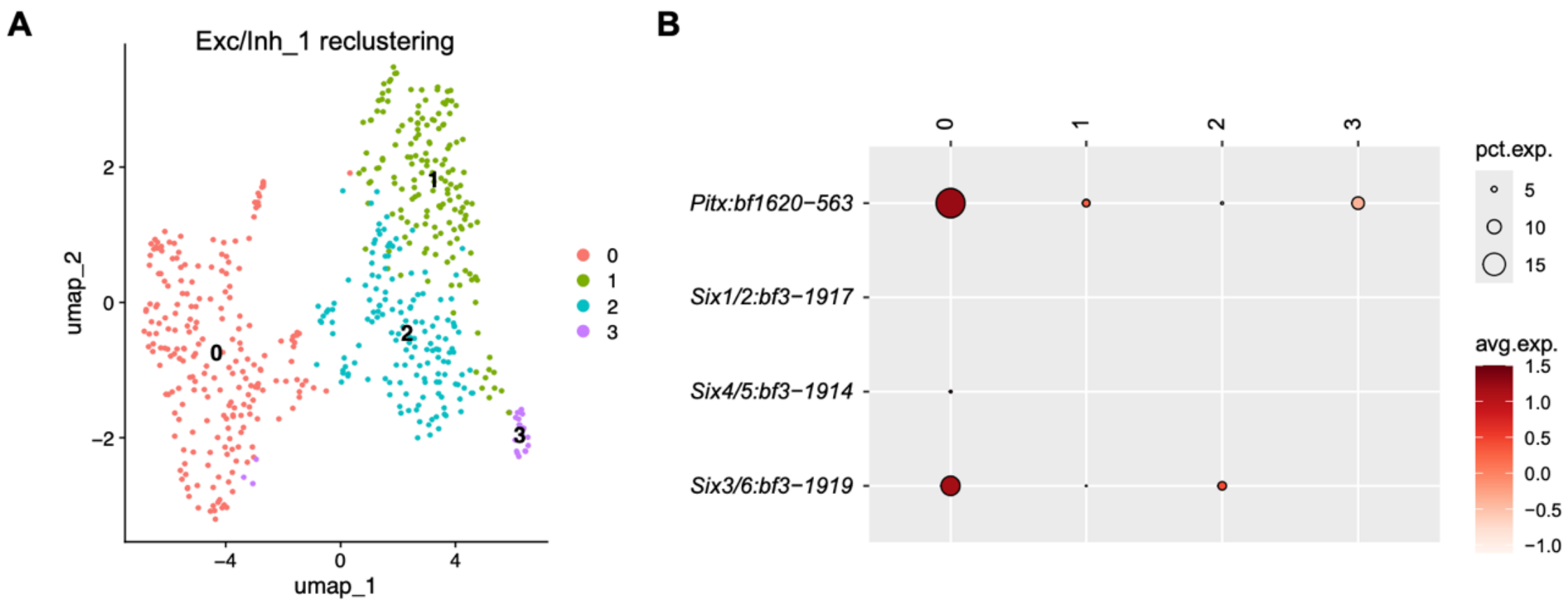

Supplementary Fig. 5 Potential homology of C25-23: ParsTuber pituitary cells in amphioxus. (A) UMAP visualisation of re-clustered Exc/Inh_1 cluster of amphioxus adult brain. **(B)** Dot plot showing gene homologs of two key TFs of mouse C25-23: ParsTuber pituitary cells in amphioxus sub-clusters of Exc/Inh_1. Dot size represents the percentages of cells within each cluster expressing that gene (pct. exp.). The colour gradient for each dot is scaled average expression for each gene within the species.

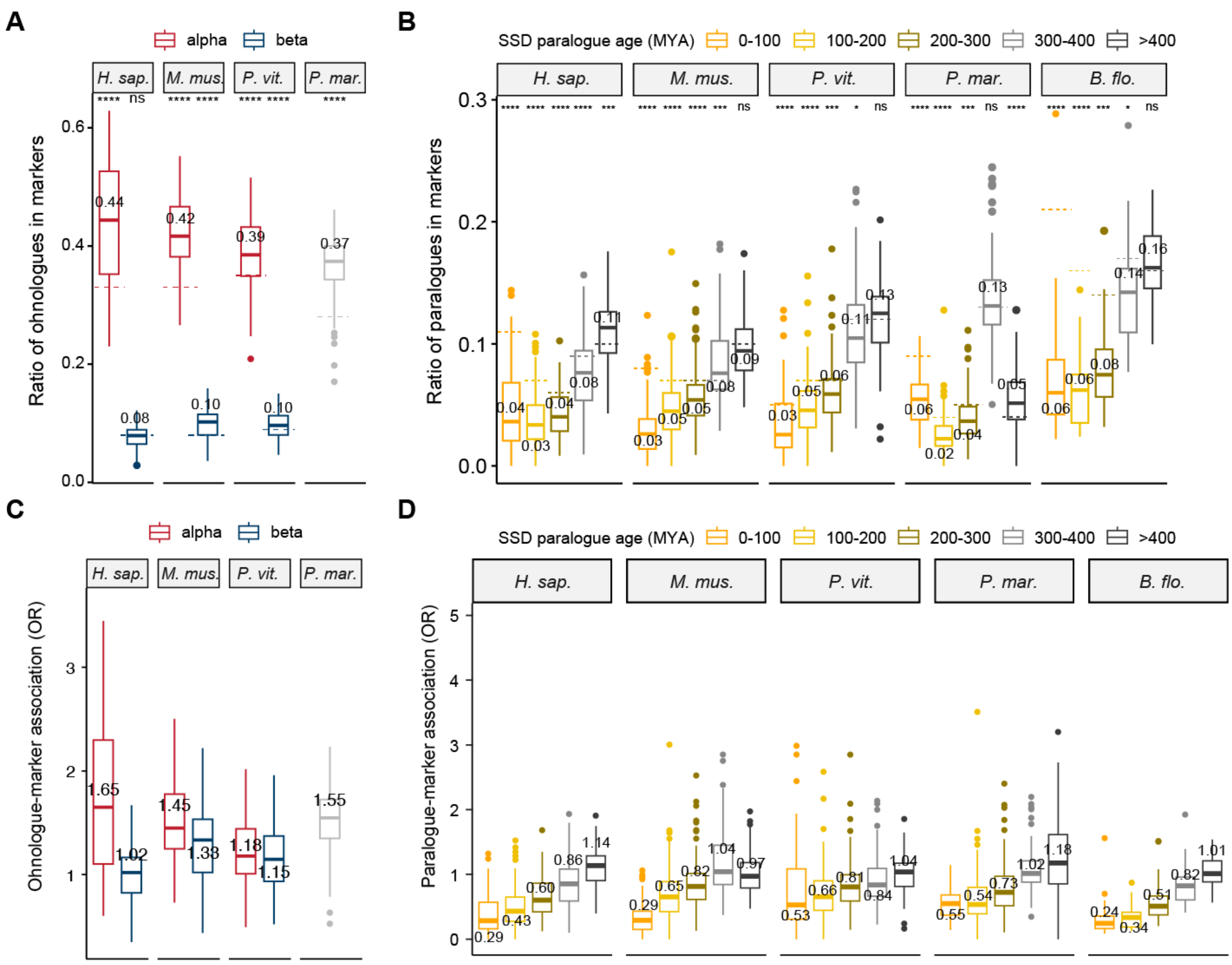

Supplementary Fig. 6 Paralogous gene association with cell cluster markers. (A) Box plots displaying the ratio of 'alpha' and 'beta' ohnologues among cell-type (cluster level listed in Supplementary Table 2) DEGs. The significance of differences between the ratio of paralogues as markers and their total percentage in all protein-coding genes (background) was assessed using one-sample Wilcoxon signed-rank test (two-sided). Background values are indicated by dotted lines: red for 'alpha' and dark blue for 'beta' ohnologues. Lamprey ohnologues, which were not subdivided due to extensive gene loss in cyclostomes, are shown as a grey box plot with a corresponding grey dotted background line. (**B**) Box plots showing the ratio of age stratified SSD paralogues in cell-type (cluster level) DEGs. Background and significance were calculated in the same way as (**A**). Only cell types with more than 100 DEGs were included to ensure robust statistical comparisons, as some clusters with fewer (for example 100) cells yield limited numbers of detectable DEGs and even fewer when considering them with age stratified gene sets. **(C)** Odds ratios (ORs) calculated from two-sided Fisher's exact test on 'alpha' and 'beta' ohnologues with cell-type (cluster level) DEGs. (**D)** ORs calculated from two-sided Fisher's exact test on age stratified SSD paralogues with cell-type (cluster level) DEGs. Only cell types with more than 100 DEGs were included to ensure robust statistical comparisons, as the same reasons described in (**B**). (**A,B**) Two-sided one-sample Wilcoxon signed-rank test, *p*-value: ns (not significant); * ($P < 0.05$); ** ($P < 0.01$); *** ($P < 0.001$); **** ($P < 0.0001$). Boxplot elements as Fig. 2a.

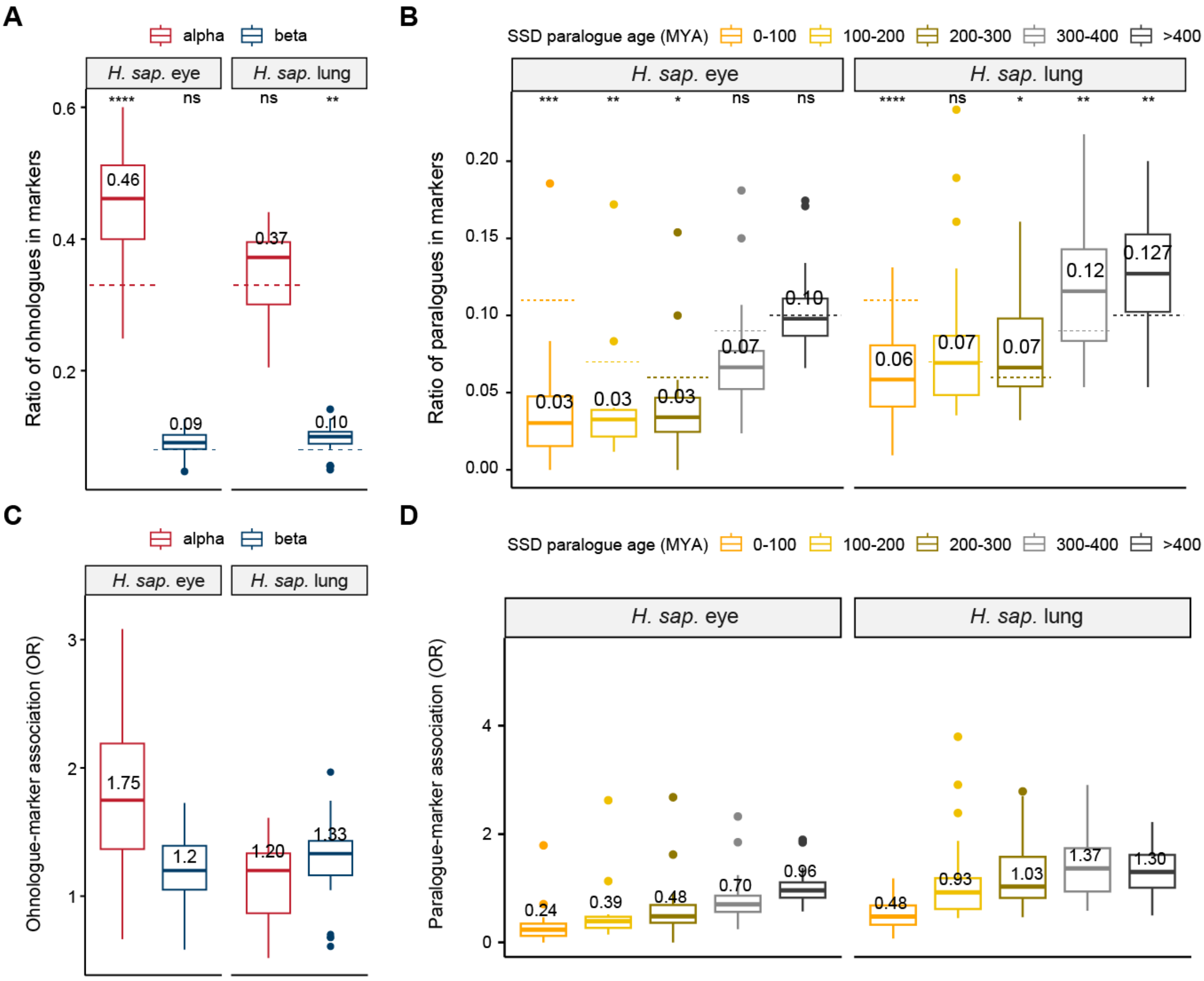

Supplementary Fig. 7 Paralogous genes association with cell-type markers from human eye and lung. (A) Box plots displaying the ratio of 'alpha' and 'beta' ohnologues among cell-type DEGs. The significance of differences between the ratio of paralogues as markers and their total percentage in all protein-coding genes (background) was assessed using one-sample Wilcoxon signed-rank test (two-sided). Background values are indicated by dotted lines: red for 'alpha' and dark blue for 'beta' ohnologues. (**B**) Box plots showing the ratio of age stratified SSD paralogues in cell-type DEGs. Background and significance were calculated in the same way as (**A**). **(C)** Odds ratios (ORs) calculated from two-sided Fisher's exact test on 'alpha' and 'beta' ohnologues with cell-type DEGs. **(D)** ORs calculated from two-sided Fisher's exact test on age stratified SSD paralogues with cell-type DEGs of human eye and lung. (**A,B**) Two-sided one-sample Wilcoxon signed-rank test, p-value: ns (not significant); * ($P < 0.05$); ** ($P < 0.01$); *** ($P < 0.001$); **** ($P < 0.0001$). Boxplot elements as Fig. 2a.

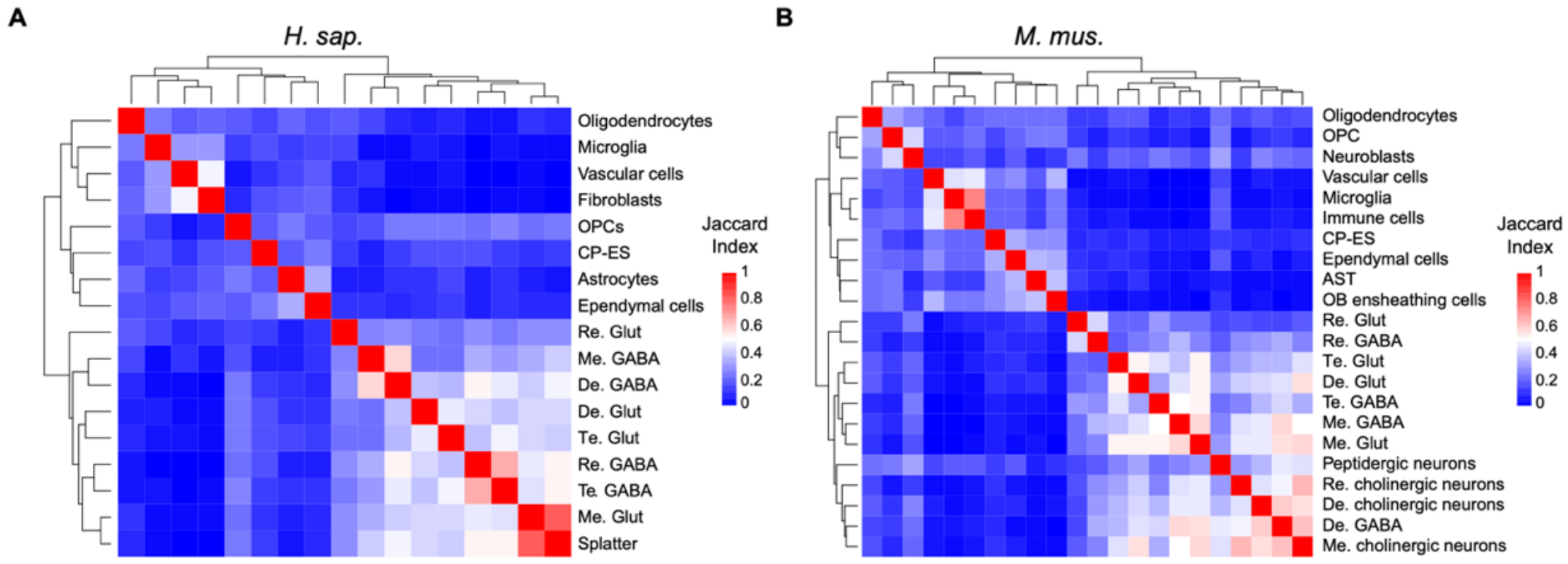

Supplementary Fig. 8 Regulon similarity of human and mouse.

(A,B) Heatmap showing the Jaccard index of the TFs of top 100 regulons across brain cell-type families within human (**A**) and mouse (**B**), respectively. The colour represents the value of Jaccard index, calculated by:

$$J(A, B) = \frac{|A \cap B|}{|A \cup B|}$$

The total number of active regulons in human and mouse (in our datasets) were 446 and 390, respectively.

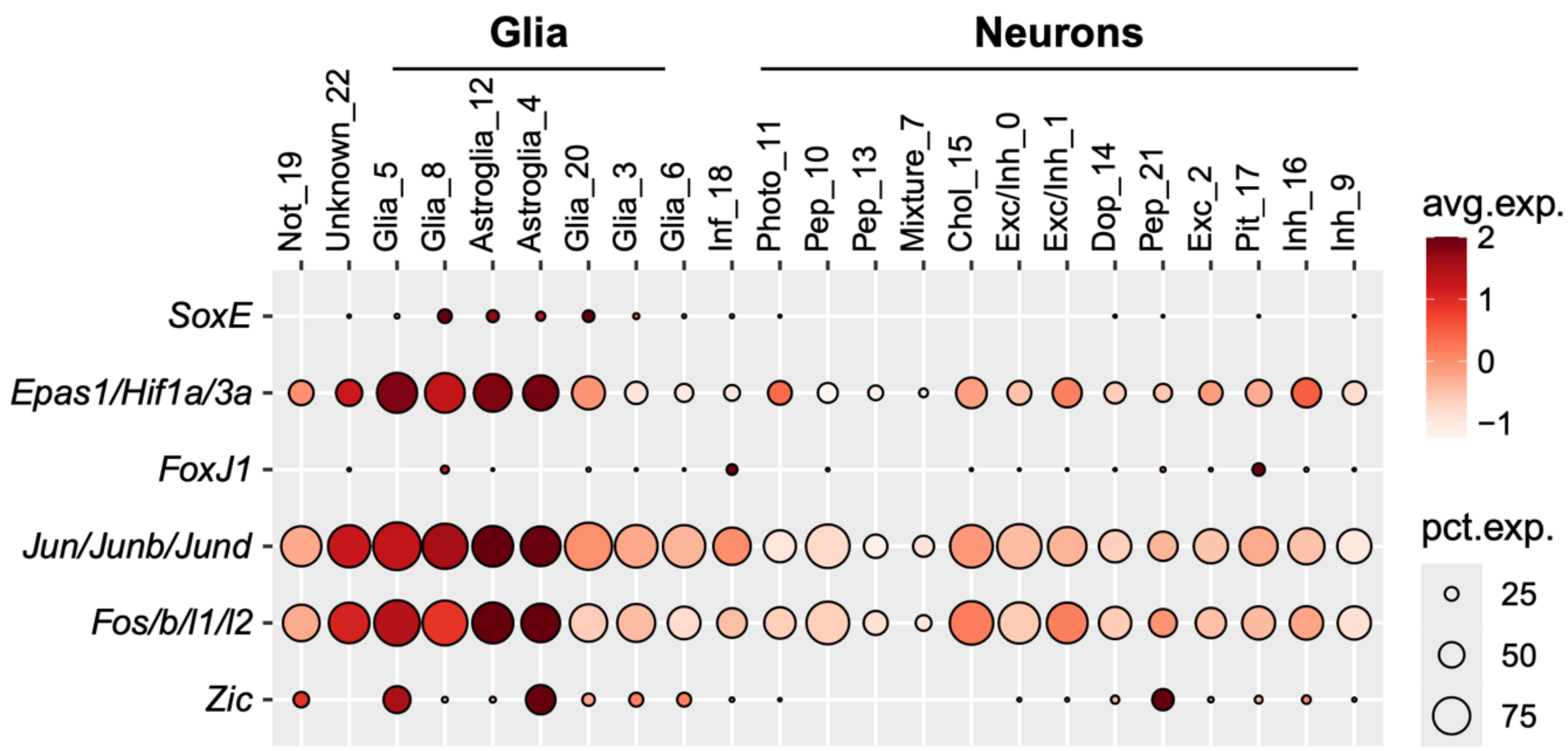

Supplementary Fig. 9 Key TFs between astrocytes and ependymal cells in adult amphioxus brain. Dot plot showing the expression of gene homologs of key TFs between astrocyte and ependymal cell in amphioxus clusters. Dot size represents the percentage of cells expressing each gene within a cluster, and colour denotes scaled average expression. Of the six conserved orthogroups, five have identifiable gene homologs in amphioxus. *SoxE (Sox8/9/10)* is not differentially expressed between astrocyte and ependymal cells, but *Sox9* is highly expressed in both; therefore, SoxE is part of key CoRCs and is also shown.

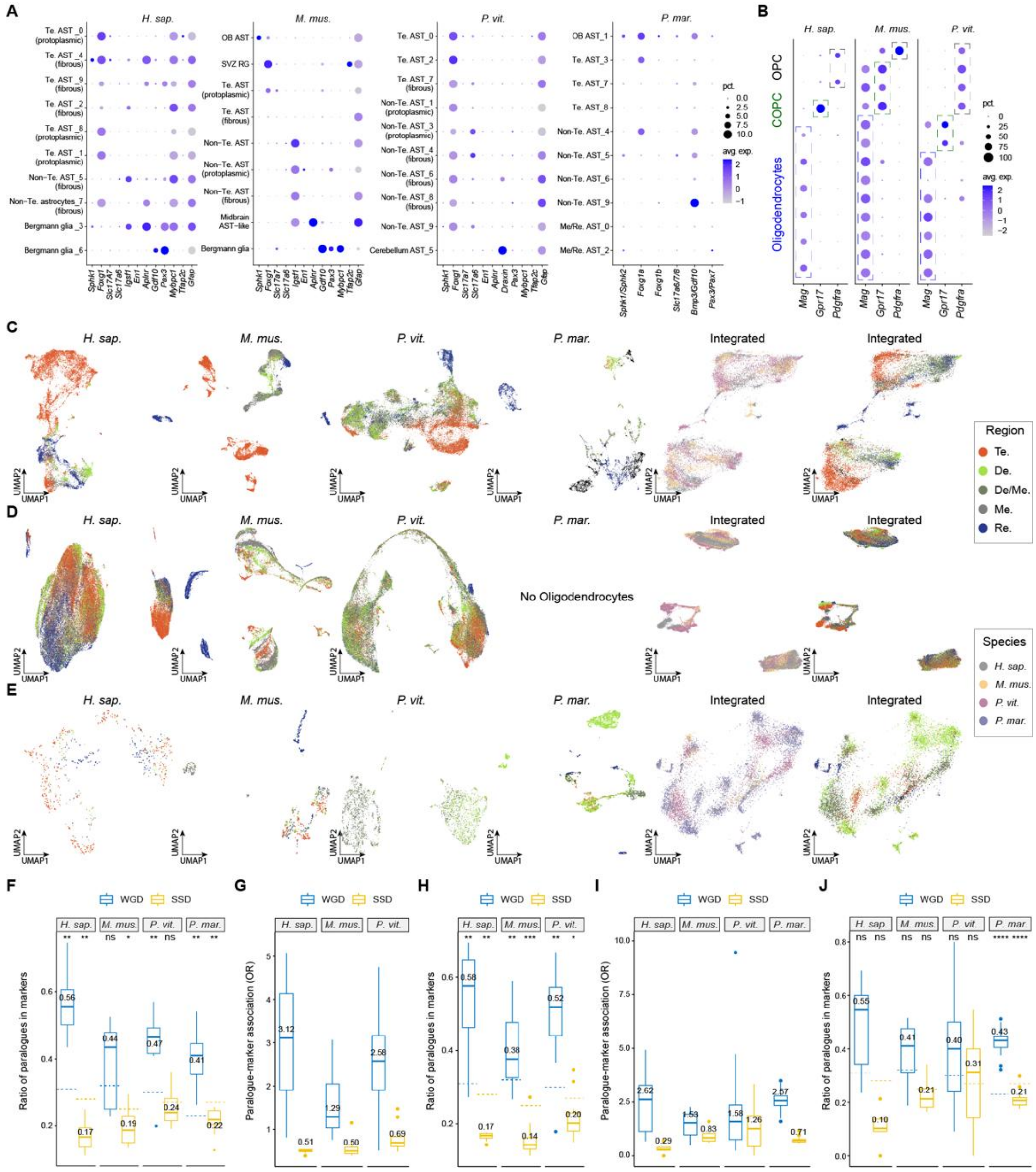

Supplementary Fig. 10 Macroglia diversity and marker relationship with paralogues. (**A**) Dot plots showing astrocyte marker expression across vertebrates. Colour indicates scaled average expression from -2 to 2, while dot size represents the percentage of expressing cells (*capped* at 10%). Abbreviations: OB, Olfactory bulb; SVZ, Subventricular zone; RG, Radial glia. (**B**) Dot plot showing the expression of oligodendrocyte markers, with the same scaling and percentage representation as in (**A**) but not capped. Abbreviation: COPC, Committed oligodendrocyte precursor cells. (**C-E)** UMAP plots of individual and integrated atlases for astrocytes (**C**), oligodendrocytes (**D**), and ependymal cells (**E**). Colours indicate dissection location and species, as detailed in the legend on the right. (**F,H,J)** Proportion of ohnologues (WGD, blue) and SSD paralogues (SSD, yellow) among DEGs in the subtype of astrocytes (**F**), oligodendrocytes (**H**), and ependymal cells (**J**). Background proportions of ohnologues and SSD paralogues in expressed protein-coding genes are indicated by blue and yellow dotted lines, respectively. Significance was evaluated by one-sample Wilcoxon signed-rank test (two-sided). ns (not significant); * ($P < 0.05$); ** ($P < 0.01$); *** ($P < 0.001$); **** ($P < 0.0001$). (**G,I**) Odds ratios (ORs) from Fisher's exact test assessing enrichment of ohnologues and SSD paralogues among DEGs in oligodendrocytes (**G**) and ependymal cells (**I**).

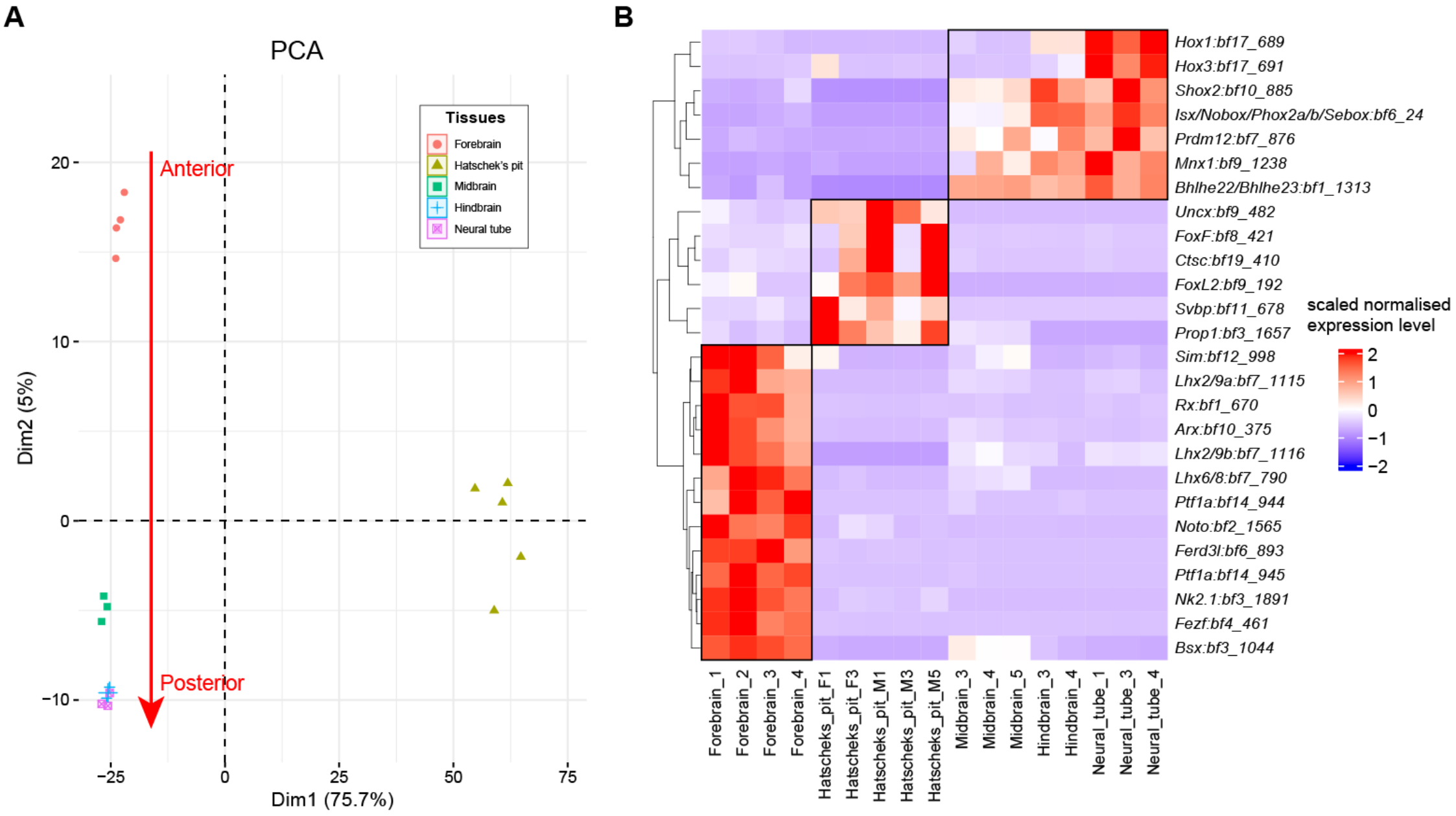

Supplementary Fig. 11 Regional patterning in adult central nervous system of amphioxus. (A) Principal-component analysis was performed based on the top 2,000 highly variable genes scaled DESeq2[37] normalised expression, showing dimension 1 (Dim1) effectively separates CNS tissues vs. Hatschek's pit and dimension 2 (Dim2) captures variance from anterior to posterior axis (A-P axis). Due to limited studies on adult CNS and regional patterning, the authors of this study[7] dissected brain and the rest of neural tube based on the first dorsal ocellus, as we did in this research. The brain was evenly divided into three regions along the A-P axis: forebrain, midbrain, and hindbrain. Please refer to Figure S1A from this study[7] for detailed locations of dissection. This division may not be optimal, as the developing Di-Mesencephalic primordium (DiMes) represents a small, poorly characterized region in adults that likely falls within either the forebrain or hindbrain in their dissections. (**B**) Heatmap showing potential TFs with strong regional identity at amphioxus adult central nervous system, retrieved from top 10% loading genes of Dim2 (genes highly contributing to A-P axis variances).

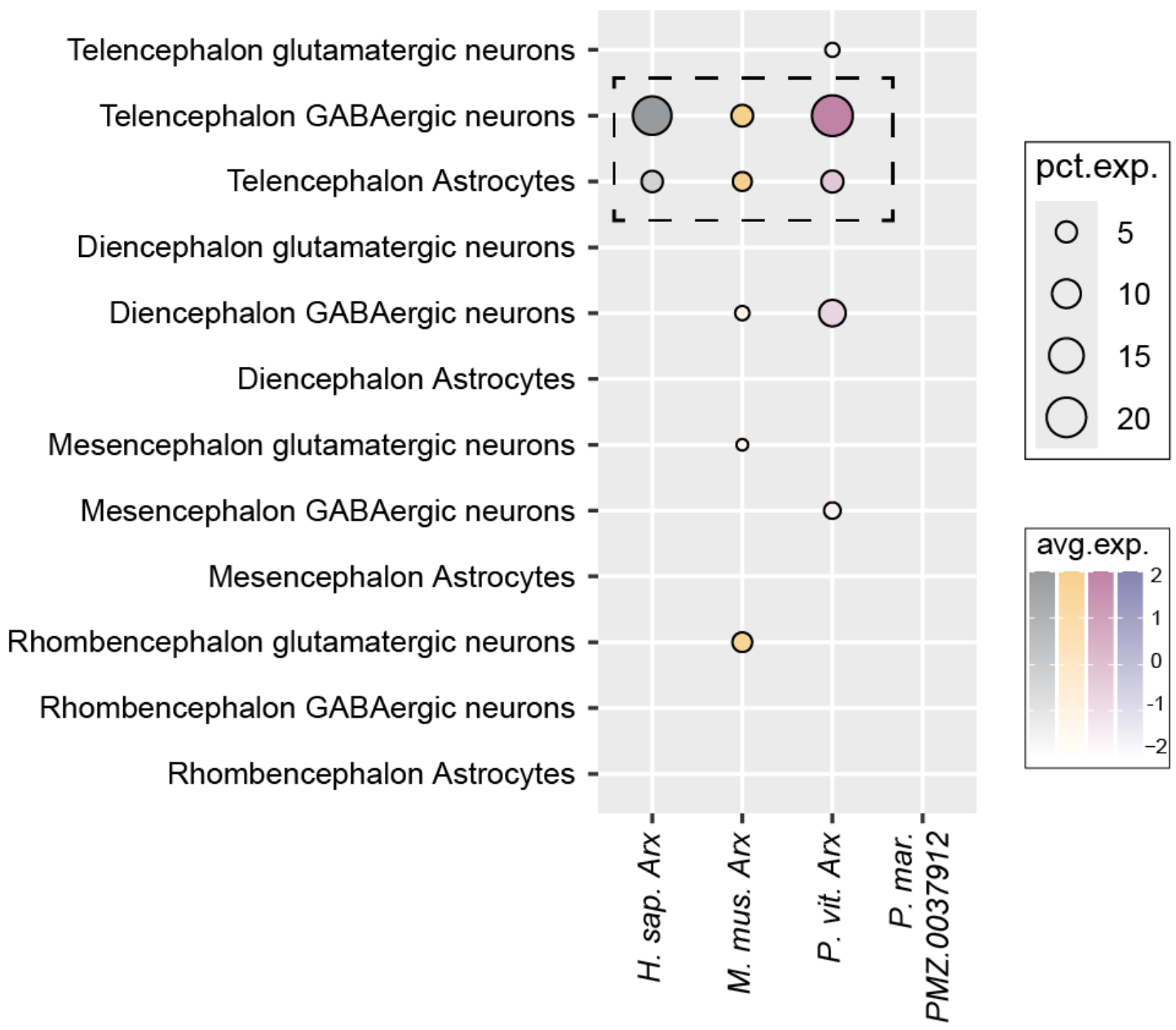

Supplementary Fig. 12 Dot plot showing *Arx* expression in vertebrates. Dot size represents the percentages of cells within each cluster expressing that gene. The gradient colours from white to species colour were scaled for each gene on individual species.

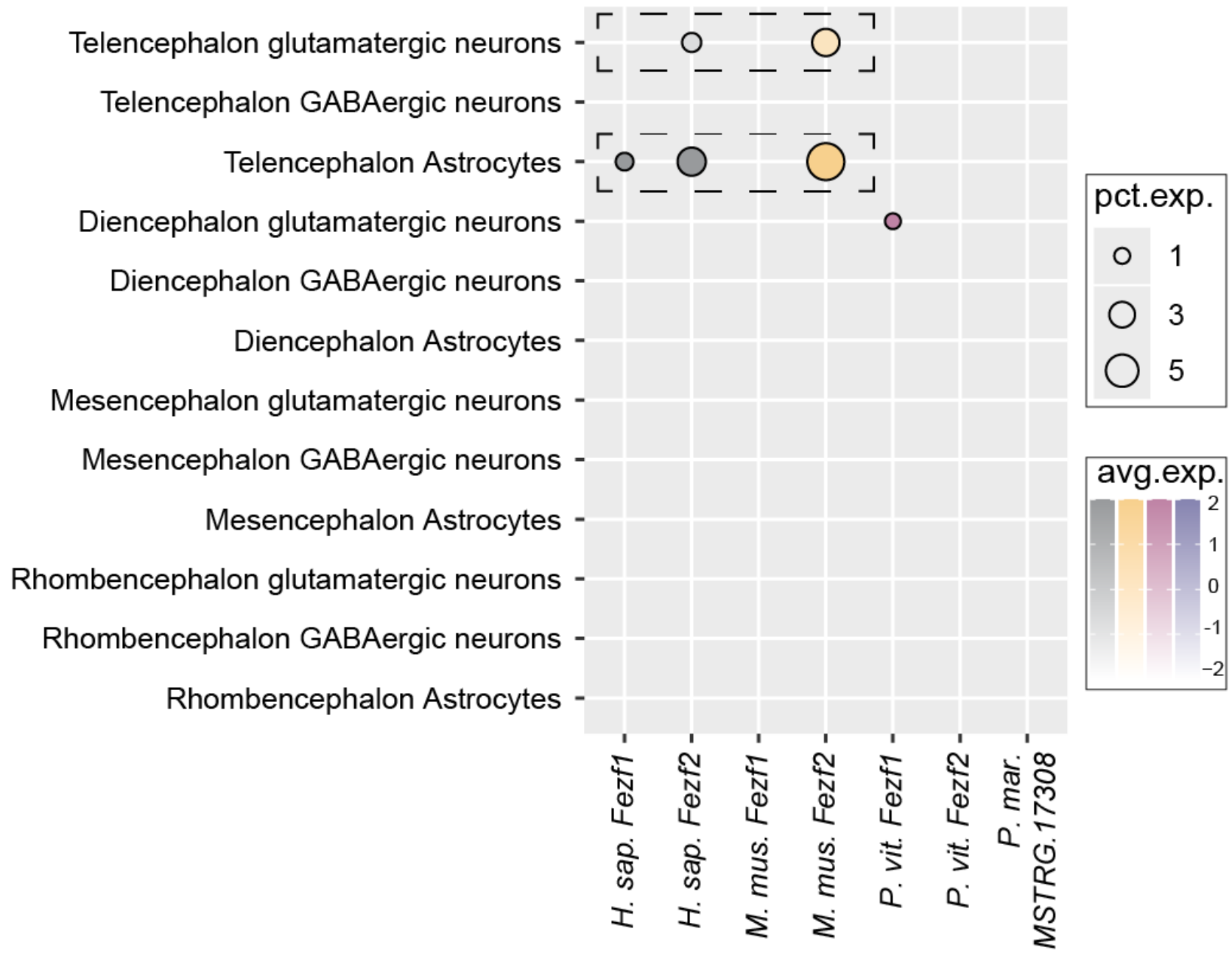

Supplementary Fig. 13 Dot plot showing *Fezf* expression in vertebrates. Dot size represents the percentages of cells within each cluster expressing that gene. The gradient colours from white to species colour were scaled for each gene on individual species.

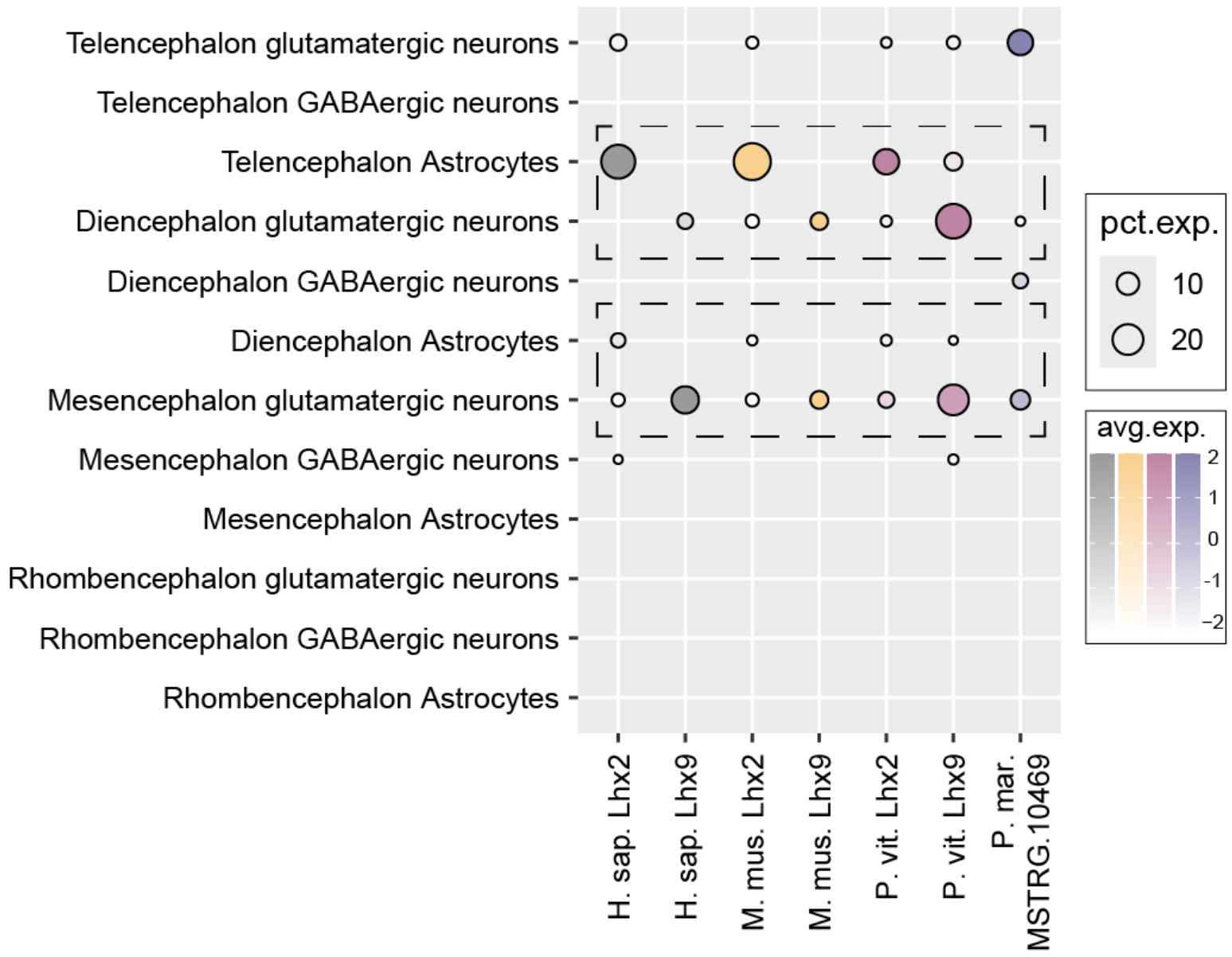

Supplementary Fig. 14 Dot plot showing *Lhx2/9* expression in vertebrates. Dot size represents the percentages of cells within each cluster expressing that gene. The gradient colours from white to species colour were scaled for each gene on individual species.

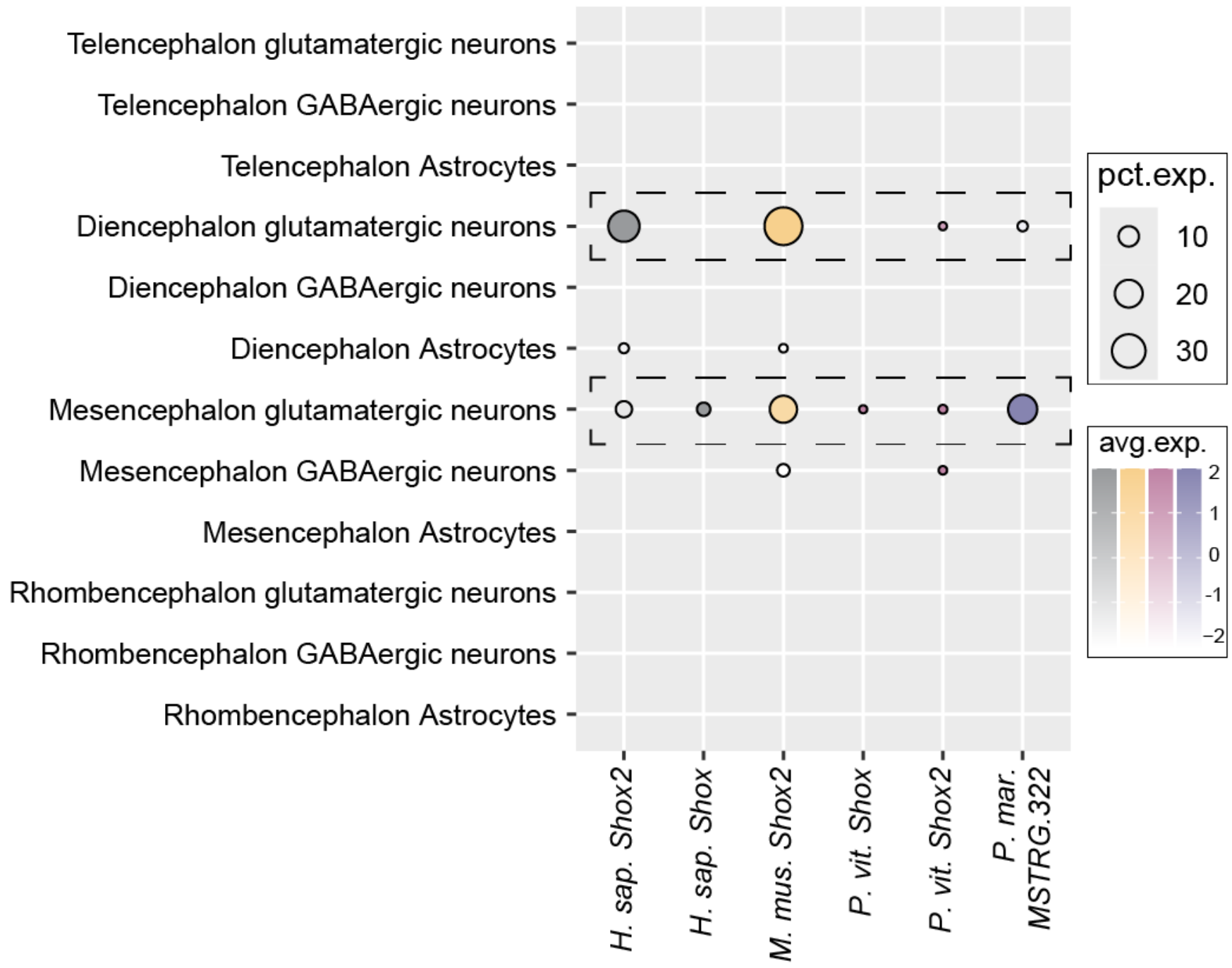

Supplementary Fig. 15 Dot plot showing *Shox* expression in vertebrates. Dot size represents the percentages of cells within each cluster expressing that gene. The gradient colours from white to species colour were scaled for each gene on individual species.

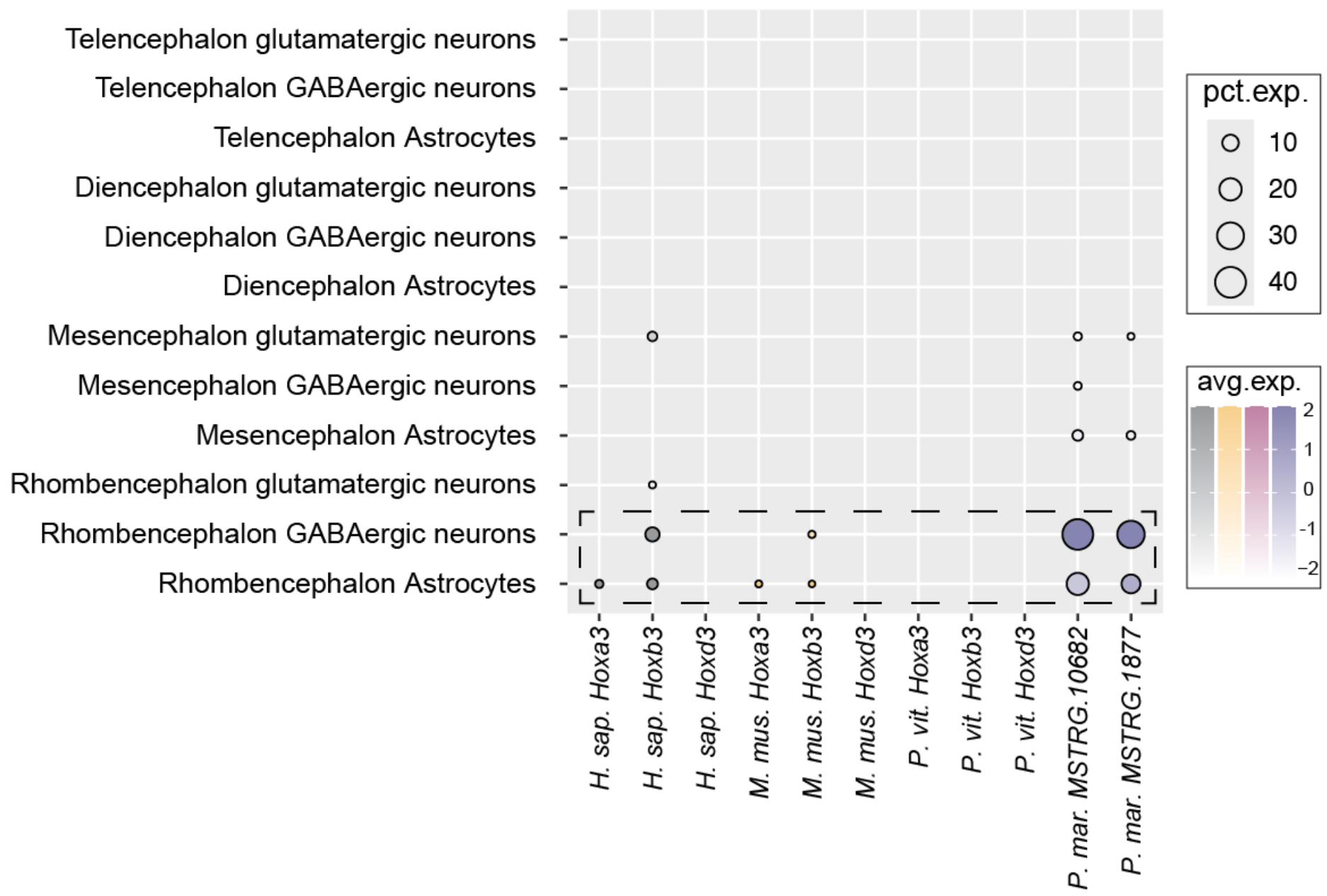

Supplementary Fig. 16 Dot plot showing *Hox3* expression in vertebrates. Dot size represents the percentages of cells within each cluster expressing that gene. The gradient colours from white to species colour were scaled for each gene on individual species.

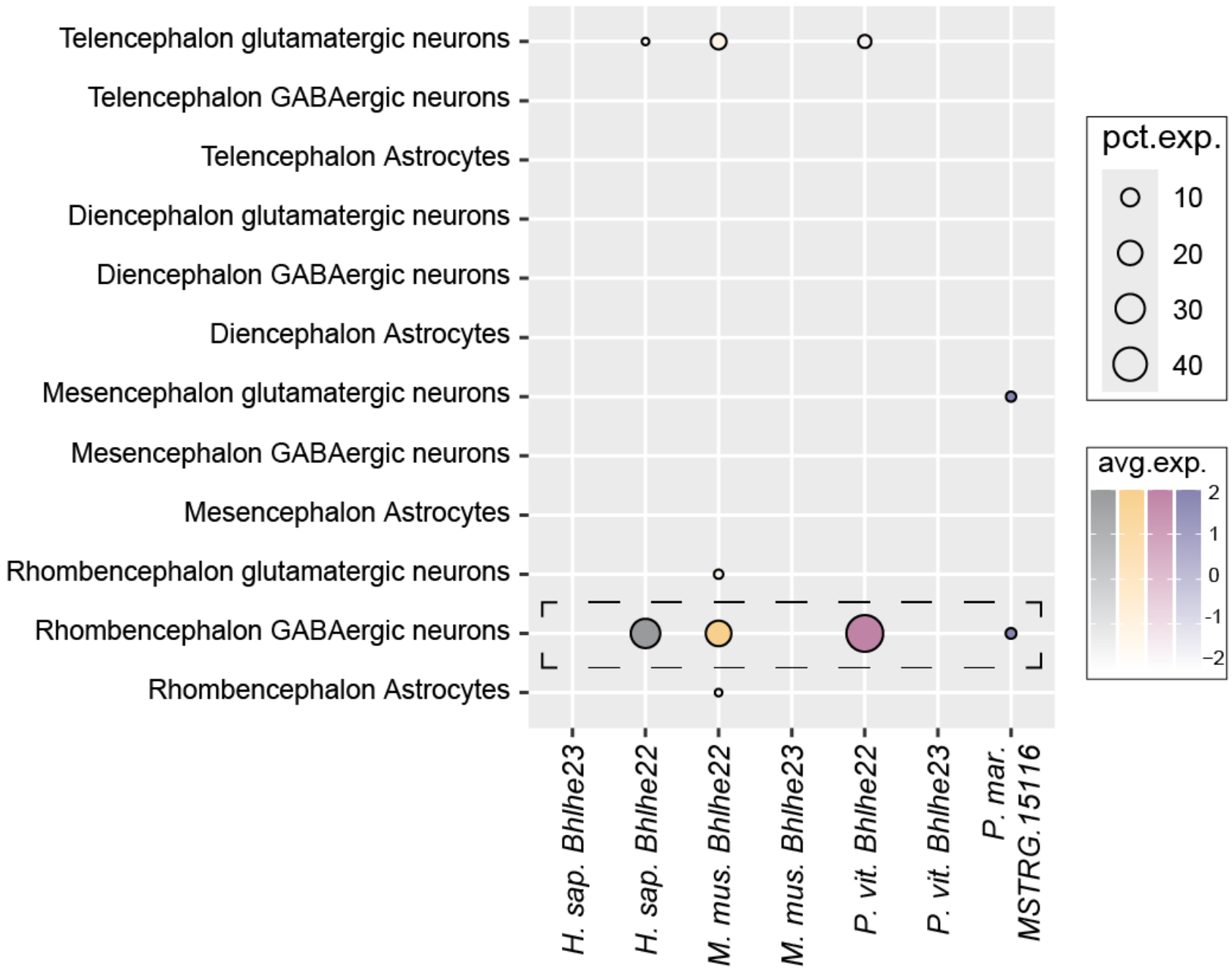

Supplementary Fig. 17 Dot plot showing *Bhlhe22/23* expression in vertebrates. Dot size represents the percentages of cells within each cluster expressing that gene. The gradient colours from white to species colour were scaled for each gene on individual species.

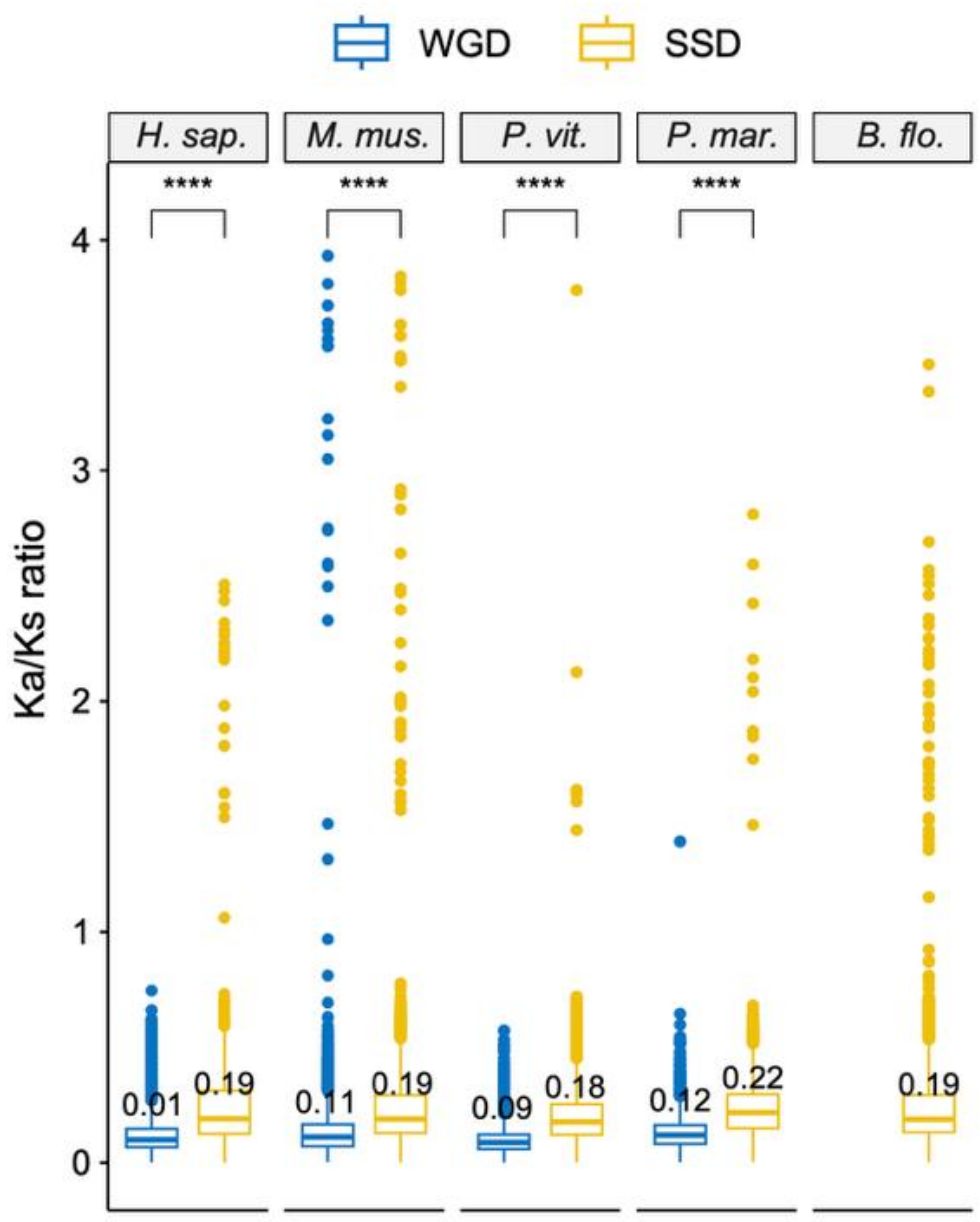

Supplementary Fig. 18 Selection on coding sequences of ohnologues and SSD paralogues. Box plots showing the distribution of Ka/Ks ratios for WGD- and SSD-derived paralogue pairs. See Methods for details of Ka/Ks calculation. Statistical significance was assessed using a two-sided Wilcoxon signed-rank test; all comparisons were significant (P < 0.0001; ****). Boxplot elements as Fig. 2a.

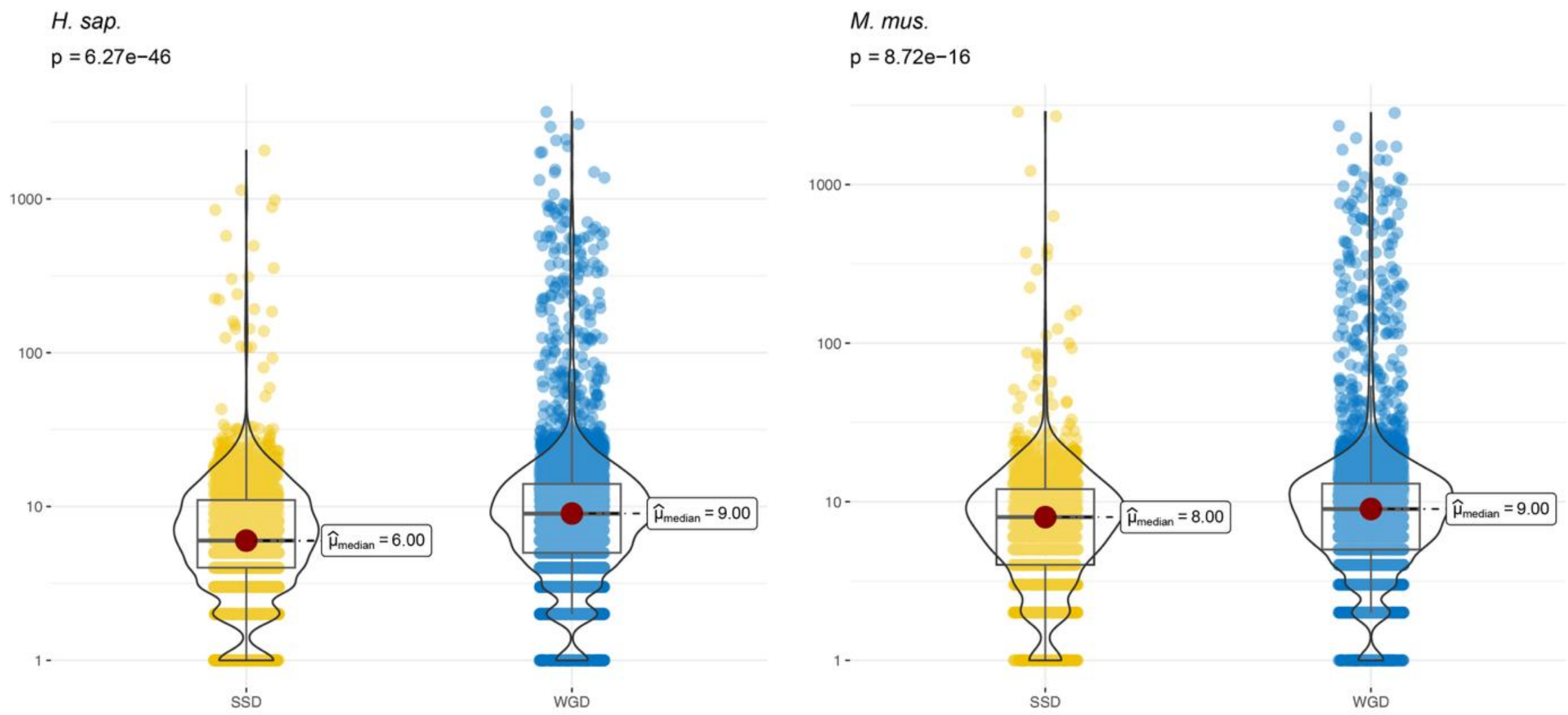

Supplementary Fig. 19 Degree (number of target genes) distribution of ohnologues and SSD paralogues. Violin plots showing the number of target-genes for WGD- and SSD-derived paralogue TFs. Statistical significance was assessed using a two-sided Wilcoxon signed-rank test; all comparisons were significant. The number shown for each violin represents the median value.

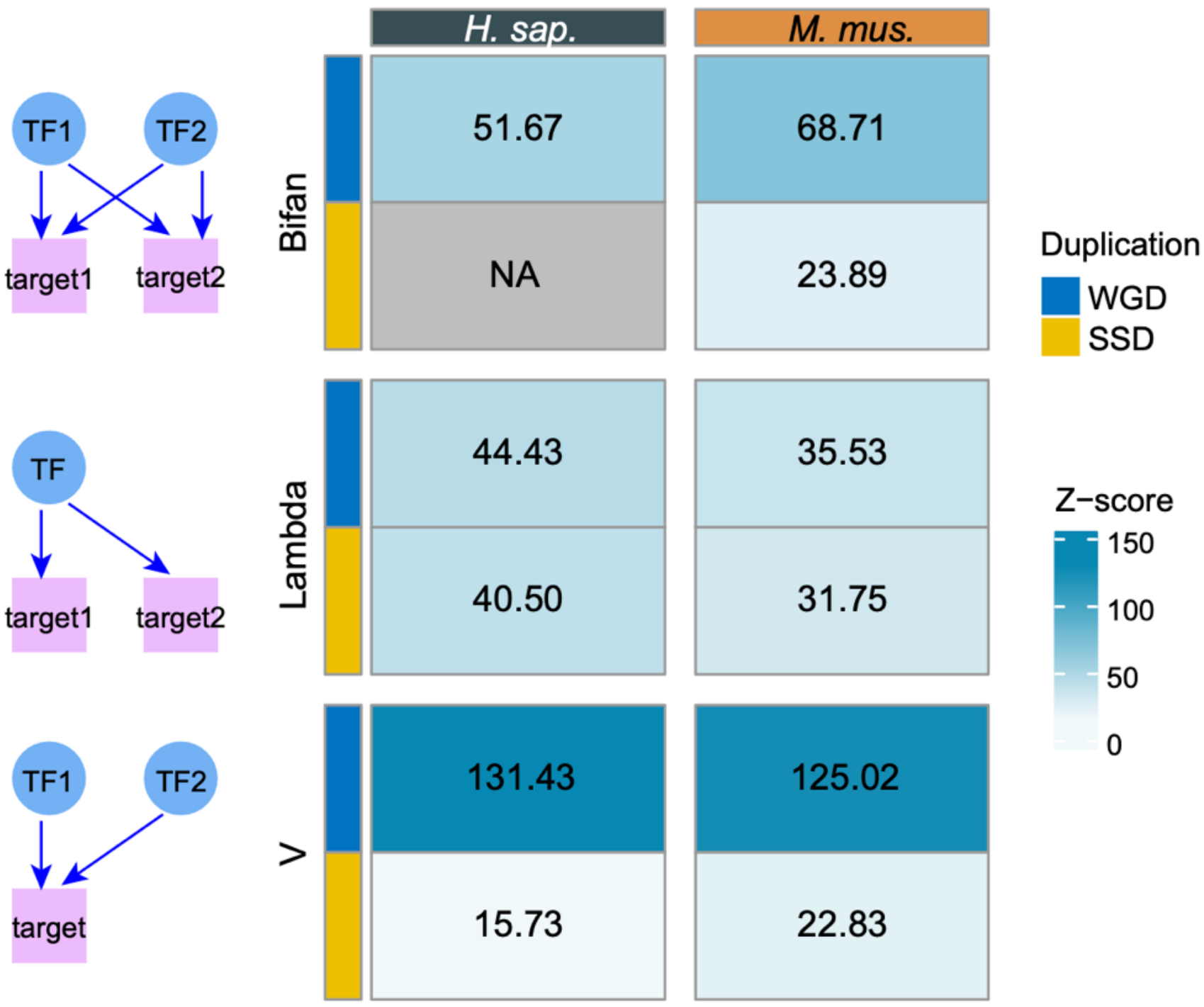

Supplementary Fig. 20 Regulatory motif enrichment for human and mouse paralogues. Three representative shapes of regulatory motifs are shown on the left. TF1 and TF2 denote duplicated TFs either from WGD or SSD. Similarly, target gene 1 and target gene 2 are duplicated genes arising from WGD or SSD. Z-scores indicate motif enrichment relative to random expectation; |Z-score| less than 2 was considered non-significant. The human SSD box in the Bifan row shows NA for not available, as there were no instances where both TF and target had duplicated such that they could be included. There were only two instances for mouse SSD paralogues in Bifan.

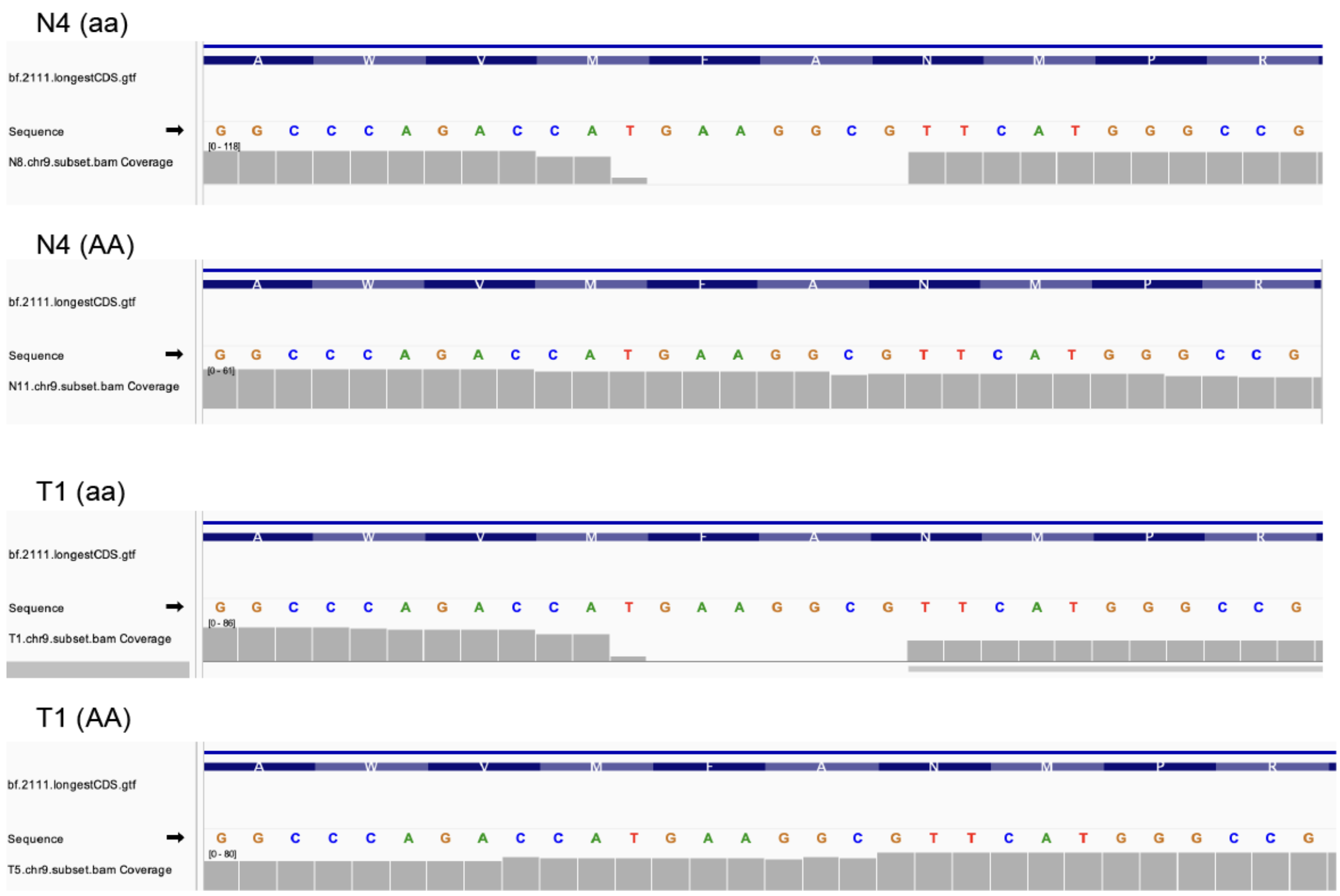

Supplementary Fig. 21 RNA-seq reads aligned by STAR were visualized in IGV to verify *SoxE* genotypes. Representative read alignments from four samples (N8, N11, T1, and T5) are shown, highlighting 8 bp deletion that distinguishes homozygous *SoxE* mutants (aa) from wild-type samples (AA). We also performed genotyping by PCR and gel electrophoresis before sequencing (Supp. Table 11 and Supp. Fig. 22).

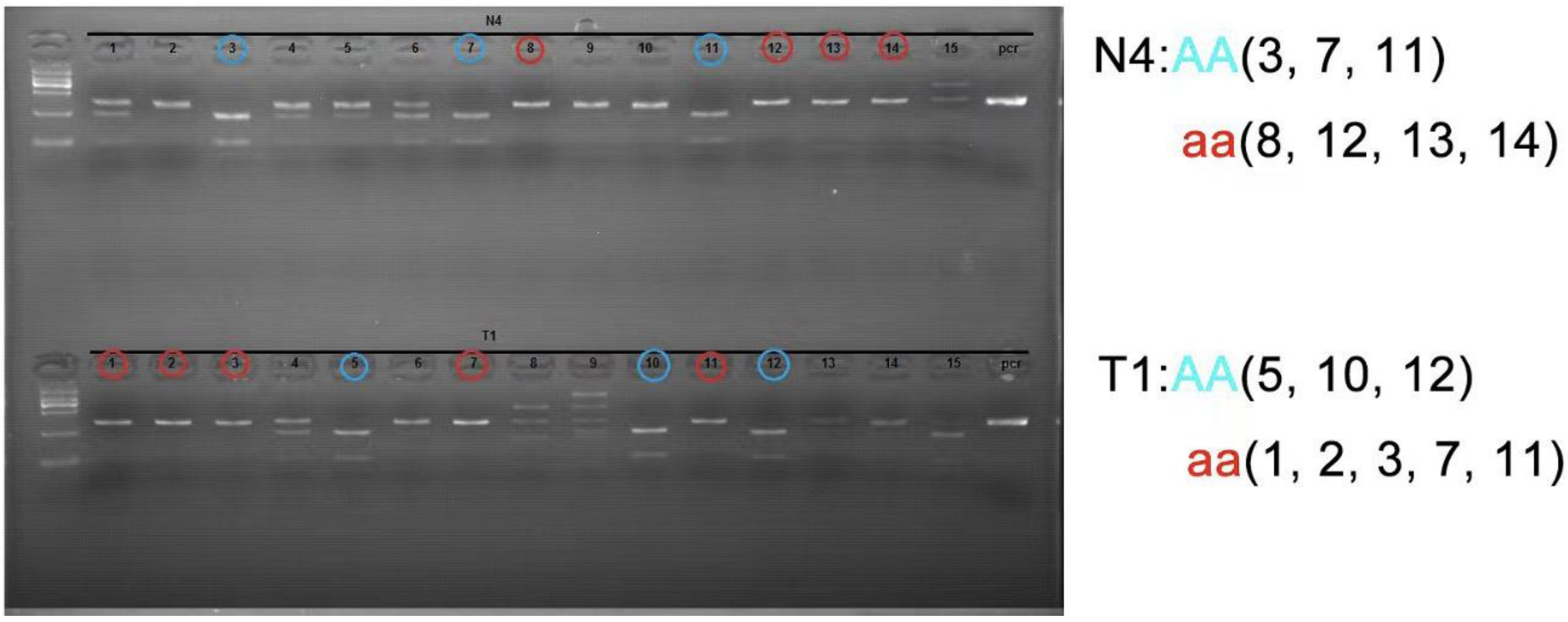

Supplementary Fig. 22 Genotyping of N4 and T1 samples by restriction enzyme digestion. Representative agarose gel electrophoresis images showing genotyping results for N4 (top) and T1 (bottom) stage embryos. Lane numbers are indicated above each gel. DNA size markers (DL2000) are shown in the leftmost lanes, and a PCR control (completely undigested) is included on the far right. Based on banding patterns, a subset of samples with wild-type (blue, AA) and homozygous SoxE mutant (red, aa) genotypes were selected for subsequent experiments, as indicated.

SI References:

1. Candiani, S., Moronti, L., Ramoino, P., Schubert, M. & Pestarino, M. A neurochemical map of the developing amphioxus nervous system. *BMC Neurosci* **13**, 59 (2012).
2. Goriely, A. Eighty-six billion and counting: do we know the number of neurons in the human brain? *Brain* **148**, 689–691 (2025).
3. Williams, R. W. Mapping Genes that Modulate Mouse Brain Development: A Quantitative Genetic Approach. in *Mouse Brain Development* (eds Goffinet, A. M. & Rakic, P.) vol. 30 21–49 (Springer Berlin Heidelberg, Berlin, Heidelberg, 2000).
4. Lemaire, L. A., Cao, C., Yoon, P. H., Long, J. & Levine, M. The hypothalamus predates the origin of vertebrates. *Sci. Adv.* **7**, eabf7452 (2021).
5. Albuixech-Crespo, B. *et al.* Molecular regionalization of the developing amphioxus neural tube challenges major partitions of the vertebrate brain. *PLoS Biol* **15**, e2001573 (2017).
6. Steuernagel, L. *et al.* HypoMap—a unified single-cell gene expression atlas of the murine hypothalamus. *Nat Metab* **4**, 1402–1419 (2022).
7. Dai, Y. *et al.* Evolutionary origin of the chordate nervous system revealed by amphioxus developmental trajectories. *Nat Ecol Evol* https://doi.org/10.1038/s41559-024-02469-7 (2024) doi:10.1038/s41559-024-02469-7.
8. Cao, C. *et al.* Comprehensive single-cell transcriptome lineages of a proto-vertebrate. *Nature* **571**, 349–354 (2019).
9. Horie, T. *et al.* Regulatory cocktail for dopaminergic neurons in a protovertebrate identified by whole-embryo single-cell transcriptomics. *Genes Dev.* **32**, 1297–1302 (2018).
10. Tarashansky, A. J. *et al.* Mapping single-cell atlases throughout Metazoa unravels cell type evolution. *eLife* **10**, e66747 (2021).
11. Corrigan, E. K. *et al.* Conservation and alteration of mammalian striatal interneurons. *Nature* **647**, 187–193 (2025).
12. Almeida-Silva, F. & Van De Peer, Y. *doubletrouble* : an R/Bioconductor package for the identification, classification, and analysis of gene and genome duplications. *Bioinformatics* **41**, btaf043 (2025).
13. Qiao, X. *et al.* Gene duplication and evolution in recurring polyploidization–diploidization cycles in plants. *Genome Biol* **20**, 38 (2019).
14. Singh, P. P. & Isambert, H. OHNOLOGS v2: a comprehensive resource for the genes retained from whole genome duplication in vertebrates. *Nucleic Acids Research* gkz909 (2019) doi:10.1093/nar/gkz909.
15. Marlétaz, F. *et al.* The hagfish genome and the evolution of vertebrates. *Nature* **627**, 811–820 (2024).
16. Lamanna, F. *et al.* A lamprey neural cell type atlas illuminates the origins of the vertebrate brain. *Nat Ecol Evol* https://doi.org/10.1038/s41559-023-02170-1 (2023) doi:10.1038/s41559-023-02170-1.
17. Szklarczyk, D. *et al.* The STRING database in 2023: protein–protein association networks and functional enrichment analyses for any sequenced genome of interest. *Nucleic Acids Research* **51**, D638–D646 (2023).
18. Veitia, R. A., Bottani, S. & Birchler, J. A. Cellular reactions to gene dosage imbalance: genomic, transcriptomic and proteomic effects. *Trends in Genetics* **24**, 390–397 (2008).
19. Almeida-Silva, F. Whole-genome Duplications and the Long-term Evolution of Gene Regulatory Networks in Angiosperms.
20. Metzis, V. *et al.* Nervous System Regionalization Entails Axial Allocation before Neural Differentiation. *Cell* **175**, 1105-1118.e17 (2018).
21. Jessell, T. M. Neuronal specification in the spinal cord: inductive signals and transcriptional codes. *Nat Rev Genet* **1**, 20–29 (2000).

22. Mayer, C. *et al.* Developmental diversification of cortical inhibitory interneurons. *Nature* **555**, 457–462 (2018).
23. Herrero-Navarro, Á. *et al.* Astrocytes and neurons share region-specific transcriptional signatures that confer regional identity to neuronal reprogramming. *Sci. Adv.* **7**, eabe8978 (2021).
24. Martynoga, B., Morrison, H., Price, D. J. & Mason, J. O. Foxg1 is required for specification of ventral telencephalon and region-specific regulation of dorsal telencephalic precursor proliferation and apoptosis. *Developmental Biology* **283**, 113–127 (2005).
25. Shimizu, T. *et al.* Zinc finger genes *Fezf1* and *Fezf2* control neuronal differentiation by repressing *Hes5* expression in the forebrain. *Development* **137**, 1875–1885 (2010).
26. Yoshida, M. *et al. Emx1* and *Emx2* functions in development of dorsal telencephalon. *Development* **124**, 101–111 (1997).
27. Karalay, Ö. *et al.* Prospero-related homeobox 1 gene (Prox1) is regulated by canonical Wnt signaling and has a stage-specific role in adult hippocampal neurogenesis. *Proc. Natl. Acad. Sci. U.S.A.* **108**, 5807–5812 (2011).
28. Lipiec, M. A. *et al.* TCF7L2 regulates postmitotic differentiation programs and excitability patterns in the thalamus. *Development* dev.190181 (2020) doi:10.1242/dev.190181.
29. Lavado, A., Lagutin, O. V. & Oliver, G. *Six3* inactivation causes progressive caudalization and aberrant patterning of the mammalian diencephalon. *Development* **135**, 441–450 (2008).
30. Vernay, B. *et al.* Otx2 Regulates Subtype Specification and Neurogenesis in the Midbrain. *J. Neurosci.* **25**, 4856–4867 (2005).
31. Sgaier, S. K. *et al.* Genetic subdivision of the tectum and cerebellum into functionally related regions based on differential sensitivity to engrailed proteins. *Development* **134**, 2325–2335 (2007).
32. Thompson, J. A., Zembrzycki, A., Mansouri, A. & Ziman, M. Pax7 is requisite for maintenance of a subpopulation of superior collicular neurons and shows a diverging expression pattern to Pax3 during superior collicular development. *BMC Dev Biol* **8**, 62 (2008).
33. Boudjadi, S., Chatterjee, B., Sun, W., Vemu, P. & Barr, F. G. The expression and function of PAX3 in development and disease. *Gene* **666**, 145–157 (2018).
34. Aruga, J. & Millen, K. J. ZIC1 Function in Normal Cerebellar Development and Human Developmental Pathology. in *Zic family* (ed. Aruga, J.) vol. 1046 249–268 (Springer Singapore, Singapore, 2018).
35. Dai, Y. *et al.* Single-cell profiling of the amphioxus digestive tract reveals conservation of endocrine cells in chordates. *Sci. Adv.* **10**, eadq0702 (2024).
36. Ryota Suzuki <suzuki@ef-prime.com>, Yoshikazu Terada <terada@sigmath.es.osaka-u.ac.jp>, Hidetoshi Shimodaira <shimo@i.kyoto-u.ac.jp>. pvclust: Hierarchical Clustering with P-Values via Multiscale Bootstrap Resampling. 2.2–0 https://doi.org/10.32614/CRAN.package.pvclust (2005).
37. Love, M. I., Huber, W. & Anders, S. Moderated estimation of fold change and dispersion for RNA-seq data with DESeq2. *Genome Biol* **15**, 550 (2014).
