# Supplementary material for: Whole-genome duplication shaped cell-type evolution in the vertebrate brain
Source: Nature. 2026 Jun 10;656(8128):659–69. doi: 10.1038/s41586-026-10629-x (PMC13489949; doi:10.1038/s41586-026-10629-x)
Supplement: Supplementary file 3 [file 41586_2026_10629_MOESM2_ESM.pdf]

nature portfolio

Corresponding author(s): Yuanzhen Zhu, Guang Li, Sebastian M. Shimeld

Last updated by author(s): Apr 21, 2026

Reporting Summary

Nature Portfolio wishes to improve the reproducibility of the work that we publish. This form provides structure for consistency and transparency in reporting. For further information on Nature Portfolio policies, see our Editorial Policies and the Editorial Policy Checklist.

Statistics

For all statistical analyses, confirm that the following items are present in the figure legend, table legend, main text, or Methods section.

n/a	Confirmed	
☐	☒	The exact sample size (*n*) for each experimental group/condition, given as a discrete number and unit of measurement
☒	☐	A statement on whether measurements were taken from distinct samples or whether the same sample was measured repeatedly
☐	☒	The statistical test(s) used AND whether they are one- or two-sided<br>*Only common tests should be described solely by name; describe more complex techniques in the Methods section.*
☒	☐	A description of all covariates tested
☐	☒	A description of any assumptions or corrections, such as tests of normality and adjustment for multiple comparisons
☐	☒	A full description of the statistical parameters including central tendency (e.g. means) or other basic estimates (e.g. regression coefficient) AND variation (e.g. standard deviation) or associated estimates of uncertainty (e.g. confidence intervals)
☐	☒	For null hypothesis testing, the test statistic (e.g. *F*, *t*, *r*) with confidence intervals, effect sizes, degrees of freedom and *P* value noted<br>*Give P values as exact values whenever suitable.*
☒	☐	For Bayesian analysis, information on the choice of priors and Markov chain Monte Carlo settings
☒	☐	For hierarchical and complex designs, identification of the appropriate level for tests and full reporting of outcomes
☐	☒	Estimates of effect sizes (e.g. Cohen's *d*, Pearson's *r*), indicating how they were calculated

Our web collection on statistics for biologists contains articles on many of the points above.

Software and code

Policy information about availability of computer code

Data collection	BD Rhapsody WTA Analysis Pipeline v1.12.1 and SeekSoul Tools
Data analysis	Software used to analyze the data were described in detail in the Methods section of the manuscript. Relevant code and resources have been uploaded to Figshare (private link: https://doi.org/10.6084/m9.figshare.29327111). All scripts available in GitHub: https://github.com/DiracZhu1998/WGD2celltype_evolution/tree/main. All environment yml files available in the above GitHub.<br><br>Other software used:<br>Seurat v5.0.0<br>SAMap v1.0.15<br>scrattch.hicat / scrattch.bigcat v0.1.0<br>clusterProfiler v4<br>AnnotationForge v1.44<br>OrthoFinder v2.5.5<br>MAFFT v7.520<br>PAL2NAL v14<br>KaKs_Calculator v2.0<br>pySCENIC v0.11.2<br>velocyto.py v0.17 and scVelo v0.3.3<br>DESeq2 v1.42.0<br>STAR v2.4.0g1<br>featureCounts v2.1.1

fastp v1.0.1
pvclust v2.2.0
lme4 v1.1.35.6
igraph v2.0.3
rstatix v0.7.2

For manuscripts utilizing custom algorithms or software that are central to the research but not yet described in published literature, software must be made available to editors and reviewers. We strongly encourage code deposition in a community repository (e.g. GitHub). See the Nature Portfolio guidelines for submitting code & software for further information.

Data

Policy information about availability of data

All manuscripts must include a data availability statement. This statement should provide the following information, where applicable:
- Accession codes, unique identifiers, or web links for publicly available datasets
- A description of any restrictions on data availability
- For clinical datasets or third party data, please ensure that the statement adheres to our policy

The reference genome, gene models, functional annotations of protein-coding genes, full marker gene list of each cell cluster, final atlases, and important intermediate files are deposited in the figshare repository under the link https://doi.org/10.6084/m9.figshare.29327111.
Data analysed from publicly available sources are described in the paper and GitHub/Figshare, and listed below:
1). Human brain atlas (human_adult_GRCh38-3.0.0.h5ad) from: https://github.com/linnarsson-lab/adult-human-brain
2). Mouse brain atlas (l5_all.loom) from: http://mousebrain.org/adolescent/downloads.html
3). Lizard brain atlas (Pogona_vitticeps_cells_Science_2022.rds) from: https://datashare.mpcdf.mpg.de/s/WBX59YhJKKebizb#editor
4). Lamprey brain atlases (adult_diencephalon.rds, adult_rhombencephalon.rds, adult_mesencephalon.rds, adult_telencephalon.rds, lamprey_adult_whole_brain.rds) from: https://downloads.kaessmannlab.org/lamprey/
5). The human eye and lung scRNA were pre-processed by Human Cell Atlas and downloaded from following links (https://datasets.cellxgene.cziscience.com/64175889-d600-4b58-97ea-e74be80206e5.rds and https://datasets.cellxgene.cziscience.com/b351804c-293e-4aeb-9c4c-043db67f4540.rds).
6). Three species cerebellar nuclei datasets (GSM4873765_mouse_data.RData.gz, GSM4873766_human_data.RData.gz, GSM4873767_chicken_data.RData.gz) were downloaded from: https://www.ncbi.nlm.nih.gov/geo/query/acc.cgi?acc=GSE160471
7). Ohnologs v2 database: http://ohnologs.curie.fr

Research involving human participants, their data, or biological material

Policy information about studies with human participants or human data. See also policy information about sex, gender (identity/presentation), and sexual orientation and race, ethnicity and racism.

Reporting on sex and gender	N/A
Reporting on race, ethnicity, or other socially relevant groupings	N/A
Population characteristics	N/A
Recruitment	N/A
Ethics oversight	N/A

Note that full information on the approval of the study protocol must also be provided in the manuscript.

Field-specific reporting

Please select the one below that is the best fit for your research. If you are not sure, read the appropriate sections before making your selection.

☒ Life sciences ☐ Behavioural & social sciences ☐ Ecological, evolutionary & environmental sciences

For a reference copy of the document with all sections, see nature.com/documents/nr-reporting-summary-flat.pdf

Life sciences study design

All studies must disclose on these points even when the disclosure is negative.

Sample size	No statistical methods were used to predetermine species sample size. We selected 5 lineages for analysis: human, mouse, lizard, lamprey and amphioxus. We chose these because they capture the relevant spread of chordate lineages.<br><br>Data for the four vertebrate species were from published studies which determined the number of sequenced cells available for analysis for these. We also generated new adult amphioxus brain and neural tube datasets and a new lamprey embryo spinal cord dataset. The number of cells sequenced was consistent with previous studies and sufficient to capture known rare cell types such as those in the infundibulum.

For bulk RNA-seq, we selected three biological replicates per condition , which is standard for differential gene expression analysis.

Data exclusions	No lineages were subsequently excluded from the analyses. Human and lizard datasets were randomly downsampled to produce datasets more comparable in size to datasets in other species. Details are included in the manuscript.
Replication	Replication within individual vertebrate species is as described in the publications describing these datasets. These are cited in the manuscript. Sequencing datasets and experiments we performed were based on biological replicates. All replication attempts were successful, and results were consistent across replicates.
Randomization	Not applicable; For mutant analyses, samples were selected based on genotype rather than randomized assignment.
Blinding	Not applicable; No blinding was performed, as the study primarily involved computational analyses of sequencing data. For experimental procedures, samples were processed and analysed without blinding, as no subjective outcome assessment was involved.

Reporting for specific materials, systems and methods

We require information from authors about some types of materials, experimental systems and methods used in many studies. Here, indicate whether each material, system or method listed is relevant to your study. If you are not sure if a list item applies to your research, read the appropriate section before selecting a response.

Materials & experimental systems

n/a	Involved in the study	
☒	☐	Antibodies
☒	☐	Eukaryotic cell lines
☒	☐	Palaeontology and archaeology
☐	☒	Animals and other organisms
☒	☐	Clinical data
☒	☐	Dual use research of concern
☒	☐	Plants

Methods

n/a	Involved in the study	
☒	☐	ChIP-seq
☒	☐	Flow cytometry
☒	☐	MRI-based neuroimaging

Animals and other research organisms

Policy information about studies involving animals; ARRIVE guidelines recommended for reporting animal research, and Sex and Gender in Research

Laboratory animals	Amphioxus were housed in a temperature-controlled facility at Xiamen University and were obtained obtained from a stock maintained by Dr. Jr-Kai Yu, originating from Tampa, Florida. Developmental stages (N4, T1) are specified. No strain name is applicable. No other laboratory animal species were directly used in this study. Some analyses incorporated publicly available datasets derived from laboratory animals; details regarding these datasets can be found in the cited publications.
Wild animals	Lamprey embryos (Lampetra planeri) were collected from Highland Water in the New Forest, UK, under permit from Forestry England.
Reporting on sex	Lamprey experiments were conducted on embryos in which sexual differentiation (which is not thought to be genetically determined) has not occurred. Amphioxus embryos do not show sexual differentiation and sex was not recorded. Adult amphioxus RNAseq libraries were derived from tissue from multiple individuals (as described in the Methods) of both sexes.
Field-collected samples	As above under wild animals
Ethics oversight	Work on lamprey embryos was approved by the University of Oxford, Department of Zoology Animal Welfare and Ethical Review Board. No ethical approval or guidance was required for amphioxus.

Note that full information on the approval of the study protocol must also be provided in the manuscript.

Plants

Seed stocks	*Report on the source of all seed stocks or other plant material used. If applicable, state the seed stock centre and catalogue number. If plant specimens were collected from the field, describe the collection location, date and sampling procedures.*
Novel plant genotypes	*Describe the methods by which all novel plant genotypes were produced. This includes those generated by transgenic approaches, gene editing, chemical/radiation-based mutagenesis and hybridization. For transgenic lines, describe the transformation method, the number of independent lines analyzed and the generation upon which experiments were performed. For gene-edited lines, describe the editor used, the endogenous sequence targeted for editing, the targeting guide RNA sequence (if applicable) and how the editor was applied.*
Authentication	*Describe any authentication procedures for each seed stock used or novel genotype generated. Describe any experiments used to assess the effect of a mutation and, where applicable, how potential secondary effects (e.g. second site T-DNA insertions, mosiacism, off-target gene editing) were examined.*
